# Supplementary material for: Cell-Type-Specific Profiling of the Arabidopsis thaliana Membrane Protein-Encoding Genes
Source: Membranes (Basel). 2022 Sep 10;12(9):874. doi: 10.3390/membranes12090874 (PMC9506093; doi:10.3390/membranes12090874)
Supplement: Supplementary file 1 [file membranes-12-00874-s001.zip › membranes-1909363-supplementary.pdf]

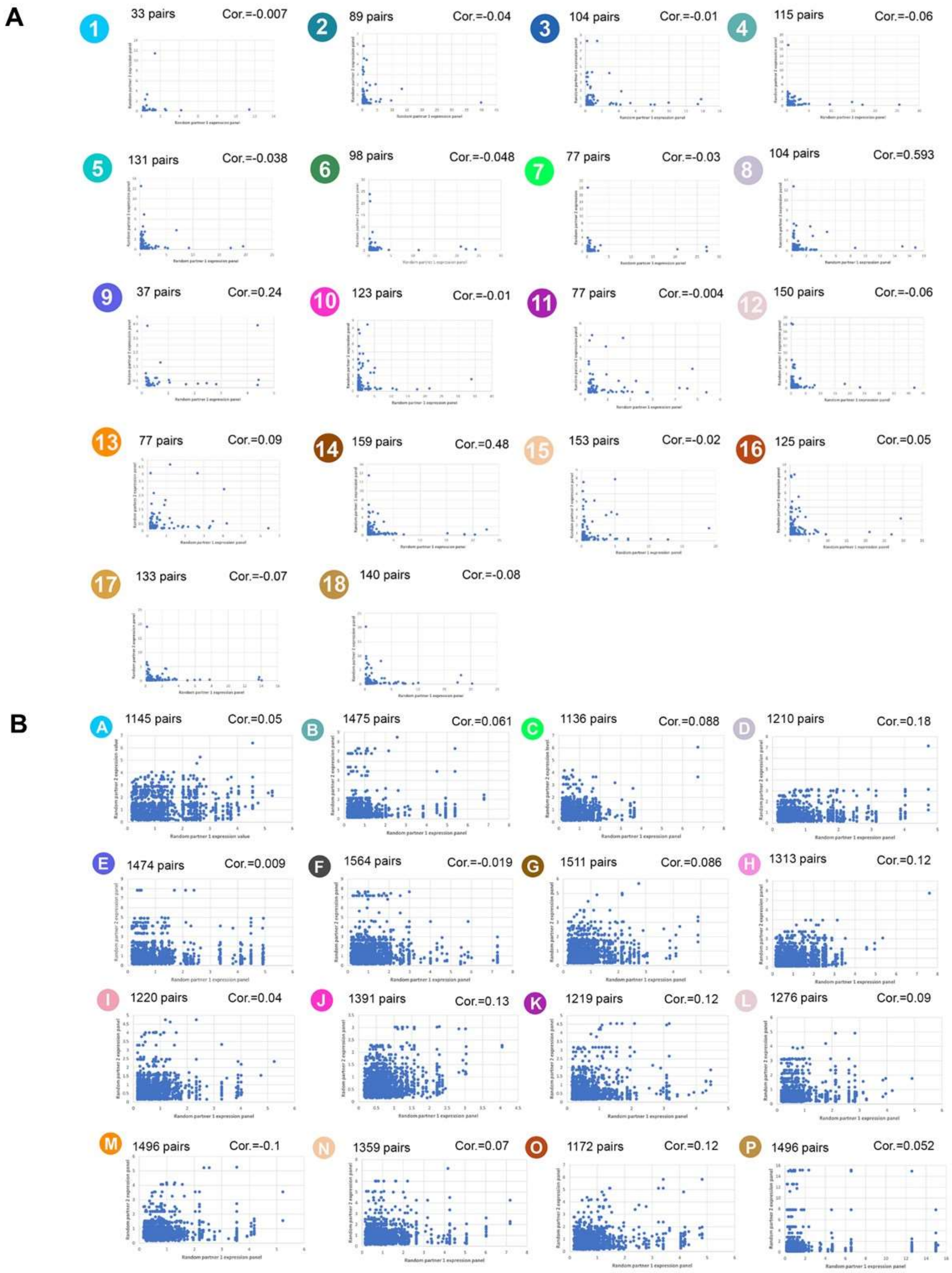

Figure S1. Correlation analysis of the expression of 1,752 pairs of Arabidopsis genes randomly selected. For each protoplast- (A, clusters 1 to 18) and nuclei-based cluster (B, clusters A to P), the average numbers of UMIs per protoplast/nuclei of each gene composing the pair are indicated on the x-axis and y-axis. For each cluster, The total number of pairs with both genes expressed is indicated as well as the coefficient of correlation between the expression of the two genes.

Figure S1: Correlation analysis of the expression of 1752 pairs of Arabidopsis genes randomly selected

Table S1. List of 1,752 Arabidopsis membrane pairs of genes analyzed as the high-confidence interaction of their encoded protein [6] and used in this study to test the co-expression at single-cell level.

Partner 1 ID	Partner 1 Name	Partner 2 ID	Partner 2 Name
AT1G01100		AT5G47700	
AT1G03860	ATPHB2	AT2G20530	ATPHB6
AT1G03860	ATPHB2	AT3G03060	
AT1G03860	ATPHB2	AT5G05370	
AT1G03860	ATPHB2	AT3G27280	ATPHB4
AT1G03860	ATPHB2	AT3G44330	
AT1G03860	ATPHB2	AT3G62360	
AT1G03860	ATPHB2	AT4G12590	
AT1G03860	ATPHB2	AT4G27585	
AT1G03860	ATPHB2	AT4G28510	ATPHB1
AT1G03860	ATPHB2	AT5G25450	
AT1G03860	ATPHB2	AT5G40770	ATPHB3
AT1G03860	ATPHB2	AT5G46800	BOU
AT1G04280		AT3G03060	
AT1G04630	MEE4	AT1G15210	ABCG35
AT1G04630	MEE4	AT1G16700	
AT1G04630	MEE4	AT1G47260	APFI
AT1G04630	MEE4	AT1G51980	
AT1G04630	MEE4	AT1G63500	
AT1G04630	MEE4	AT1G67350	
AT1G04630	MEE4	AT1G79010	
AT1G04630	MEE4	AT2G07698	
AT1G04630	MEE4	AT2G18730	ATDGK3
AT1G04630	MEE4	AT2G26140	ftsh4
AT1G04630	MEE4	AT2G36850	ATGSL08
AT1G04630	MEE4	AT3G01290	AtHIR2
AT1G04630	MEE4	AT3G02090	MPPBETA
AT1G04630	MEE4	AT3G03100	
AT1G04630	MEE4	AT3G07160	ATGSL10
AT1G04630	MEE4	AT3G18410	
AT1G04630	MEE4	AT3G42050	
AT1G04630	MEE4	AT3G48680	AtCAL2
AT1G04630	MEE4	AT3G51550	FER
AT1G04630	MEE4	AT3G53480	ABCG37
AT1G04630	MEE4	AT4G02580	
AT1G04630	MEE4	AT4G16450	
AT1G04630	MEE4	AT4G32470	
AT1G04630	MEE4	AT5G08060	
AT1G04630	MEE4	AT5G08530	CI51
AT1G04630	MEE4	AT5G11770	
AT1G04630	MEE4	AT5G13450	ATP5
AT1G04630	MEE4	AT5G18800	
AT1G04630	MEE4	AT5G37510	CI76
AT1G04630	MEE4	AT5G52840	
AT1G04630	MEE4	AT5G66510	GAMMA CA3
AT1G04630	MEE4	ATMG00070	NAD9
AT1G05150		AT3G62830	ATUXS2
AT1G05150		AT4G00710	BSK3

AT1G05150		AT4G17140	
AT1G05150		AT5G40890	ATCLC-A
AT1G05570	ATGSL06	AT3G28720	
AT1G05570	ATGSL06	AT5G40890	ATCLC-A
AT1G06840		AT1G15210	ABCG35
AT1G06840		AT3G08580	AAC1
AT1G06840		AT3G42050	
AT1G06840		AT3G57330	ACA11
AT1G06840		AT5G49760	
AT1G06950	ATTIC110	AT1G12110	ATNRT1
AT1G06950	ATTIC110	AT1G18260	EBS5
AT1G06950	ATTIC110	AT1G20330	CVP1
AT1G06950	ATTIC110	AT1G76400	
AT1G06950	ATTIC110	AT2G21160	
AT1G06950	ATTIC110	AT2G30490	ATC4H
AT1G06950	ATTIC110	AT2G40890	CYP98A3
AT1G06950	ATTIC110	AT3G20940	CYP705A30
AT1G06950	ATTIC110	AT3G52190	AtPHF1
AT1G06950	ATTIC110	AT4G00900	ATECA2
AT1G06950	ATTIC110	AT4G14070	AAE15
AT1G06950	ATTIC110	AT5G50460	
AT1G06950	ATTIC110	AT4G29130	ATHXK1
AT1G06950	ATTIC110	AT4G31500	ATR4
AT1G06950	ATTIC110	AT4G35230	BSK1
AT1G06950	ATTIC110	AT4G38350	
AT1G06950	ATTIC110	AT5G18480	PGSIP6
AT1G06950	ATTIC110	AT5G20660	
AT1G06950	ATTIC110	AT5G27540	emb2473
AT1G06950	ATTIC110	AT5G40510	
AT1G06950	ATTIC110	AT5G42570	
AT1G06950	ATTIC110	AT5G42590	CYP71A16
AT1G06950	ATTIC110	AT5G52240	AtMAPR5
AT1G06950	ATTIC110	ATMG00160	COX2
AT1G07670	ATECA4	AT1G15500	ATNTT2
AT1G07670	ATECA4	AT1G53000	AtCKS
AT1G07670	ATECA4	AT1G76400	
AT1G07670	ATECA4	AT2G17720	P4H5
AT1G07670	ATECA4	AT2G45060	
AT1G07670	ATECA4	AT2G45140	PVA12
AT1G07670	ATECA4	AT3G01280	ATVDAC1
AT1G07670	ATECA4	AT3G14680	CYP72A14
AT1G07670	ATECA4	AT3G47833	SDH7
AT1G07670	ATECA4	AT3G54110	ATPUMP1
AT1G07670	ATECA4	AT3G60600	(AT)VAP
AT1G07670	ATECA4	AT4G14070	AAE15
AT1G07670	ATECA4	AT4G21150	HAP6
AT1G07670	ATECA4	AT4G24520	AR1
AT1G07670	ATECA4	AT4G31500	ATR4
AT1G07670	ATECA4	AT5G15090	ATVDAC3
AT1G07670	ATECA4	AT5G57490	ATVDAC4
AT1G07670	ATECA4	AT5G66680	DGL1
AT1G07810	ACA3	AT1G10130	ATECA3

AT1G07810	ACA3	AT1G18260	EBS5
AT1G07810	ACA3	AT1G59870	ABCG36
AT1G07810	ACA3	AT2G36380	ABCG34
AT1G07810	ACA3	AT3G07570	
AT1G07810	ACA3	AT5G50460	
AT1G07810	ACA3	AT5G14040	PHT3;1
AT1G07810	ACA3	AT5G18480	PGSIP6
AT1G07810	ACA3	AT5G27540	emb2473
AT1G07810	ACA3	AT5G52240	AtMAPR5
AT1G08930	ERD6	AT1G18260	EBS5
AT1G08930	ERD6	AT1G55850	ATCSLE1
AT1G08930	ERD6	AT2G01470	ATSEC12
AT1G08930	ERD6	AT3G05970	ATLACS6
AT1G08930	ERD6	AT4G01320	ATSTE24
AT1G08930	ERD6	AT4G26410	
AT1G08930	ERD6	AT5G08080	ATSYP132
AT1G08930	ERD6	AT5G18480	PGSIP6
AT1G08930	ERD6	AT5G25100	
AT1G08930	ERD6	AT5G36220	CYP81D1
AT1G08930	ERD6	AT5G58270	ABCB25
AT1G10130	ATECA3	AT3G05970	ATLACS6
AT1G10130	ATECA3	AT3G07570	
AT1G10130	ATECA3	AT3G27570	
AT1G10130	ATECA3	AT3G57010	
AT1G10130	ATECA3	AT4G29130	ATHXK1
AT1G10130	ATECA3	AT4G35230	BSK1
AT1G10130	ATECA3	AT5G18480	PGSIP6
AT1G10130	ATECA3	AT5G25100	
AT1G11260	ATSTP1	AT1G61250	SC3
AT1G11680	CYP51	AT2G21160	
AT1G11890	ATSEC22	AT1G12110	ATNRT1
AT1G11890	ATSEC22	AT5G12470	
AT1G11910	APA1	AT1G74020	SS2
AT1G11910	APA1	AT4G23430	AtTic32-IVa
AT1G11910	APA1	AT5G42650	AOS
AT1G12110	ATNRT1	AT1G55850	ATCSLE1
AT1G12110	ATNRT1	AT1G76400	
AT1G12110	ATNRT1	AT2G19080	
AT1G12110	ATNRT1	AT4G15330	CYP705A1
AT1G12110	ATNRT1	AT5G40510	
AT1G13060	PBE1	AT1G16470	PAB1
AT1G13060	PBE1	AT2G05840	PAA2
AT1G13060	PBE1	AT3G22110	PAC1
AT1G13060	PBE1	AT3G27430	PBB1
AT1G13060	PBE1	AT3G60820	PBF1
AT1G13060	PBE1	AT4G31300	PBA1
AT1G13060	PBE1	AT5G42790	ARS5
AT1G13060	PBE1	AT5G56500	Cpn60beta3
AT1G13080	CYP71B2	AT2G21160	
AT1G13080	CYP71B2	AT2G24180	CYP71B6
AT1G13080	CYP71B2	AT2G30490	ATC4H
AT1G13080	CYP71B2	AT3G13772	AtTMN7

AT1G13080	CYP71B2	AT3G26210	CYP71B23
AT1G13080	CYP71B2	AT3G61070	PEX11E
AT1G13080	CYP71B2	AT4G35000	APX3
AT1G13080	CYP71B2	AT5G12470	
AT1G14080	ATFUT6	AT4G23640	ATKT3
AT1G14830	ADL1C	AT1G59610	ADL3
AT1G15210	ABCG35	AT1G26850	
AT1G15210	ABCG35	AT1G30360	ERD4
AT1G15210	ABCG35	AT1G47260	APFI
AT1G15210	ABCG35	AT1G51980	
AT1G15210	ABCG35	AT1G63500	
AT1G15210	ABCG35	AT1G67350	
AT1G15210	ABCG35	AT1G79010	
AT1G15210	ABCG35	AT2G18730	ATDGK3
AT1G15210	ABCG35	AT2G20360	
AT1G15210	ABCG35	AT2G21410	VHA-A2
AT1G15210	ABCG35	AT2G28520	VHA-A1
AT1G15210	ABCG35	AT3G01290	AtHIR2
AT1G15210	ABCG35	AT3G02090	MPPBETA
AT1G15210	ABCG35	AT3G27240	
AT1G15210	ABCG35	AT3G28710	
AT1G15210	ABCG35	AT3G42050	
AT1G15210	ABCG35	AT3G48680	AtCAL2
AT1G15210	ABCG35	AT3G57330	ACA11
AT1G15210	ABCG35	AT4G29900	ACA10
AT1G15210	ABCG35	AT5G08530	CI51
AT1G15210	ABCG35	AT5G11770	
AT1G15210	ABCG35	AT5G13450	ATP5
AT1G15210	ABCG35	AT5G19760	
AT1G15210	ABCG35	AT5G37510	CI76
AT1G15210	ABCG35	AT5G40810	
AT1G15210	ABCG35	AT5G49760	
AT1G15210	ABCG35	AT5G51570	
AT1G15210	ABCG35	AT5G52840	
AT1G15210	ABCG35	AT5G57110	ACA8
AT1G15210	ABCG35	AT5G64030	
AT1G15210	ABCG35	AT5G66510	GAMMA CA3
AT1G15210	ABCG35	ATMG00070	NAD9
AT1G15500	ATNTT2	AT1G19110	
AT1G15500	ATNTT2	AT1G53000	AtCKS
AT1G15500	ATNTT2	AT1G61790	
AT1G15500	ATNTT2	AT2G17720	P4H5
AT1G15500	ATNTT2	AT2G45140	PVA12
AT1G15500	ATNTT2	AT3G27820	ATMDAR4
AT1G15500	ATNTT2	AT4G21150	HAP6
AT1G15500	ATNTT2	AT5G15090	ATVDAC3
AT1G15500	ATNTT2	AT5G57490	ATVDAC4
AT1G15690	ATAVP3	AT1G59870	ABCG36
AT1G15690	ATAVP3	AT3G57330	ACA11
AT1G15690	ATAVP3	AT4G30190	AHA2
AT1G15690	ATAVP3	AT5G57110	ACA8
AT1G16470	PAB1	AT1G56450	PBG1

AT1G16470	PAB1	AT4G31300	PBA1
AT1G16470	PAB1	AT5G42790	ARS5
AT1G16700		AT1G47260	APFI
AT1G16700		AT1G76030	
AT1G16700		AT1G79010	
AT1G16700		AT2G33040	ATP3
AT1G16700		AT3G01290	AtHIR2
AT1G16700		AT3G02090	MPPBETA
AT1G16700		AT3G27280	ATPHB4
AT1G16700		AT3G48680	AtCAL2
AT1G16700		AT3G57530	ATCPK32
AT1G16700		AT4G02580	
AT1G16700		AT4G16450	
AT1G16700		AT5G08060	
AT1G16700		AT5G08530	CI51
AT1G16700		AT5G11770	
AT1G16700		AT5G52840	
AT1G16700		ATMG00070	NAD9
AT1G18260	EBS5	AT1G75220	AtERDL6
AT1G18260	EBS5	AT2G01470	ATSEC12
AT1G18260	EBS5	AT2G40890	CYP98A3
AT1G18260	EBS5	AT3G13772	AtTMN7
AT1G18260	EBS5	AT4G29130	ATHXK1
AT1G18260	EBS5	AT4G35230	BSK1
AT1G18260	EBS5	AT5G07910	
AT1G18260	EBS5	AT5G18480	PGSIP6
AT1G18260	EBS5	AT5G27540	emb2473
AT1G18260	EBS5	AT5G52240	AtMAPR5
AT1G18540		AT3G18740	RLK902
AT1G19110		AT1G30360	ERD4
AT1G19110		AT1G55160	
AT1G19360	RRA3	AT5G23630	MIA
AT1G19580	GAMMA CA1	AT5G66510	GAMMA CA3
AT1G20010	TUB5	AT3G56150	ATEIF3C-1
AT1G20050	HYD1	AT5G16590	LRR1
AT1G20330	CVP1	AT2G30490	ATC4H
AT1G20330	CVP1	AT2G40890	CYP98A3
AT1G20330	CVP1	AT4G29130	ATHXK1
AT1G20330	CVP1	AT5G40510	
AT1G20575	DPMS1	AT3G13772	AtTMN7
AT1G20575	DPMS1	AT3G26210	CYP71B23
AT1G20575	DPMS1	AT5G20660	
AT1G22410		AT1G33140	PGY2
AT1G22410		AT3G63150	ATCBG
AT1G22410		AT4G26010	
AT1G22530	PATL2	AT1G72150	PATL1
AT1G25490	ATB BETA BETA	AT5G24710	
AT1G26850		AT1G30360	ERD4
AT1G26850		AT1G77590	LACS9
AT1G26850		AT2G18960	AHA1
AT1G26850		AT2G20360	
AT1G26850		AT2G37050	

AT1G26850		AT3G08580	AAC1
AT1G26850		AT3G16480	MPPalpha
AT1G26850		AT3G27240	
AT1G26850		AT3G57330	ACA11
AT1G26850		AT4G38350	
AT1G26850		AT5G04930	ALA1
AT1G26850		AT5G16590	LRR1
AT1G26850		AT5G19760	
AT1G26850		AT5G40810	
AT1G26850		AT5G49760	
AT1G26880		AT1G69620	RPL34
AT1G27190		AT3G28450	
AT1G27390	TOM20-2	AT1G72170	
AT1G27390	TOM20-2	AT3G07568	
AT1G27390	TOM20-2	AT5G53650	
AT1G27400		AT3G04400	emb2171
AT1G27400		AT1G57860	
AT1G27400		AT1G43170	ARP1
AT1G27400		AT1G66580	RPL10C
AT1G27400		AT3G24830	
AT1G27770	ACA1	AT2G04350	LACS8
AT1G27770	ACA1	ATMG00220	COB
AT1G27770	ACA1	AT2G17720	P4H5
AT1G27770	ACA1	AT2G25310	
AT1G27770	ACA1	AT2G47000	ABCB4
AT1G27770	ACA1	AT3G54110	ATPUMP1
AT1G27770	ACA1	AT4G00630	ATKEA2
AT1G27770	ACA1	AT4G21150	HAP6
AT1G27770	ACA1	AT5G11560	
AT1G27770	ACA1	AT5G13490	AAC2
AT1G27770	ACA1	AT5G15090	ATVDAC3
AT1G27770	ACA1	AT5G57490	ATVDAC4
AT1G27770	ACA1	AT5G66680	DGL1
AT1G27770	ACA1	AT5G67500	ATVDAC2
AT1G27980	ATDPL1	AT1G76400	
AT1G27980	ATDPL1	AT2G21160	
AT1G27980	ATDPL1	AT5G50460	
AT1G27980	ATDPL1	AT4G29130	ATHXK1
AT1G27980	ATDPL1	AT5G52240	AtMAPR5
AT1G28290	AGP31	AT1G66580	RPL10C
AT1G29470		AT5G14950	ATGMII
AT1G30360	ERD4	AT1G36050	
AT1G30360	ERD4	AT1G55160	
AT1G30360	ERD4	AT1G77590	LACS9
AT1G30360	ERD4	AT2G04350	LACS8
AT1G30360	ERD4	AT2G17720	P4H5
AT1G30360	ERD4	AT2G18960	AHA1
AT1G30360	ERD4	AT2G37050	
AT1G30360	ERD4	AT2G47000	ABCB4
AT1G30360	ERD4	AT3G13870	GOM8
AT1G30360	ERD4	AT3G16480	MPPalpha
AT1G30360	ERD4	AT3G27240	

AT1G30360	ERD4	AT3G54110	ATPUMP1
AT1G30360	ERD4	AT3G57330	ACA11
AT1G30360	ERD4	AT3G62700	ABCC14
AT1G30360	ERD4	AT4G29900	ACA10
AT1G30360	ERD4	AT4G38350	
AT1G30360	ERD4	AT5G16590	LRR1
AT1G30360	ERD4	AT5G19760	
AT1G30360	ERD4	AT5G40810	
AT1G30360	ERD4	AT5G57110	ACA8
AT1G32400	TOM2A	AT1G59870	ABCG36
AT1G33140	PGY2	AT4G26010	
AT1G33140	PGY2	AT5G42080	ADL1
AT1G33230		AT1G56110	NOP56
AT1G34130	STT3B	AT3G20920	
AT1G34430	EMB3003	AT3G25860	LTA2
AT1G35620	ATPDI8	AT2G17720	P4H5
AT1G35620	ATPDI8	AT2G21160	
AT1G35620	ATPDI8	AT2G45060	
AT1G35620	ATPDI8	AT2G47000	ABCB4
AT1G35620	ATPDI8	AT3G14680	CYP72A14
AT1G35620	ATPDI8	AT3G54110	ATPUMP1
AT1G35620	ATPDI8	AT3G60600	(AT)VAP
AT1G35620	ATPDI8	AT4G24520	AR1
AT1G35620	ATPDI8	AT4G29130	ATHXK1
AT1G35620	ATPDI8	AT5G15090	ATVDAC3
AT1G35620	ATPDI8	AT5G35460	
AT1G35620	ATPDI8	AT5G40510	
AT1G35620	ATPDI8	AT5G42570	
AT1G35620	ATPDI8	AT5G56730	
AT1G35620	ATPDI8	AT5G57490	ATVDAC4
AT1G36050		AT2G04350	LACS8
AT1G36050		AT2G18960	AHA1
AT1G36050		AT3G47833	SDH7
AT1G36050		AT3G54110	ATPUMP1
AT1G36050		AT3G62700	ABCC14
AT1G36050		AT4G12650	
AT1G36050		AT4G29900	ACA10
AT1G36050		AT5G11560	
AT1G36050		AT5G13490	AAC2
AT1G36050		AT5G19760	
AT1G43170	ARP1	AT3G04400	emb2171
AT1G43170	ARP1	AT2G34480	
AT1G43170	ARP1	AT3G04840	
AT1G47260	APFI	AT1G51980	
AT1G47260	APFI	AT1G63500	
AT1G47260	APFI	AT1G67350	
AT1G47260	APFI	AT1G79010	
AT1G47260	APFI	AT2G07698	
AT1G47260	APFI	AT2G18730	ATDGK3
AT1G47260	APFI	AT2G33040	ATP3
AT1G47260	APFI	AT2G36850	ATGSL08
AT1G47260	APFI	AT3G01290	AtHIR2

AT1G47260	APFI	AT3G02090	MPPBETA
AT1G47260	APFI	AT3G03100	
AT1G47260	APFI	AT3G07160	ATGSL10
AT1G47260	APFI	AT3G08580	AAC1
AT1G47260	APFI	AT3G42050	
AT1G47260	APFI	AT3G48680	AtCAL2
AT1G47260	APFI	AT3G51550	FER
AT1G47260	APFI	AT3G53480	ABCG37
AT1G47260	APFI	AT4G16450	
AT1G47260	APFI	AT4G32470	
AT1G47260	APFI	AT5G08060	
AT1G47260	APFI	AT5G08530	CI51
AT1G47260	APFI	AT5G11770	
AT1G47260	APFI	AT5G13450	ATP5
AT1G47260	APFI	AT5G18800	
AT1G47260	APFI	AT5G37510	CI76
AT1G47260	APFI	AT5G51570	
AT1G47260	APFI	AT5G52840	
AT1G47260	APFI	AT5G66510	GAMMA CA3
AT1G47260	APFI	ATMG00070	NAD9
AT1G49570		AT5G19780	TUA5
AT1G51630		AT1G53000	AtCKS
AT1G51800	IOS1	AT3G11730	ATFP8
AT1G51980		AT1G63500	
AT1G51980		AT1G67350	
AT1G51980		AT1G79010	
AT1G51980		AT2G07698	
AT1G51980		AT2G18730	ATDGK3
AT1G51980		AT2G18960	AHA1
AT1G51980		AT2G20360	
AT1G51980		AT2G33040	ATP3
AT1G51980		AT3G01290	AtHIR2
AT1G51980		AT3G02090	MPPBETA
AT1G51980		AT3G03100	
AT1G51980		AT3G08580	AAC1
AT1G51980		AT3G27240	
AT1G51980		AT3G28710	
AT1G51980		AT3G42050	
AT1G51980		AT3G48680	AtCAL2
AT1G51980		AT3G57330	ACA11
AT1G51980		AT4G16450	
AT1G51980		AT4G20150	
AT1G51980		AT4G29900	ACA10
AT1G51980		AT4G32470	
AT1G51980		AT5G08530	CI51
AT1G51980		AT5G11770	
AT1G51980		AT5G13450	ATP5
AT1G51980		AT5G18800	
AT1G51980		AT5G19760	
AT1G51980		AT5G37510	CI76
AT1G51980		AT5G40810	
AT1G51980		AT5G49760	

AT1G51980		AT5G51570	
AT1G51980		AT5G52840	
AT1G51980		AT5G57110	ACA8
AT1G51980		AT5G62740	ATHIR1
AT1G51980		AT5G66510	GAMMA CA3
AT1G51980		AT5G67590	FRO1
AT1G51980		ATMG00070	NAD9
AT1G52200		AT4G35860	ATGB2
AT1G52780		AT4G20110	BP80-3;1
AT1G52780		AT5G04930	ALA1
AT1G52780		AT5G49760	
AT1G53000	AtCKS	AT1G55160	
AT1G53000	AtCKS	AT2G04350	LACS8
AT1G53000	AtCKS	ATMG00220	COB
AT1G53000	AtCKS	AT2G17720	P4H5
AT1G53000	AtCKS	AT2G19080	
AT1G53000	AtCKS	AT2G45060	
AT1G53000	AtCKS	AT2G45140	PVA12
AT1G53000	AtCKS	AT2G47000	ABCB4
AT1G53000	AtCKS	AT3G05230	
AT1G53000	AtCKS	AT3G14680	CYP72A14
AT1G53000	AtCKS	AT3G24300	AMT1;3
AT1G53000	AtCKS	AT3G47833	SDH7
AT1G53000	AtCKS	AT3G54110	ATPUMP1
AT1G53000	AtCKS	AT3G60600	(AT)VAP
AT1G53000	AtCKS	AT4G12650	
AT1G53000	AtCKS	AT4G14070	AAE15
AT1G53000	AtCKS	AT4G21150	HAP6
AT1G53000	AtCKS	AT4G24520	AR1
AT1G53000	AtCKS	AT4G38350	
AT1G53000	AtCKS	AT5G05520	
AT1G53000	AtCKS	AT5G15090	ATVDAC3
AT1G53000	AtCKS	AT5G19760	
AT1G53000	AtCKS	AT5G57490	ATVDAC4
AT1G53000	AtCKS	AT5G66680	DGL1
AT1G53750	RPT1A	AT1G57860	
AT1G53750	RPT1A	AT2G20140	RPT2b
AT1G53750	RPT1A	AT2G20580	ATRPN1A
AT1G53750	RPT1A	AT2G34480	
AT1G53750	RPT1A	AT5G45775	
AT1G53750	RPT1A	AT3G05530	ATS6A.2
AT1G53750	RPT1A	AT5G58290	RPT3
AT1G55160		AT2G17720	P4H5
AT1G55160		AT2G37050	
AT1G55160		AT2G47000	ABCB4
AT1G55160		AT3G13870	GOM8
AT1G55160		AT3G54110	ATPUMP1
AT1G55160		AT3G60330	AHA7
AT1G55160		AT4G01100	ADNT1
AT1G55160		AT4G12650	
AT1G55160		AT4G24330	
AT1G55160		AT4G35300	TMT2

AT1G55160		AT5G05520	
AT1G55850	ATCSLE1	AT2G01470	ATSEC12
AT1G55850	ATCSLE1	AT2G21160	
AT1G55850	ATCSLE1	AT4G26410	
AT1G55850	ATCSLE1	AT5G09270	
AT1G55850	ATCSLE1	AT5G39410	
AT1G55850	ATCSLE1	AT5G43970	ATTOM22-V
AT1G55850	ATCSLE1	AT5G52240	AtMAPR5
AT1G56110	NOP56	AT5G23630	MIA
AT1G56110	NOP56	AT5G55610	
AT1G56140		AT5G04930	ALA1
AT1G56140		AT5G49760	
AT1G56330	ATSAR1	AT2G19860	ATHXK2
AT1G56450	PBG1	AT2G05840	PAA2
AT1G56450	PBG1	AT2G28000	CH-CPN60A
AT1G56450	PBG1	AT2G33210	HSP60-2
AT1G56450	PBG1	AT3G23990	HSP60
AT1G56450	PBG1	AT3G27430	PBB1
AT1G56450	PBG1	AT3G60820	PBF1
AT1G56450	PBG1	AT5G42790	ARS5
AT1G56450	PBG1	AT5G56500	Cpn60beta3
AT1G57860		AT2G34480	
AT1G57860		AT5G45775	
AT1G57860		AT4G27090	
AT1G57860		AT5G58290	RPT3
AT1G57860		AT5G64260	EXL2
AT1G59820	ALA3	AT5G13450	ATP5
AT1G59870	ABCG36	AT1G75680	AtGH9B7
AT1G59870	ABCG36	AT2G36380	ABCG34
AT1G59870	ABCG36	AT3G02880	
AT1G59870	ABCG36	AT3G08510	ATPLC2
AT1G59870	ABCG36	AT3G21630	CERK1
AT1G59870	ABCG36	AT5G14040	PHT3;1
AT1G61250	SC3	AT3G07950	
AT1G61250	SC3	AT4G28770	
AT1G61790		AT2G04350	LACS8
AT1G61790		ATMG00220	COB
AT1G61790		AT2G17720	P4H5
AT1G61790		AT2G18960	AHA1
AT1G61790		AT2G45140	PVA12
AT1G61790		AT3G13870	GOM8
AT1G61790		AT3G14610	CYP72A7
AT1G61790		AT4G21150	HAP6
AT1G61790		AT4G29900	ACA10
AT1G61790		AT5G15090	ATVDAC3
AT1G61790		AT5G19760	
AT1G61790		AT5G66680	DGL1
AT1G63500		AT1G79010	
AT1G63500		AT2G07698	
AT1G63500		AT2G20360	
AT1G63500		AT3G08580	AAC1
AT1G63500		AT3G27240	

AT1G63500		AT3G48680	AtCAL2
AT1G63500		AT3G57330	ACA11
AT1G63500		AT4G30190	AHA2
AT1G63500		AT4G32470	
AT1G63500		AT5G08530	CI51
AT1G63500		AT5G13450	ATP5
AT1G63500		AT5G37510	CI76
AT1G63500		AT5G52840	
AT1G63500		AT5G66510	GAMMA CA3
AT1G63500		AT5G67590	FRO1
AT1G63500		ATMG00070	NAD9
AT1G64090	RTNLB3	AT4G23630	BTI1
AT1G64090	RTNLB3	AT4G35790	ATPLDDELTA
AT1G64790	ILA	AT4G39850	ABCD1
AT1G65270		AT5G05370	
AT1G65270		AT4G12590	
AT1G65270		AT5G11560	
AT1G65270		AT5G13490	AAC2
AT1G65270		AT5G40770	ATPHB3
AT1G65270		AT5G47890	
AT1G65270		AT5G48000	CYP708 A2
AT1G65540		AT3G28860	ABCB19
AT1G65985		AT2G21580	
AT1G66580	RPL10C	AT3G04840	
AT1G67350		AT1G79010	
AT1G67350		AT2G07698	
AT1G67350		ATMG00480	ATP8
AT1G67350		AT2G20360	
AT1G67350		AT3G03100	
AT1G67350		AT3G07160	ATGSL10
AT1G67350		AT3G08580	AAC1
AT1G67350		AT3G18480	AtCASP
AT1G67350		AT3G27240	
AT1G67350		AT3G48680	AtCAL2
AT1G67350		AT4G16450	
AT1G67350		AT4G20150	
AT1G67350		AT4G32470	
AT1G67350		AT5G08530	CI51
AT1G67350		AT5G13450	ATP5
AT1G67350		AT5G37510	CI76
AT1G67350		AT5G52840	
AT1G67350		AT5G66510	GAMMA CA3
AT1G67350		ATMG00070	NAD9
AT1G67730	ATKCR1	AT5G01500	TAAC
AT1G68070		AT5G13490	AAC2
AT1G69460		AT5G01500	TAAC
AT1G69620	RPL34	AT3G28900	
AT1G69620	RPL34	AT3G53740	
AT1G69620	RPL34	AT3G55750	
AT1G70690	HWI1	AT1G75680	AtGH9B7
AT1G70690	HWI1	AT5G07910	
AT1G72150	PATL1	AT4G37070	AtPLAIVA

AT1G72170		AT3G07568	
AT1G72170		AT5G21990	OEP61
AT1G74020	SS2	AT5G42150	
AT1G74020	SS2	AT5G58070	ATTIL
AT1G75220	AtERDL6	AT3G28450	
AT1G75220	AtERDL6	AT4G35230	BSK1
AT1G75220	AtERDL6	AT5G07910	
AT1G75220	AtERDL6	AT5G18480	PGSIP6
AT1G76030		AT4G38510	
AT1G76400		AT2G01720	
AT1G76400		AT2G30490	ATC4H
AT1G76400		AT2G45060	
AT1G76400		AT3G01280	ATVDAC1
AT1G76400		AT3G14680	CYP72A14
AT1G76400		AT3G20940	CYP705A30
AT1G76400		AT3G26330	CYP71B37
AT1G76400		AT3G47833	SDH7
AT1G76400		AT3G60600	(AT)VAP
AT1G76400		AT3G66658	ALDH22A1
AT1G76400		AT4G14070	AAE15
AT1G76400		AT4G24520	AR1
AT1G76400		AT4G31500	ATR4
AT1G76400		AT4G35230	BSK1
AT1G76400		AT4G37640	ACA2
AT1G76400		AT4G38350	
AT1G76400		AT5G27540	emb2473
AT1G76400		AT5G40510	
AT1G76400		AT5G42590	CYP71A16
AT1G79010		AT2G07698	
AT1G79010		AT2G18730	ATDGK3
AT1G79010		AT2G33040	ATP3
AT1G79010		AT3G01290	AtHIR2
AT1G79010		AT3G02090	MPPBETA
AT1G79010		AT3G03100	
AT1G79010		AT3G08580	AAC1
AT1G79010		AT3G42050	
AT1G79010		AT3G48680	AtCAL2
AT1G79010		AT3G53480	ABCG37
AT1G79010		AT4G16450	
AT1G79010		AT4G20150	
AT1G79010		AT4G29900	ACA10
AT1G79010		AT4G32470	
AT1G79010		AT5G08530	CI51
AT1G79010		AT5G11770	
AT1G79010		AT5G13450	ATP5
AT1G79010		AT5G37510	CI76
AT1G79010		AT5G51570	
AT1G79010		AT5G52840	
AT1G79010		AT5G66510	GAMMA CA3
AT1G79010		ATMG00070	NAD9
AT2G01470	ATSEC12	AT2G24180	CYP71B6
AT2G01470	ATSEC12	AT2G30490	ATC4H

AT2G01470	ATSEC12	AT2G40890	CYP98A3
AT2G01470	ATSEC12	AT3G05970	ATLACS6
AT2G01470	ATSEC12	AT3G13772	AtTMN7
AT2G01470	ATSEC12	AT3G27570	
AT2G01470	ATSEC12	AT3G28450	
AT2G01470	ATSEC12	AT3G52190	AtPHF1
AT2G01470	ATSEC12	AT4G01320	ATSTE24
AT2G01470	ATSEC12	AT4G26410	
AT2G01470	ATSEC12	AT5G12470	
AT2G01470	ATSEC12	AT5G39410	
AT2G01690		AT3G02080	
AT2G01720		AT3G60600	(AT)VAP
AT2G01720		AT4G31500	ATR4
AT2G03510		AT2G43950	ATOEP37
AT2G03510		AT3G18410	
AT2G03510		AT3G62360	
AT2G03510		AT3G62830	ATUXS2
AT2G03510		AT4G17140	
AT2G03510		AT4G28510	ATPHB1
AT2G03510		AT5G05000	ATTOC34
AT2G03510		AT5G23630	MIA
AT2G03510		AT5G40480	EMB3012
AT2G04350	LACS8	ATMG00220	COB
AT2G04350	LACS8	AT2G17720	P4H5
AT2G04350	LACS8	AT2G18960	AHA1
AT2G04350	LACS8	AT2G47000	ABCB4
AT2G04350	LACS8	AT3G14610	CYP72A7
AT2G04350	LACS8	AT3G17910	EMB3121
AT2G04350	LACS8	AT3G24300	AMT1;3
AT2G04350	LACS8	AT3G47833	SDH7
AT2G04350	LACS8	AT3G54110	ATPUMP1
AT2G04350	LACS8	AT3G60600	(AT)VAP
AT2G04350	LACS8	AT3G62700	ABCC14
AT2G04350	LACS8	AT4G21150	HAP6
AT2G04350	LACS8	AT4G24520	AR1
AT2G04350	LACS8	AT4G37640	ACA2
AT2G04350	LACS8	AT5G10780	
AT2G04350	LACS8	AT5G11560	
AT2G04350	LACS8	AT5G13490	AAC2
AT2G04350	LACS8	AT5G14950	ATGMII
AT2G04350	LACS8	AT5G15090	ATVDAC3
AT2G04350	LACS8	AT5G19760	
AT2G04350	LACS8	AT5G66680	DGL1
AT2G04350	LACS8	AT5G67500	ATVDAC2
AT2G05840	PAA2	AT2G28000	CH-CPN60A
AT2G05840	PAA2	AT2G33210	HSP60-2
AT2G05840	PAA2	AT3G23990	HSP60
AT2G05840	PAA2	AT3G27430	PBB1
AT2G05840	PAA2	AT3G60820	PBF1
AT2G05840	PAA2	AT4G31300	PBA1
AT2G05840	PAA2	AT5G42790	ARS5
AT2G05840	PAA2	AT5G56500	Cpn60beta3

AT2G07698		AT2G33040	ATP3
AT2G07698		AT3G01290	AtHIR2
AT2G07698		AT3G02090	MPPBETA
AT2G07698		AT3G03100	
AT2G07698		AT3G08580	AAC1
AT2G07698		AT3G42050	
AT2G07698		AT3G48680	AtCAL2
AT2G07698		AT3G53480	ABCG37
AT2G07698		AT4G16450	
AT2G07698		AT4G32410	AtCESA1
AT2G07698		AT4G32470	
AT2G07698		AT5G08060	
AT2G07698		AT5G08530	CI51
AT2G07698		AT5G11770	
AT2G07698		AT5G13450	ATP5
AT2G07698		AT5G18800	
AT2G07698		AT5G37510	CI76
AT2G07698		AT5G51570	
AT2G07698		AT5G52840	
AT2G07698		AT5G66510	GAMMA CA3
AT2G07698		ATMG00070	NAD9
AT2G14740	ATVSR3	AT3G52850	ATELP
AT2G17440	PIRL5	AT5G51570	
AT2G17720	P4H5	AT2G18960	AHA1
AT2G17720	P4H5	AT2G45060	
AT2G17720	P4H5	AT2G45140	PVA12
AT2G17720	P4H5	AT2G47000	ABCB4
AT2G17720	P4H5	AT3G01280	ATVDAC1
AT2G17720	P4H5	AT3G05230	
AT2G17720	P4H5	AT3G13870	GOM8
AT2G17720	P4H5	AT3G14680	CYP72A14
AT2G17720	P4H5	AT3G47833	SDH7
AT2G17720	P4H5	AT3G54110	ATPUMP1
AT2G17720	P4H5	AT3G60600	(AT)VAP
AT2G17720	P4H5	AT4G14070	AAE15
AT2G17720	P4H5	AT4G21150	HAP6
AT2G17720	P4H5	AT4G24520	AR1
AT2G17720	P4H5	AT4G29900	ACA10
AT2G17720	P4H5	AT4G38350	
AT2G17720	P4H5	AT5G15090	ATVDAC3
AT2G17720	P4H5	AT5G19760	
AT2G17720	P4H5	AT5G35460	
AT2G17720	P4H5	AT5G42570	
AT2G17720	P4H5	AT5G56730	
AT2G17720	P4H5	AT5G57490	ATVDAC4
AT2G17720	P4H5	AT5G61790	ATCNX1
AT2G17720	P4H5	AT5G66680	DGL1
AT2G17720	P4H5	AT5G67500	ATVDAC2
AT2G18730	ATDGK3	AT3G01290	AtHIR2
AT2G18730	ATDGK3	AT3G02090	MPPBETA
AT2G18730	ATDGK3	AT3G08580	AAC1
AT2G18730	ATDGK3	AT3G48680	AtCAL2

AT2G18730	ATDGK3	AT4G29900	ACA10
AT2G18730	ATDGK3	AT5G08530	CI51
AT2G18730	ATDGK3	AT5G11770	
AT2G18730	ATDGK3	AT5G51570	
AT2G18730	ATDGK3	AT5G52840	
AT2G18730	ATDGK3	AT5G66510	GAMMA CA3
AT2G18730	ATDGK3	ATMG00070	NAD9
AT2G18960	AHA1	ATMG00220	COB
AT2G18960	AHA1	AT2G20360	
AT2G18960	AHA1	AT2G21410	VHA-A2
AT2G18960	AHA1	AT2G25310	
AT2G18960	AHA1	AT2G47000	ABCB4
AT2G18960	AHA1	AT3G01290	AtHIR2
AT2G18960	AHA1	AT3G08580	AAC1
AT2G18960	AHA1	AT3G16480	MPPalpha
AT2G18960	AHA1	AT3G24300	AMT1;3
AT2G18960	AHA1	AT3G27240	
AT2G18960	AHA1	AT3G28710	
AT2G18960	AHA1	AT3G47833	SDH7
AT2G18960	AHA1	AT3G48680	AtCAL2
AT2G18960	AHA1	AT3G54110	ATPUMP1
AT2G18960	AHA1	AT3G57330	ACA11
AT2G18960	AHA1	AT3G62700	ABCC14
AT2G18960	AHA1	AT4G29900	ACA10
AT2G18960	AHA1	AT4G30190	AHA2
AT2G18960	AHA1	AT4G38350	
AT2G18960	AHA1	AT5G13490	AAC2
AT2G18960	AHA1	AT5G14950	ATGMII
AT2G18960	AHA1	AT5G19760	
AT2G18960	AHA1	AT5G40810	
AT2G18960	AHA1	AT5G51570	
AT2G18960	AHA1	AT5G57110	ACA8
AT2G18960	AHA1	ATMG00070	NAD9
AT2G19080		AT3G14680	CYP72A14
AT2G19080		AT4G14070	AAE15
AT2G19680		AT4G26210	
AT2G19680		AT4G29480	
AT2G19860	ATHXK2	AT2G21160	
AT2G19860	ATHXK2	AT2G46650	ATCB5-C
AT2G19860	ATHXK2	AT3G13772	AtTMN7
AT2G19860	ATHXK2	AT4G00900	ATECA2
AT2G19860	ATHXK2	AT4G29130	ATHXK1
AT2G19860	ATHXK2	AT5G27540	emb2473
AT2G19860	ATHXK2	ATMG00160	COX2
AT2G20140	RPT2b	AT2G20580	ATRPN1A
AT2G20140	RPT2b	AT2G34480	
AT2G20140	RPT2b	AT3G05530	ATS6A.2
AT2G20140	RPT2b	AT5G58290	RPT3
AT2G20360		AT2G33040	ATP3
AT2G20360		AT3G01290	AtHIR2
AT2G20360		AT3G08580	AAC1
AT2G20360		AT3G16480	MPPalpha

AT2G20360		AT3G27240	
AT2G20360		AT3G28710	
AT2G20360		AT3G48680	AtCAL2
AT2G20360		AT3G57330	ACA11
AT2G20360		AT4G29900	ACA10
AT2G20360		AT5G08530	CI51
AT2G20360		AT5G16590	LRR1
AT2G20360		AT5G19760	
AT2G20360		AT5G37510	CI76
AT2G20360		AT5G40810	
AT2G20360		AT5G49760	
AT2G20360		AT5G51570	
AT2G20360		AT5G57110	ACA8
AT2G20360		AT5G62740	ATHIR1
AT2G20360		AT5G64030	
AT2G20360		AT5G66510	GAMMA CA3
AT2G20360		AT5G67590	FRO1
AT2G20360		ATMG00070	NAD9
AT2G20530	ATPHB6	AT3G02090	MPPBETA
AT2G20530	ATPHB6	AT3G27280	ATPHB4
AT2G20530	ATPHB6	AT5G11770	
AT2G20530	ATPHB6	AT5G40770	ATPHB3
AT2G20530	ATPHB6	ATMG00270	NAD6
AT2G20580	ATRPN1A	AT2G34480	
AT2G20580	ATRPN1A	AT3G05530	ATS6A.2
AT2G20580	ATRPN1A	AT5G58290	RPT3
AT2G20760		AT3G08530	
AT2G20760		AT4G39200	
AT2G20990	ATSYTA	AT2G33040	ATP3
AT2G20990	ATSYTA	AT2G38670	PECT1
AT2G20990	ATSYTA	AT3G02090	MPPBETA
AT2G20990	ATSYTA	AT5G05370	
AT2G20990	ATSYTA	AT3G44330	
AT2G20990	ATSYTA	AT3G57650	LPAT2
AT2G20990	ATSYTA	AT4G12590	
AT2G20990	ATSYTA	AT4G29900	ACA10
AT2G21160		AT2G30490	ATC4H
AT2G21160		AT2G40890	CYP98A3
AT2G21160		AT3G13772	AtTMN7
AT2G21160		AT3G14680	CYP72A14
AT2G21160		AT3G61070	PEX11E
AT2G21160		AT4G29130	ATHXK1
AT2G21160		AT5G12470	
AT2G21160		AT5G14030	
AT2G21160		AT5G20660	
AT2G21160		AT5G42570	
AT2G21390		AT4G31490	
AT2G21410	VHA-A2	AT3G01290	AtHIR2
AT2G21410	VHA-A2	AT3G28710	
AT2G21410	VHA-A2	AT3G57330	ACA11
AT2G21410	VHA-A2	AT3G62700	ABCC14
AT2G21410	VHA-A2	AT4G30190	AHA2

AT2G21410	VHA-A2	AT5G40810	
AT2G21410	VHA-A2	AT5G51570	
AT2G21410	VHA-A2	AT5G57110	ACA8
AT2G21410	VHA-A2	ATMG00070	NAD9
AT2G23770		AT4G16450	
AT2G23810	TET8	AT2G39420	
AT2G23810	TET8	AT5G06320	NHL3
AT2G23810	TET8	AT5G54440	AtTRS130
AT2G24180	CYP71B6	AT4G01320	ATSTE24
AT2G24180	CYP71B6	AT5G23300	PYRD
AT2G24180	CYP71B6	AT5G39410	
AT2G25160	CYP82F1	AT2G35720	OWL1
AT2G25160	CYP82F1	AT5G05000	ATTOC34
AT2G25310		AT4G38920	ATVHA-C3
AT2G25310		AT3G03060	
AT2G25310		AT5G05370	
AT2G25310		AT5G11560	
AT2G25310		AT5G13490	AAC2
AT2G25310		AT5G14950	ATGMII
AT2G25310		AT5G50650	
AT2G25310		AT5G55940	emb2731
AT2G25310		AT5G66680	DGL1
AT2G25310		AT5G67500	ATVDAC2
AT2G25430		AT2G40060	CLC2
AT2G25430		AT4G32285	
AT2G26140	ftsh4	AT3G03100	
AT2G26140	ftsh4	AT3G07160	ATGSL10
AT2G26140	ftsh4	AT3G51550	FER
AT2G26140	ftsh4	AT4G02580	
AT2G26140	ftsh4	AT4G16450	
AT2G26140	ftsh4	AT4G33430	ATBAK1
AT2G26140	ftsh4	AT5G08060	
AT2G26140	ftsh4	AT5G11770	
AT2G26140	ftsh4	AT5G52840	
AT2G26890	GRV2	AT3G11730	ATFP8
AT2G28000	CH-CPN60A	AT2G33210	HSP60-2
AT2G28000	CH-CPN60A	AT3G23990	HSP60
AT2G28000	CH-CPN60A	AT3G60820	PBF1
AT2G28000	CH-CPN60A	AT5G42790	ARS5
AT2G28000	CH-CPN60A	AT5G56500	Cpn60beta3
AT2G28430		AT3G07568	
AT2G28520	VHA-A1	AT5G08530	CI51
AT2G28520	VHA-A1	AT5G37510	CI76
AT2G28520	VHA-A1	ATMG00070	NAD9
AT2G30490	ATC4H	AT2G40890	CYP98A3
AT2G30490	ATC4H	AT3G13772	AtTMN7
AT2G30490	ATC4H	AT3G20940	CYP705A30
AT2G30490	ATC4H	AT3G26210	CYP71B23
AT2G30490	ATC4H	AT3G52190	AtPHF1
AT2G30490	ATC4H	AT4G29130	ATHXK1
AT2G30490	ATC4H	AT4G31500	ATR4
AT2G30490	ATC4H	AT5G20660	

AT2G30490	ATC4H	AT5G42570	
AT2G31610		AT3G02560	
AT2G31610		AT3G14840	
AT2G31610		AT5G45750	AtRABA1c
AT2G32720	ATCB5-B	AT4G39690	
AT2G33040	ATP3	AT2G36850	ATGSL08
AT2G33040	ATP3	AT3G02090	MPPBETA
AT2G33040	ATP3	AT3G08580	AAC1
AT2G33040	ATP3	AT3G28710	
AT2G33040	ATP3	AT3G42050	
AT2G33040	ATP3	AT3G48680	AtCAL2
AT2G33040	ATP3	AT4G32470	
AT2G33040	ATP3	AT5G08530	CI51
AT2G33040	ATP3	AT5G11560	
AT2G33040	ATP3	AT5G40770	ATPHB3
AT2G33040	ATP3	AT5G62740	ATHIR1
AT2G33040	ATP3	AT5G66510	GAMMA CA3
AT2G33040	ATP3	ATMG00070	NAD9
AT2G33120	ATVAMP722	AT3G52850	ATELP
AT2G33120	ATVAMP722	AT5G22360	ATVAMP714
AT2G33210	HSP60-2	AT3G23990	HSP60
AT2G33210	HSP60-2	AT3G60820	PBF1
AT2G33210	HSP60-2	AT5G56500	Cpn60beta3
AT2G33630		AT5G05000	ATTOC34
AT2G33630		AT5G47420	
AT2G34250		AT4G21150	HAP6
AT2G34250		AT5G11560	
AT2G34250		AT5G66680	DGL1
AT2G34480		AT3G04400	emb2171
AT2G34480		AT5G45775	
AT2G34480		AT3G04840	
AT2G34480		AT4G27090	
AT2G34660	ABCC2	AT3G27280	ATPHB4
AT2G34660	ABCC2	AT3G57650	LPAT2
AT2G36380	ABCG34	AT3G08510	ATPLC2
AT2G36380	ABCG34	AT4G36750	
AT2G36380	ABCG34	AT5G14040	PHT3;1
AT2G36850	ATGSL08	AT3G42050	
AT2G36850	ATGSL08	AT3G51550	FER
AT2G36850	ATGSL08	AT4G16450	
AT2G36850	ATGSL08	AT5G08060	
AT2G36850	ATGSL08	AT5G08530	CI51
AT2G36850	ATGSL08	AT5G11770	
AT2G36850	ATGSL08	AT5G52840	
AT2G36850	ATGSL08	AT5G66510	GAMMA CA3
AT2G37050		AT2G47000	ABCB4
AT2G37050		AT3G54110	ATPUMP1
AT2G37050		AT3G60330	AHA7
AT2G37050		AT4G12650	
AT2G37050		AT4G35300	TMT2
AT2G37050		AT4G38350	
AT2G37050		AT5G16590	LRR1

AT2G37050		AT5G19760	
AT2G38670	PECT1	AT4G03560	ATCCH1
AT2G38670	PECT1	AT5G11560	
AT2G39420		AT5G06320	NHL3
AT2G40060	CLC2	AT3G11130	
AT2G40890	CYP98A3	AT3G07570	
AT2G40890	CYP98A3	AT3G13772	AtTMN7
AT2G40890	CYP98A3	AT3G26210	CYP71B23
AT2G40890	CYP98A3	AT3G28450	
AT2G40890	CYP98A3	AT3G52190	AtPHF1
AT2G40890	CYP98A3	AT4G00900	ATECA2
AT2G40890	CYP98A3	AT4G01320	ATSTE24
AT2G40890	CYP98A3	AT4G29130	ATHXK1
AT2G40890	CYP98A3	AT4G29330	DER1
AT2G40890	CYP98A3	AT4G31500	ATR4
AT2G40890	CYP98A3	AT4G35230	BSK1
AT2G40890	CYP98A3	AT5G07910	
AT2G40890	CYP98A3	AT5G18480	PGSIP6
AT2G40890	CYP98A3	AT5G20660	
AT2G40890	CYP98A3	AT5G25100	
AT2G40890	CYP98A3	AT5G27540	emb2473
AT2G40890	CYP98A3	AT5G58270	ABCB25
AT2G40890	CYP98A3	ATMG00160	COX2
AT2G42210	ATOEP16-3	AT5G47570	
AT2G43950	ATOEP37	AT3G55360	ATTSC13
AT2G43950	ATOEP37	AT3G62360	
AT2G43950	ATOEP37	AT4G28510	ATPHB1
AT2G43950	ATOEP37	AT5G40480	EMB3012
AT2G43950	ATOEP37	AT5G42960	
AT2G44640		AT5G58270	ABCB25
AT2G45010		AT3G52400	ATSYP122
AT2G45060		AT2G47000	ABCB4
AT2G45060		AT3G01280	ATVDAC1
AT2G45060		AT3G14680	CYP72A14
AT2G45060		AT3G47833	SDH7
AT2G45060		AT3G54110	ATPUMP1
AT2G45060		AT3G60600	(AT)VAP
AT2G45060		AT4G24520	AR1
AT2G45060		AT5G15090	ATVDAC3
AT2G45060		AT5G40510	
AT2G45060		AT5G42570	
AT2G45060		AT5G57490	ATVDAC4
AT2G45140	PVA12	AT2G47000	ABCB4
AT2G45140	PVA12	AT3G14680	CYP72A14
AT2G45140	PVA12	AT3G47833	SDH7
AT2G45140	PVA12	AT3G54110	ATPUMP1
AT2G45140	PVA12	AT3G66658	ALDH22A1
AT2G45140	PVA12	AT4G14070	AAE15
AT2G45140	PVA12	AT4G21150	HAP6
AT2G45140	PVA12	AT4G24520	AR1
AT2G45140	PVA12	AT5G15090	ATVDAC3
AT2G45140	PVA12	AT5G57490	ATVDAC4

AT2G45510	CYP704A2	AT3G48410	
AT2G46650	ATCB5-C	AT4G00900	ATECA2
AT2G46650	ATCB5-C	ATMG00160	COX2
AT2G47000	ABCB4	AT3G13870	GOM8
AT2G47000	ABCB4	AT3G47833	SDH7
AT2G47000	ABCB4	AT3G54110	ATPUMP1
AT2G47000	ABCB4	AT3G60330	AHA7
AT2G47000	ABCB4	AT4G12650	
AT2G47000	ABCB4	AT4G21150	HAP6
AT2G47000	ABCB4	AT4G24520	AR1
AT2G47000	ABCB4	AT4G35300	TMT2
AT2G47000	ABCB4	AT4G37640	ACA2
AT2G47000	ABCB4	AT4G38350	
AT2G47000	ABCB4	AT5G15090	ATVDAC3
AT2G47000	ABCB4	AT5G19760	
AT2G47000	ABCB4	AT5G42570	
AT2G47000	ABCB4	AT5G61790	ATCNX1
AT2G47000	ABCB4	AT5G66680	DGL1
AT2G47000	ABCB4	AT5G67500	ATVDAC2
AT3G01280	ATVDAC1	AT3G14680	CYP72A14
AT3G01280	ATVDAC1	AT3G60600	(AT)VAP
AT3G01280	ATVDAC1	AT4G21150	HAP6
AT3G01280	ATVDAC1	AT4G24520	AR1
AT3G01280	ATVDAC1	AT4G31500	ATR4
AT3G01280	ATVDAC1	AT4G37640	ACA2
AT3G01280	ATVDAC1	AT5G13490	AAC2
AT3G01280	ATVDAC1	AT5G15090	ATVDAC3
AT3G01280	ATVDAC1	AT5G42570	
AT3G01280	ATVDAC1	AT5G57490	ATVDAC4
AT3G01280	ATVDAC1	AT5G61790	ATCNX1
AT3G01280	ATVDAC1	AT5G66680	DGL1
AT3G01280	ATVDAC1	AT5G67500	ATVDAC2
AT3G01290	AtHIR2	AT3G02090	MPPBETA
AT3G01290	AtHIR2	AT3G08580	AAC1
AT3G01290	AtHIR2	AT3G27240	
AT3G01290	AtHIR2	AT3G28710	
AT3G01290	AtHIR2	AT3G42050	
AT3G01290	AtHIR2	AT3G48680	AtCAL2
AT3G01290	AtHIR2	AT3G57330	ACA11
AT3G01290	AtHIR2	AT3G62360	
AT3G01290	AtHIR2	AT4G16450	
AT3G01290	AtHIR2	AT4G27585	
AT3G01290	AtHIR2	AT4G30190	AHA2
AT3G01290	AtHIR2	AT4G32470	
AT3G01290	AtHIR2	AT4G39080	VHA-A3
AT3G01290	AtHIR2	AT5G08530	CI51
AT3G01290	AtHIR2	AT5G11770	
AT3G01290	AtHIR2	AT5G13490	AAC2
AT3G01290	AtHIR2	AT5G19760	
AT3G01290	AtHIR2	AT5G40770	ATPHB3
AT3G01290	AtHIR2	AT5G40810	
AT3G01290	AtHIR2	AT5G51570	

AT3G01290	AtHIR2	AT5G52840	
AT3G01290	AtHIR2	AT5G57110	ACA8
AT3G01290	AtHIR2	AT5G62740	ATHIR1
AT3G01290	AtHIR2	AT5G66510	GAMMA CA3
AT3G02090	MPPBETA	AT3G08580	AAC1
AT3G02090	MPPBETA	AT3G27280	ATPHB4
AT3G02090	MPPBETA	AT3G42050	
AT3G02090	MPPBETA	AT3G48680	AtCAL2
AT3G02090	MPPBETA	AT4G16450	
AT3G02090	MPPBETA	AT4G29900	ACA10
AT3G02090	MPPBETA	AT4G32470	
AT3G02090	MPPBETA	AT5G08530	CI51
AT3G02090	MPPBETA	AT5G11770	
AT3G02090	MPPBETA	AT5G40770	ATPHB3
AT3G02090	MPPBETA	AT5G51570	
AT3G02090	MPPBETA	AT5G52840	
AT3G02090	MPPBETA	AT5G66510	GAMMA CA3
AT3G02090	MPPBETA	ATMG00070	NAD9
AT3G02560		AT4G08850	
AT3G02880		AT3G08510	ATPLC2
AT3G02880		AT3G21630	CERK1
AT3G02880		AT5G14040	PHT3;1
AT3G03060		AT3G27280	ATPHB4
AT3G03060		AT4G28510	ATPHB1
AT3G03060		AT5G40770	ATPHB3
AT3G03100		AT3G07160	ATGSL10
AT3G03100		AT3G08580	AAC1
AT3G03100		AT3G27390	
AT3G03100		AT3G42050	
AT3G03100		AT3G48680	AtCAL2
AT3G03100		AT4G16450	
AT3G03100		AT4G20150	
AT3G03100		AT4G32470	
AT3G03100		AT5G08060	
AT3G03100		AT5G08530	CI51
AT3G03100		AT5G13450	ATP5
AT3G03100		AT5G37510	CI76
AT3G03100		AT5G52840	
AT3G03100		AT5G66510	GAMMA CA3
AT3G03100		ATMG00070	NAD9
AT3G03305		AT3G46740	MAR1
AT3G03305		AT5G40890	ATCLC-A
AT3G03960		AT3G11830	
AT3G03960		AT3G18190	
AT3G03960		AT3G20050	ATTCP-1
AT3G03960		AT5G26360	
AT3G04400	emb2171	AT3G04840	
AT3G04400	emb2171	AT4G27090	
AT3G05230		AT3G51830	ATG5
AT3G05230		AT4G12650	
AT3G05230		AT4G38350	
AT3G05280		AT4G30260	

AT3G05560		AT5G27770	
AT3G05970	ATLACS6	AT3G27570	
AT3G05970	ATLACS6	AT3G28450	
AT3G05970	ATLACS6	AT3G57010	
AT3G05970	ATLACS6	AT5G43970	ATTOM22-V
AT3G05970	ATLACS6	AT5G58270	ABCB25
AT3G07160	ATGSL10	AT3G48680	AtCAL2
AT3G07160	ATGSL10	AT3G51550	FER
AT3G07160	ATGSL10	AT3G53480	ABCG37
AT3G07160	ATGSL10	AT4G02580	
AT3G07160	ATGSL10	AT4G16450	
AT3G07160	ATGSL10	AT4G32470	
AT3G07160	ATGSL10	AT4G33430	ATBAK1
AT3G07160	ATGSL10	AT5G08060	
AT3G07160	ATGSL10	AT5G08530	CI51
AT3G07160	ATGSL10	AT5G37510	CI76
AT3G07160	ATGSL10	AT5G52840	
AT3G07160	ATGSL10	AT5G66510	GAMMA CA3
AT3G07568		AT5G40930	TOM20-4
AT3G07568		AT5G53650	
AT3G07570		AT3G28450	
AT3G07570		AT4G29130	ATHXK1
AT3G07570		AT5G18480	PGSIP6
AT3G07570		AT5G25100	
AT3G07950		AT4G28770	
AT3G08510	ATPLC2	AT3G57330	ACA11
AT3G08510	ATPLC2	AT4G30190	AHA2
AT3G08510	ATPLC2	AT5G49760	
AT3G08510	ATPLC2	AT5G57110	ACA8
AT3G08530		AT4G39200	
AT3G08580	AAC1	AT3G27240	
AT3G08580	AAC1	AT3G28710	
AT3G08580	AAC1	AT3G42050	
AT3G08580	AAC1	AT3G48680	AtCAL2
AT3G08580	AAC1	AT3G57330	ACA11
AT3G08580	AAC1	AT4G29900	ACA10
AT3G08580	AAC1	AT4G32470	
AT3G08580	AAC1	AT5G08530	CI51
AT3G08580	AAC1	AT5G11770	
AT3G08580	AAC1	AT5G13450	ATP5
AT3G08580	AAC1	AT5G18800	
AT3G08580	AAC1	AT5G19760	
AT3G08580	AAC1	AT5G37510	CI76
AT3G08580	AAC1	AT5G40810	
AT3G08580	AAC1	AT5G49760	
AT3G08580	AAC1	AT5G52840	
AT3G08580	AAC1	AT5G64030	
AT3G08580	AAC1	AT5G66510	GAMMA CA3
AT3G08580	AAC1	AT5G67590	FRO1
AT3G08580	AAC1	ATMG00070	NAD9
AT3G10950		AT5G45775	
AT3G11070		AT5G05520	

AT3G11070		AT5G15090	ATVDAC3
AT3G11130		AT4G31985	
AT3G11730	ATFP8	AT3G14840	
AT3G11830		AT3G18190	
AT3G11830		AT3G20050	ATTCP-1
AT3G11830		AT5G26360	
AT3G13772	AtTMN7	AT3G26210	CYP71B23
AT3G13772	AtTMN7	AT4G00900	ATECA2
AT3G13772	AtTMN7	AT4G01320	ATSTE24
AT3G13772	AtTMN7	AT4G29130	ATHXK1
AT3G13772	AtTMN7	AT4G35230	BSK1
AT3G13772	AtTMN7	AT5G08080	ATSYP132
AT3G13772	AtTMN7	AT5G18480	PGSIP6
AT3G13772	AtTMN7	AT5G20660	
AT3G13772	AtTMN7	AT5G25100	
AT3G13772	AtTMN7	AT5G52240	AtMAPR5
AT3G13772	AtTMN7	AT5G58270	ABCB25
AT3G13870	GOM8	AT3G54110	ATPUMP1
AT3G13870	GOM8	AT3G57330	ACA11
AT3G13870	GOM8	AT4G29900	ACA10
AT3G13870	GOM8	AT5G19760	
AT3G14610	CYP72A7	ATMG00220	COB
AT3G14610	CYP72A7	AT3G47833	SDH7
AT3G14610	CYP72A7	AT3G54110	ATPUMP1
AT3G14610	CYP72A7	AT4G21150	HAP6
AT3G14610	CYP72A7	AT4G37640	ACA2
AT3G14610	CYP72A7	AT5G10780	
AT3G14610	CYP72A7	AT5G11560	
AT3G14610	CYP72A7	AT5G13490	AAC2
AT3G14610	CYP72A7	AT5G66680	DGL1
AT3G14610	CYP72A7	AT5G67500	ATVDAC2
AT3G14680	CYP72A14	AT3G20940	CYP705A30
AT3G14680	CYP72A14	AT3G47833	SDH7
AT3G14680	CYP72A14	AT3G54110	ATPUMP1
AT3G14680	CYP72A14	AT3G60600	(AT)VAP
AT3G14680	CYP72A14	AT4G14070	AAE15
AT3G14680	CYP72A14	AT4G21150	HAP6
AT3G14680	CYP72A14	AT4G24520	AR1
AT3G14680	CYP72A14	AT4G29130	ATHXK1
AT3G14680	CYP72A14	AT4G31500	ATR4
AT3G14680	CYP72A14	AT5G15090	ATVDAC3
AT3G14680	CYP72A14	AT5G42570	
AT3G14680	CYP72A14	AT5G57490	ATVDAC4
AT3G14840		AT4G00710	BSK3
AT3G14840		AT4G00740	QUA3
AT3G14840		AT5G45750	AtRABA1c
AT3G16480	MPPalpha	AT3G27240	
AT3G16480	MPPalpha	AT3G57330	ACA11
AT3G16480	MPPalpha	AT4G29900	ACA10
AT3G16480	MPPalpha	AT5G16590	LRR1
AT3G16480	MPPalpha	AT5G19760	
AT3G16480	MPPalpha	AT5G40810	

AT3G16480	MPPalpha	AT5G49760	
AT3G16480	MPPalpha	AT5G64030	
AT3G16480	MPPalpha	AT5G67590	FRO1
AT3G18190		AT3G20050	ATTCP-1
AT3G18190		AT5G26360	
AT3G18410		AT3G27280	ATPHB4
AT3G18410		AT3G46740	MAR1
AT3G18410		AT3G62360	
AT3G18410		AT4G16450	
AT3G18410		AT4G17140	
AT3G18410		AT4G27585	
AT3G18410		AT4G28510	ATPHB1
AT3G18410		AT4G32470	
AT3G18410		AT5G11770	
AT3G18410		AT5G40480	EMB3012
AT3G18410		AT5G40770	ATPHB3
AT3G18820	ATRAB7B	AT4G39990	ATGB3
AT3G19340		AT3G27820	ATMDAR4
AT3G19930	ATSTP4	AT4G25640	ATDTX35
AT3G20050	ATTCP-1	AT5G26360	
AT3G20920		AT4G38240	CGL
AT3G20940	CYP705A30	AT3G60600	(AT)VAP
AT3G20940	CYP705A30	AT4G31500	ATR4
AT3G20940	CYP705A30	AT4G35230	BSK1
AT3G20940	CYP705A30	AT5G20660	
AT3G20940	CYP705A30	AT5G27540	emb2473
AT3G20940	CYP705A30	AT5G42570	
AT3G21630	CERK1	AT5G14040	PHT3;1
AT3G22110	PAC1	AT3G27430	PBB1
AT3G22110	PAC1	AT3G60820	PBF1
AT3G22110	PAC1	AT4G31300	PBA1
AT3G22110	PAC1	AT5G42790	ARS5
AT3G22110	PAC1	AT5G56500	Cpn60beta3
AT3G22370	AOX1A	AT3G57010	
AT3G22370	AOX1A	AT5G01500	TAAC
AT3G23990	HSP60	AT3G60820	PBF1
AT3G23990	HSP60	AT5G42790	ARS5
AT3G23990	HSP60	AT5G56500	Cpn60beta3
AT3G24300	AMT1;3	ATMG00220	COB
AT3G24300	AMT1;3	AT3G47833	SDH7
AT3G24300	AMT1;3	AT3G54110	ATPUMP1
AT3G24300	AMT1;3	AT4G21150	HAP6
AT3G24300	AMT1;3	AT5G19760	
AT3G25290		AT5G07910	
AT3G26210	CYP71B23	AT4G01320	ATSTE24
AT3G26210	CYP71B23	AT5G12470	
AT3G26210	CYP71B23	AT5G20660	
AT3G26210	CYP71B23	AT5G36220	CYP81D1
AT3G27240		AT3G28710	
AT3G27240		AT3G48680	AtCAL2
AT3G27240		AT3G57330	ACA11
AT3G27240		AT4G29900	ACA10

AT3G27240		AT5G08530	CI51
AT3G27240		AT5G14950	ATGMII
AT3G27240		AT5G19760	
AT3G27240		AT5G37510	CI76
AT3G27240		AT5G40810	
AT3G27240		AT5G49760	
AT3G27240		AT5G57110	ACA8
AT3G27240		AT5G62740	ATHIR1
AT3G27240		AT5G66510	GAMMA CA3
AT3G27240		AT5G67590	FRO1
AT3G27240		ATMG00070	NAD9
AT3G27280	ATPHB4	AT5G05370	
AT3G27280	ATPHB4	AT3G44330	
AT3G27280	ATPHB4	AT3G57650	LPAT2
AT3G27280	ATPHB4	AT4G27585	
AT3G27280	ATPHB4	AT4G28510	ATPHB1
AT3G27280	ATPHB4	AT4G39080	VHA-A3
AT3G27280	ATPHB4	AT5G11770	
AT3G27280	ATPHB4	AT5G25450	
AT3G27280	ATPHB4	AT5G40770	ATPHB3
AT3G27280	ATPHB4	AT5G46800	BOU
AT3G27325		AT5G53170	FTSH11
AT3G27430	PBB1	AT3G60820	PBF1
AT3G27430	PBB1	AT4G31300	PBA1
AT3G27430	PBB1	AT5G42790	ARS5
AT3G27430	PBB1	AT5G56500	Cpn60beta3
AT3G27570		AT3G57010	
AT3G28450		AT3G52190	AtPHF1
AT3G28450		AT5G18480	PGSIP6
AT3G28450		AT5G40780	LHT1
AT3G28450		ATMG00160	COX2
AT3G28710		AT3G48680	AtCAL2
AT3G28710		AT3G57330	ACA11
AT3G28710		AT4G29900	ACA10
AT3G28710		AT4G30190	AHA2
AT3G28710		AT5G08530	CI51
AT3G28710		AT5G13490	AAC2
AT3G28710		AT5G40810	
AT3G28710		AT5G46800	BOU
AT3G28710		AT5G62740	ATHIR1
AT3G28710		AT5G66510	GAMMA CA3
AT3G28710		ATMG00070	NAD9
AT3G28720		AT4G17140	
AT3G28720		AT5G40890	ATCLC-A
AT3G28860	ABCB19	AT4G35300	TMT2
AT3G42050		AT3G48680	AtCAL2
AT3G42050		AT4G16450	
AT3G42050		AT4G29900	ACA10
AT3G42050		AT4G32470	
AT3G42050		AT5G08530	CI51
AT3G42050		AT5G11770	
AT3G42050		AT5G37510	CI76

AT3G42050		AT5G51570	
AT3G42050		AT5G52840	
AT3G42050		AT5G66510	GAMMA CA3
AT3G42050		ATMG00070	NAD9
AT3G44330		AT4G12590	
AT3G44330		AT5G40770	ATPHB3
AT3G44330		AT5G46800	BOU
AT3G46280		AT5G14040	PHT3;1
AT3G46740	MAR1	AT3G55360	ATTSC13
AT3G46740	MAR1	AT3G62360	
AT3G46740	MAR1	AT4G17140	
AT3G46740	MAR1	AT4G27585	
AT3G46740	MAR1	AT5G05000	ATTOC34
AT3G46740	MAR1	AT5G23630	MIA
AT3G46740	MAR1	AT5G40480	EMB3012
AT3G46740	MAR1	AT5G40890	ATCLC-A
AT3G47833	SDH7	ATMG00220	COB
AT3G47833	SDH7	AT3G54110	ATPUMP1
AT3G47833	SDH7	AT3G60600	(AT)VAP
AT3G47833	SDH7	AT3G62700	ABCC14
AT3G47833	SDH7	AT4G12650	
AT3G47833	SDH7	AT4G21150	HAP6
AT3G47833	SDH7	AT4G24520	AR1
AT3G47833	SDH7	AT4G37640	ACA2
AT3G47833	SDH7	AT4G38350	
AT3G47833	SDH7	AT5G11560	
AT3G47833	SDH7	AT5G15090	ATVDAC3
AT3G47833	SDH7	AT5G19760	
AT3G47833	SDH7	AT5G40510	
AT3G47833	SDH7	AT5G56730	
AT3G47833	SDH7	AT5G57490	ATVDAC4
AT3G47833	SDH7	AT5G66680	DGL1
AT3G47833	SDH7	AT5G67500	ATVDAC2
AT3G48380		AT5G50650	
AT3G48380		AT5G51510	
AT3G48380		AT5G66680	DGL1
AT3G48410		AT5G17770	ATCBR
AT3G48570		AT5G58640	
AT3G48570		ATMG00160	COX2
AT3G48680	AtCAL2	AT3G51550	FER
AT3G48680	AtCAL2	AT3G53480	ABCG37
AT3G48680	AtCAL2	AT3G57330	ACA11
AT3G48680	AtCAL2	AT4G16450	
AT3G48680	AtCAL2	AT4G20150	
AT3G48680	AtCAL2	AT4G29900	ACA10
AT3G48680	AtCAL2	AT4G32470	
AT3G48680	AtCAL2	AT5G08530	CI51
AT3G48680	AtCAL2	AT5G11770	
AT3G48680	AtCAL2	AT5G13450	ATP5
AT3G48680	AtCAL2	AT5G18800	
AT3G48680	AtCAL2	AT5G37510	CI76
AT3G48680	AtCAL2	AT5G40810	

AT3G48680	AtCAL2	AT5G52840	
AT3G48680	AtCAL2	AT5G62740	ATHIR1
AT3G48680	AtCAL2	AT5G66510	GAMMA CA3
AT3G48680	AtCAL2	AT5G67590	FRO1
AT3G48680	AtCAL2	ATMG00070	NAD9
AT3G51460	RHD4	AT3G62360	
AT3G51460	RHD4	AT4G27585	
AT3G51550	FER	AT4G02580	
AT3G51550	FER	AT4G08850	
AT3G51550	FER	AT4G16450	
AT3G51550	FER	AT4G32470	
AT3G51550	FER	AT4G33430	ATBAK1
AT3G51550	FER	AT5G08530	CI51
AT3G51550	FER	AT5G37510	CI76
AT3G51550	FER	AT5G52840	
AT3G51550	FER	AT5G66510	GAMMA CA3
AT3G51550	FER	ATMG00070	NAD9
AT3G52190	AtPHF1	AT3G61070	PEX11E
AT3G52190	AtPHF1	AT5G50460	
AT3G52190	AtPHF1	AT4G29130	ATHXK1
AT3G52730		AT4G34640	ERG9
AT3G52730		AT5G20090	
AT3G53480	ABCG37	AT4G16450	
AT3G53480	ABCG37	AT4G32470	
AT3G53480	ABCG37	AT5G08060	
AT3G53480	ABCG37	AT5G08530	CI51
AT3G53480	ABCG37	AT5G18800	
AT3G53480	ABCG37	AT5G52840	
AT3G53480	ABCG37	ATMG00070	NAD9
AT3G53740		AT4G27090	
AT3G54110	ATPUMP1	ATMG00220	COB
AT3G54110	ATPUMP1	AT3G55320	ABCB20
AT3G54110	ATPUMP1	AT3G60600	(AT)VAP
AT3G54110	ATPUMP1	AT3G62700	ABCC14
AT3G54110	ATPUMP1	AT4G12650	
AT3G54110	ATPUMP1	AT4G21150	HAP6
AT3G54110	ATPUMP1	AT4G24520	AR1
AT3G54110	ATPUMP1	AT4G29900	ACA10
AT3G54110	ATPUMP1	AT4G37640	ACA2
AT3G54110	ATPUMP1	AT4G38350	
AT3G54110	ATPUMP1	AT5G11560	
AT3G54110	ATPUMP1	AT5G13490	AAC2
AT3G54110	ATPUMP1	AT5G15090	ATVDAC3
AT3G54110	ATPUMP1	AT5G19760	
AT3G54110	ATPUMP1	AT5G42570	
AT3G54110	ATPUMP1	AT5G47890	
AT3G54110	ATPUMP1	AT5G56730	
AT3G54110	ATPUMP1	AT5G57490	ATVDAC4
AT3G54110	ATPUMP1	AT5G66680	DGL1
AT3G54110	ATPUMP1	AT5G67500	ATVDAC2
AT3G55320	ABCB20	AT5G19760	
AT3G55320	ABCB20	AT5G56730	

AT3G55360	ATTSC13	AT5G23630	MIA
AT3G56190	ALPHA-SNAP2	AT5G62865	
AT3G57280		AT5G36220	CYP81D1
AT3G57330	ACA11	AT4G01100	ADNT1
AT3G57330	ACA11	AT4G29900	ACA10
AT3G57330	ACA11	AT4G30190	AHA2
AT3G57330	ACA11	AT5G19760	
AT3G57330	ACA11	AT5G37510	CI76
AT3G57330	ACA11	AT5G40810	
AT3G57330	ACA11	AT5G49760	
AT3G57330	ACA11	AT5G51570	
AT3G57330	ACA11	AT5G57110	ACA8
AT3G57330	ACA11	AT5G67590	FRO1
AT3G57330	ACA11	ATMG00070	NAD9
AT3G57650	LPAT2	AT5G40770	ATPHB3
AT3G60600	(AT)VAP	AT4G14070	AAE15
AT3G60600	(AT)VAP	AT4G21150	HAP6
AT3G60600	(AT)VAP	AT4G24520	AR1
AT3G60600	(AT)VAP	AT4G31500	ATR4
AT3G60600	(AT)VAP	AT4G37640	ACA2
AT3G60600	(AT)VAP	AT5G15090	ATVDAC3
AT3G60600	(AT)VAP	AT5G42570	
AT3G60600	(AT)VAP	AT5G56730	
AT3G60600	(AT)VAP	AT5G57490	ATVDAC4
AT3G60600	(AT)VAP	AT5G61790	ATCNX1
AT3G60600	(AT)VAP	AT5G66680	DGL1
AT3G60600	(AT)VAP	AT5G67500	ATVDAC2
AT3G60820	PBF1	AT4G31300	PBA1
AT3G60820	PBF1	AT5G42790	ARS5
AT3G60820	PBF1	AT5G56500	Cpn60beta3
AT3G62360		AT4G17140	
AT3G62360		AT4G27585	
AT3G62360		AT4G28510	ATPHB1
AT3G62360		AT5G05000	ATTOC34
AT3G62360		AT5G11770	
AT3G62360		AT5G19690	STT3A
AT3G62360		AT5G40480	EMB3012
AT3G62360		AT5G40770	ATPHB3
AT3G62360		AT5G42960	
AT3G62360		AT5G46800	BOU
AT3G62360		AT5G47990	CYP705A5
AT3G62700	ABCC14	ATMG00220	COB
AT3G62700	ABCC14	AT5G19760	
AT3G62830	ATUXS2	AT4G17140	
AT3G62830	ATUXS2	AT4G34670	
AT3G62830	ATUXS2	AT5G05000	ATTOC34
AT3G62830	ATUXS2	AT5G25265	
AT3G66654		AT4G26410	
AT3G66658	ALDH22A1	AT4G14070	AAE15
AT4G00550	DGD2	AT4G13010	
AT4G00630	ATKEA2	AT5G11560	
AT4G00630	ATKEA2	AT5G66680	DGL1

AT4G00740	QUA3	AT5G54110	ATMAMI
AT4G00900	ATECA2	AT4G14070	AAE15
AT4G00900	ATECA2	AT4G29130	ATHXK1
AT4G00900	ATECA2	AT4G29330	DER1
AT4G00900	ATECA2	AT5G20660	
AT4G00900	ATECA2	AT5G27540	emb2473
AT4G00900	ATECA2	AT5G58270	ABCB25
AT4G00900	ATECA2	ATMG00160	COX2
AT4G01100	ADNT1	AT5G16590	LRR1
AT4G01100	ADNT1	AT5G19760	
AT4G01320	ATSTE24	AT4G26410	
AT4G01320	ATSTE24	AT5G36220	CYP81D1
AT4G01320	ATSTE24	AT5G52240	AtMAPR5
AT4G02580		AT4G16450	
AT4G02580		AT4G32470	
AT4G02580		AT5G04990	ATSUN1
AT4G02580		AT5G66510	GAMMA CA3
AT4G11150	emb2448	AT4G38510	
AT4G12590		AT5G05370	
AT4G12590		AT4G32400	ATBT1
AT4G12590		AT5G25450	
AT4G12590		AT5G40770	ATPHB3
AT4G12650		AT4G35300	TMT2
AT4G12650		AT4G38350	
AT4G12650		AT5G05520	
AT4G12650		AT5G19760	
AT4G14070	AAE15	AT4G29330	DER1
AT4G14070	AAE15	AT5G27540	emb2473
AT4G14070	AAE15	AT5G40510	
AT4G16450		AT4G32470	
AT4G16450		AT4G33430	ATBAK1
AT4G16450		AT5G08060	
AT4G16450		AT5G08530	CI51
AT4G16450		AT5G11770	
AT4G16450		AT5G18800	
AT4G16450		AT5G37510	CI76
AT4G16450		AT5G49890	ATCLC-C
AT4G16450		AT5G52840	
AT4G16450		AT5G66510	GAMMA CA3
AT4G16450		ATMG00070	NAD9
AT4G17140		AT4G27585	
AT4G17140		AT4G28510	ATPHB1
AT4G17140		AT5G05000	ATTOC34
AT4G17140		AT5G40480	EMB3012
AT4G17140		AT5G40890	ATCLC-A
AT4G20110	BP80-3;1	AT5G04930	ALA1
AT4G20150		AT5G37510	CI76
AT4G21150	HAP6	ATMG00220	COB
AT4G21150	HAP6	AT4G24520	AR1
AT4G21150	HAP6	AT4G37640	ACA2
AT4G21150	HAP6	AT5G11560	
AT4G21150	HAP6	AT5G13490	AAC2

AT4G21150	HAP6	AT5G15090	ATVDAC3
AT4G21150	HAP6	AT5G42570	
AT4G21150	HAP6	AT5G57490	ATVDAC4
AT4G21150	HAP6	AT5G61790	ATCNX1
AT4G21150	HAP6	AT5G64290	DCT
AT4G21150	HAP6	AT5G66680	DGL1
AT4G21150	HAP6	AT5G67500	ATVDAC2
AT4G23430	AtTic32-IVa	AT5G10730	
AT4G24520	AR1	AT4G31500	ATR4
AT4G24520	AR1	AT4G37640	ACA2
AT4G24520	AR1	AT5G15090	ATVDAC3
AT4G24520	AR1	AT5G40510	
AT4G24520	AR1	AT5G42570	
AT4G24520	AR1	AT5G56730	
AT4G24520	AR1	AT5G57490	ATVDAC4
AT4G24520	AR1	AT5G66680	DGL1
AT4G24520	AR1	AT5G67500	ATVDAC2
AT4G26010		AT5G42080	ADL1
AT4G26210		AT4G29480	
AT4G26410		AT4G35000	APX3
AT4G26410		AT5G18480	PGSIP6
AT4G26410		AT5G25100	
AT4G26410		AT5G51220	
AT4G27080	ATPDI7	AT5G55940	emb2731
AT4G27090		AT5G45775	
AT4G27585		AT4G28510	ATPHB1
AT4G27585		AT4G39080	VHA-A3
AT4G27585		AT5G05000	ATTOC34
AT4G27585		AT5G11770	
AT4G27585		AT5G40770	ATPHB3
AT4G27585		AT5G42960	
AT4G27585		AT5G46800	BOU
AT4G28510	ATPHB1	AT4G38240	CGL
AT4G28510	ATPHB1	AT5G11770	
AT4G28510	ATPHB1	AT5G40480	EMB3012
AT4G28510	ATPHB1	AT5G40770	ATPHB3
AT4G28510	ATPHB1	AT5G54100	
AT4G28510	ATPHB1	ATMG00270	NAD6
AT4G29130	ATHXK1	AT5G50460	
AT4G29130	ATHXK1	AT4G29330	DER1
AT4G29130	ATHXK1	AT4G35230	BSK1
AT4G29130	ATHXK1	AT5G08080	ATSYP132
AT4G29130	ATHXK1	AT5G18480	PGSIP6
AT4G29130	ATHXK1	AT5G20660	
AT4G29130	ATHXK1	AT5G25100	
AT4G29130	ATHXK1	AT5G27540	emb2473
AT4G29130	ATHXK1	AT5G58270	ABCB25
AT4G29130	ATHXK1	ATMG00160	COX2
AT4G29480		AT5G20090	
AT4G29900	ACA10	AT5G13490	AAC2
AT4G29900	ACA10	AT5G19760	
AT4G29900	ACA10	AT5G57110	ACA8

AT4G29900	ACA10	ATMG00070	NAD9
AT4G30190	AHA2	AT5G40810	
AT4G30190	AHA2	AT5G51570	
AT4G30190	AHA2	AT5G57110	ACA8
AT4G30260		AT5G06320	NHL3
AT4G31300	PBA1	AT5G42790	ARS5
AT4G31300	PBA1	AT5G56500	Cpn60beta3
AT4G31500	ATR4	AT4G35230	BSK1
AT4G31500	ATR4	AT5G20660	
AT4G31500	ATR4	AT5G27540	emb2473
AT4G31500	ATR4	AT5G35460	
AT4G31500	ATR4	AT5G40510	
AT4G31500	ATR4	AT5G42570	
AT4G31500	ATR4	AT5G42590	CYP71A16
AT4G31500	ATR4	AT5G52240	AtMAPR5
AT4G32400	ATBT1	AT4G38920	ATVHA-C3
AT4G32400	ATBT1	AT5G11560	
AT4G32400	ATBT1	AT5G13490	AAC2
AT4G32400	ATBT1	AT5G14950	ATGMII
AT4G32400	ATBT1	AT5G67500	ATVDAC2
AT4G32410	AtCESA1	AT5G13450	ATP5
AT4G32410	AtCESA1	AT5G18800	
AT4G32470		AT5G08060	
AT4G32470		AT5G08530	CI51
AT4G32470		AT5G11770	
AT4G32470		AT5G18800	
AT4G32470		AT5G37510	CI76
AT4G32470		AT5G52840	
AT4G32470		AT5G66510	GAMMA CA3
AT4G32470		ATMG00070	NAD9
AT4G33430	ATBAK1	AT5G11770	
AT4G33865		AT5G50650	
AT4G35000	APX3	AT5G43970	ATTOM22-V
AT4G35000	APX3	AT5G58270	ABCB25
AT4G35230	BSK1	AT5G07910	
AT4G35230	BSK1	AT5G18480	PGSIP6
AT4G35230	BSK1	AT5G27540	emb2473
AT4G35230	BSK1	AT5G42590	CYP71A16
AT4G35230	BSK1	AT5G52240	AtMAPR5
AT4G35300	TMT2	AT4G38350	
AT4G35300	TMT2	AT5G57350	AHA3
AT4G36750		AT5G52240	AtMAPR5
AT4G37640	ACA2	AT5G11560	
AT4G37640	ACA2	AT5G13490	AAC2
AT4G37640	ACA2	AT5G15090	ATVDAC3
AT4G37640	ACA2	AT5G40510	
AT4G37640	ACA2	AT5G48000	CYP708 A2
AT4G37640	ACA2	AT5G66680	DGL1
AT4G37640	ACA2	AT5G67500	ATVDAC2
AT4G38240	CGL	AT5G19690	STT3A
AT4G38240	CGL	AT5G40480	EMB3012
AT4G38350		ATMG00220	COB

AT4G38350		AT5G14040	PHT3;1
AT4G38350		AT5G19760	
AT4G38350		AT5G27540	emb2473
AT4G38350		AT5G42590	CYP71A16
AT4G38350		AT5G56730	
AT4G38350		AT5G57110	ACA8
AT4G38350		ATMG00160	COX2
AT4G39080	VHA-A3	AT5G11770	
AT4G39080	VHA-A3	AT5G47990	CYP705A5
AT4G39080	VHA-A3	ATMG00270	NAD6
AT5G01500	TAAC	AT5G36220	CYP81D1
AT5G01500	TAAC	AT5G42270	FTSH5
AT5G04830		AT5G42150	
AT5G04930	ALA1	AT5G16590	LRR1
AT5G04930	ALA1	AT5G49760	
AT5G04930	ALA1	AT5G57110	ACA8
AT5G05000	ATTOC34	AT5G23630	MIA
AT5G05000	ATTOC34	AT5G40480	EMB3012
AT5G05000	ATTOC34	AT5G42960	
AT5G05370		AT5G40770	ATPHB3
AT5G07090		AT5G15200	
AT5G08060		AT5G08530	CI51
AT5G08060		AT5G11770	
AT5G08060		AT5G18800	
AT5G08060		AT5G52840	
AT5G08060		AT5G66510	GAMMA CA3
AT5G08530	CI51	AT5G11770	
AT5G08530	CI51	AT5G13450	ATP5
AT5G08530	CI51	AT5G18800	
AT5G08530	CI51	AT5G37510	CI76
AT5G08530	CI51	AT5G40810	
AT5G08530	CI51	AT5G52840	
AT5G08530	CI51	AT5G66510	GAMMA CA3
AT5G08530	CI51	AT5G67590	FRO1
AT5G08530	CI51	ATMG00070	NAD9
AT5G08540		AT5G09995	
AT5G11560		ATMG00220	COB
AT5G11560		AT5G13490	AAC2
AT5G11560		AT5G19760	
AT5G11560		AT5G66680	DGL1
AT5G11560		AT5G67500	ATVDAC2
AT5G11770		AT5G40770	ATPHB3
AT5G11770		AT5G46800	BOU
AT5G11770		AT5G52840	
AT5G11770		AT5G54100	
AT5G11770		AT5G66510	GAMMA CA3
AT5G11770		ATMG00070	NAD9
AT5G12470		AT5G36220	CYP81D1
AT5G13450	ATP5	AT5G18800	
AT5G13450	ATP5	AT5G37510	CI76
AT5G13450	ATP5	AT5G51570	
AT5G13450	ATP5	AT5G52840	

AT5G13450	ATP5	AT5G66510	GAMMA CA3
AT5G13450	ATP5	ATMG00070	NAD9
AT5G13490	AAC2	ATMG00220	COB
AT5G13490	AAC2	AT5G14950	ATGMII
AT5G13490	AAC2	AT5G15090	ATVDAC3
AT5G13490	AAC2	AT5G19760	
AT5G13490	AAC2	AT5G46800	BOU
AT5G13490	AAC2	AT5G47890	
AT5G13490	AAC2	AT5G48000	CYP708 A2
AT5G13490	AAC2	AT5G66680	DGL1
AT5G13490	AAC2	AT5G67500	ATVDAC2
AT5G14040	PHT3;1	AT5G50460	
AT5G14040	PHT3;1	AT5G27540	emb2473
AT5G14040	PHT3;1	AT5G42590	CYP71A16
AT5G14950	ATGMII	AT5G40810	
AT5G15090	ATVDAC3	AT5G42570	
AT5G15090	ATVDAC3	AT5G57490	ATVDAC4
AT5G15090	ATVDAC3	AT5G61790	ATCNX1
AT5G15090	ATVDAC3	AT5G64290	DCT
AT5G15090	ATVDAC3	AT5G66680	DGL1
AT5G15090	ATVDAC3	AT5G67500	ATVDAC2
AT5G16590	LRR1	AT5G19760	
AT5G18380		AT5G45750	AtRABA1c
AT5G18480	PGSIP6	AT5G25100	
AT5G18480	PGSIP6	AT5G27540	emb2473
AT5G18480	PGSIP6	AT5G52240	AtMAPR5
AT5G18800		AT5G37510	CI76
AT5G18800		AT5G52840	
AT5G18800		AT5G66510	GAMMA CA3
AT5G18800		ATMG00070	NAD9
AT5G19690	STT3A	AT5G40480	EMB3012
AT5G19760		ATMG00220	COB
AT5G19760		AT5G40810	
AT5G19760		AT5G56730	
AT5G19760		AT5G57110	ACA8
AT5G20490	ATXIK	AT5G47200	ATRAB1A
AT5G20660		AT5G27540	emb2473
AT5G20660		AT5G42590	CYP71A16
AT5G23300	PYRD	AT5G39410	
AT5G23300	PYRD	AT5G43970	ATTOM22-V
AT5G23630	MIA	AT5G40480	EMB3012
AT5G25100		AT5G52240	AtMAPR5
AT5G27540	emb2473	AT5G50460	
AT5G27540	emb2473	AT5G52240	AtMAPR5
AT5G27540	emb2473	ATMG00160	COX2
AT5G35460		AT5G66680	DGL1
AT5G37510	CI76	AT5G40810	
AT5G37510	CI76	AT5G49760	
AT5G37510	CI76	AT5G51570	
AT5G37510	CI76	AT5G52840	
AT5G37510	CI76	AT5G57110	ACA8
AT5G37510	CI76	AT5G66510	GAMMA CA3

AT5G37510	CI76	AT5G67590	FRO1
AT5G37510	CI76	ATMG00070	NAD9
AT5G39410		AT5G43970	ATTOM22-V
AT5G40480	EMB3012	AT5G40890	ATCLC-A
AT5G40510		AT5G42570	
AT5G40510		AT5G56730	
AT5G40770	ATPHB3	AT5G46800	BOU
AT5G40770	ATPHB3	ATMG00270	NAD6
AT5G40810		AT5G64030	
AT5G40810		AT5G67590	FRO1
AT5G40810		ATMG00070	NAD9
AT5G42150		AT5G42650	AOS
AT5G42270	FTSH5	AT5G51220	
AT5G42570		AT5G61790	ATCNX1
AT5G42570		AT5G66680	DGL1
AT5G42790	ARS5	AT5G56500	Cpn60beta3
AT5G43010	RPT4A	AT5G58290	RPT3
AT5G46800	BOU	AT5G54100	
AT5G47890		AT5G48000	CYP708 A2
AT5G47890		AT5G66680	DGL1
AT5G47890		AT5G67500	ATVDAC2
AT5G49760		AT5G51570	
AT5G49760		AT5G57110	ACA8
AT5G49760		AT5G67590	FRO1
AT5G50460		AT5G52240	AtMAPR5
AT5G50460		ATMG00160	COX2
AT5G50650		AT5G66680	DGL1
AT5G51570		AT5G57110	ACA8
AT5G52240	AtMAPR5	ATMG00160	COX2
AT5G52840		AT5G66510	GAMMA CA3
AT5G52840		ATMG00070	NAD9
AT5G57490	ATVDAC4	AT5G66680	DGL1
AT5G58270	ABCB25	ATMG00160	COX2
AT5G61790	ATCNX1	AT5G66680	DGL1
AT5G61790	ATCNX1	AT5G67500	ATVDAC2
AT5G64290	DCT	AT5G66680	DGL1
AT5G64290	DCT	AT5G67500	ATVDAC2
AT5G66510	GAMMA CA3	ATMG00070	NAD9
AT5G66680	DGL1	AT5G67500	ATVDAC2
AT5G67590	FRO1	ATMG00070	NAD9

Table S2. List of 965 Arabidopsis membrane genes analyzed for the interaction of their encoded protein [6] and used in this study to cluster root protoplasts and nuclei according to their transcriptional activities.

Protein ID	Name	Mapman	DESCRIPTION
AT3G08580	AAC1	transport.metabolite transporters at the envelope membrane	AAC1 (ADP/ATP CARRIER 1); ATP:ADP antiporter/ binding
AT5G13490	AAC2	transport.unspecified cations	AAC2 (ADP/ATP carrier 2); ATP:ADP antiporter/ binding

AT4G14070	AAE15	lipid metabolism.FA synthesis and FA elongation.acyl coa ligase	AAE15 (acyl-activating enzyme 15); long-chain-fatty-acid-[acyl-carrier-protein] ligase
AT2G36910	ABCB1	transport.ABC transporters and multidrug resistance systems	ABCB1 (ATP BINDING CASSETTE SUBFAMILY B1); ATPase, coupled to transmembrane movement of substances / auxin efflux transmembrane transporter/ calmodulin binding
AT3G28860	ABCB19	transport.ABC transporters and multidrug resistance systems	ABCB19; ATPase, coupled to transmembrane movement of substances / auxin efflux transmembrane transporter
AT2G47000	ABCB4	transport.ABC transporters and multidrug resistance systems	ABCB4 (ATP BINDING CASSETTE SUBFAMILY B4); ATPase, coupled to transmembrane movement of substances / xenobiotic-transporting ATPase
AT1G27770	ACA1	transport.calcium	ACA1 (AUTO-INHIBITED CA2+-ATPASE 1); calcium channel/ calcium-transporting ATPase/ calmodulin binding
AT4G29900	ACA10	signalling.calcium	ACA10 (AUTOINHIBITED CA(2+)-ATPASE 10); calcium-transporting ATPase/ calmodulin binding
AT3G57330	ACA11	signalling.calcium	ACA11 (autoinhibited Ca2+-ATPase 11); calcium-transporting ATPase/ calmodulin binding
AT4G37640	ACA2	signalling.calcium	ACA2 (CALCIUM ATPASE 2); calcium ion transmembrane transporter/ calcium-transporting ATPase/ calmodulin binding
AT2G41560	ACA4	signalling.calcium	ACA4 (AUTO-INHIBITED CA(2+)-ATPASE, ISOFORM 4); calcium-transporting ATPase/ calmodulin binding
AT5G57110	ACA8	transport.calcium	ACA8 (AUTOINHIBITED CA2+ -ATPASE, ISOFORM 8); calcium-transporting ATPase/ calmodulin binding / protein self-association
AT3G21180	ACA9	signalling.calcium	ACA9 (AUTOINHIBITED CA(2+)-ATPASE 9); calcium-transporting ATPase/ calmodulin binding
AT5G42080	ADL1	signalling.G-proteins	ADL1 (ARABIDOPSIS DYNAMIN-LIKE PROTEIN); GTP binding / GTPase/ protein binding
AT1G14830	ADL1C	misc.dynamin	ADL1C (ARABIDOPSIS DYNAMIN-LIKE PROTEIN 1C); GTP binding / GTPase
AT3G60190	ADL1E	misc.dynamin	ADL1E (ARABIDOPSIS DYNAMIN-LIKE 1E); GTP binding / GTPase

AT1G59610	ADL3	misc.dynamin	ADL3 (ARABIDOPSIS DYNAMIN-LIKE 3); GTPase
AT4G01100	ADNT1	transport.metabolite transporters at the mitochondrial membrane	ADNT1 (ADENINE NUCLEOTIDE TRANSPORTER 1); ADP transmembrane transporter/ AMP transmembrane transporter/ ATP transmembrane transporter/ binding
AT2G38960	AERO2	protein.targeting.secretory pathway.ER	AERO2 (Arabidopsis endoplasmic reticulum oxidoreductins 2); FAD binding / electron carrier/ oxidoreductase, acting on sulfur group of donors, disulfide as acceptor / protein binding
AT3G22942	AGG2	signalling.G-proteins	AGG2 (G-PROTEIN GAMMA SUBUNIT 2)
AT1G28290	AGP31	stress.abiotic.unspecified	AGP31 (arabinogalactan-protein 31)
AT2G18960	AHA1	transport.p- and v-ATPases.H+-exporting ATPase	AHA1 (ARABIDOPSIS H+ ATPASE 1); ATPase/ hydrogen-exporting ATPase, phosphorylative mechanism / protein binding
AT4G30190	AHA2	transport.p- and v-ATPases.H+-exporting ATPase	AHA2; ATPase/ hydrogen-exporting ATPase, phosphorylative mechanism
AT5G57350	AHA3	transport.p- and v-ATPases.H+-exporting ATPase	AHA3; ATPase/ hydrogen-exporting ATPase, phosphorylative mechanism
AT3G60330	AHA7	transport.p- and v-ATPases	AHA7 (Arabidopsis H(+)-ATPase 7); hydrogen-exporting ATPase, phosphorylative mechanism
AT5G04930	ALA1	transport.p- and v-ATPases	ALA1 (aminophospholipid ATPase1); ATPase, coupled to transmembrane movement of ions, phosphorylative mechanism
AT1G59820	ALA3	transport.p- and v-ATPases	ALA3 (Aminophospholipid ATPase3); ATPase, coupled to transmembrane movement of ions, phosphorylative mechanism / phospholipid transporter
AT3G66658	ALDH22A1	secondary metabolism.N misc.betaine.betaine-aldehyde dehydrogenase	ALDH22a1 (Aldehyde Dehydrogenase 22a1); 3-chloroallyl aldehyde dehydrogenase/ oxidoreductase
AT4G36250	ALDH3F1	fermentation.aldehyde dehydrogenase	ALDH3F1 (Aldehyde Dehydrogenase 3F1); 3-chloroallyl aldehyde dehydrogenase/ aldehyde dehydrogenase (NAD)
AT1G44170	ALDH3H1	fermentation.aldehyde dehydrogenase	ALDH3H1 (ALDEHYDE DEHYDROGENASE 3H1); 3-chloroallyl aldehyde

			dehydrogenase/ aldehyde dehydrogenase (NAD)
AT3G56190	ALPHA-SNAP2	protein.targeting.secretory pathway.unspecified	ALPHA-SNAP2 (ALPHA-SOLUBLE NSF ATTACHMENT PROTEIN 2); binding / soluble NSF attachment protein
AT3G24300	AMT1;3	transport.ammonium	AMT1;3 (AMMONIUM TRANSPORTER 1;3); ammonium transmembrane transporter
AT2G38750	ANNAT4	cell.organisation	ANNAT4 (ANNEXIN ARABIDOPSIS 4); calcium ion binding / calcium-dependent phospholipid binding
AT3G25780	AOC3	hormone metabolism.jasmonate.synthesis-degradation.allene oxidase cyclase	AOC3 (ALLENE OXIDE CYCLASE 3); allene-oxide cyclase
AT5G42650	AOS	hormone metabolism.jasmonate.synthesis-degradation.allene oxidase synthase	AOS (ALLENE OXIDE SYNTHASE); allene oxide synthase/ hydro-lyase/ oxygen binding
AT3G22370	AOX1A	mitochondrial electron transport / ATP synthesis.alternative oxidase	AOX1A (ALTERNATIVE OXIDASE 1A); alternative oxidase
AT4G35000	APX3	redox.ascorbate and glutathione.ascorbate	APX3 (ASCORBATE PEROXIDASE 3); L-ascorbate peroxidase
AT1G06400	ARA-2	signalling.G-proteins	ARA-2; GTP binding
AT2G43130	ARA-4	signalling.G-proteins	ARA4; GTP binding / GTPase/ protein binding
AT1G02130	ARA-5	signalling.G-proteins	ARA-5 (ARABIDOPSIS RAS 5); GTP binding
AT1G43170	ARP1	protein.synthesis.ribosomal protein.eukaryotic.60S subunit.L3	ARP1 (ARABIDOPSIS RIBOSOMAL PROTEIN 1); structural constituent of ribosome
AT1G47290	3BETAHSD/D1	lipid metabolism.'exotics' (steroids, squalene etc).3-beta hydroxysteroid dehydrogenase/isomerase	AT3BETAHSD/D1 (3BETA-HYDROXYSTEROID-DEHYDROGENASE/DECARBOXYLASE ISOFORM 1); 3-beta-hydroxy-delta5-steroid dehydrogenase/ sterol-4-alpha-carboxylate 3-dehydrogenase (decarboxylating)
AT4G01660	ABC1	mitochondrial electron transport / ATP synthesis.unspecified	ATABC1 (ARABIDOPSIS THALIANA ABC TRANSPORTER 1); transporter
AT1G64780	AMT1;2	transport.ammonium	ATAMT1;2 (AMMONIUM TRANSPORTER 1;2); ammonium transmembrane transporter
AT5G67560	ARLA1D	protein.targeting.secretory pathway.unspecified	ATARLA1D (ADP-ribosylation factor-like A1D); GTP binding
AT5G62390	ATBAG7	signalling.calcium	ATBAG7 (ARABIDOPSIS THALIANA BCL-2-ASSOCIATED ATHANOGENE 7); calmodulin binding

AT4G34490	ATCAP1	DNA.unspecified	ATCAP1 (ARABIDOPSIS THALIANA CYCLASE ASSOCIATED PROTEIN 1); actin binding
AT3G18480	AtCASP	protein.targeting.secretory pathway.golgi	AtCASP (Arabidopsis thaliana CCAAT-displacement protein alternatively spliced product)
AT5G17770	ATCBR	redox.misc	ATCBR (ARABIDOPSIS THALIANA NADH:CYTOCHROME B5 REDUCTASE 1); cytochrome-b5 reductase
AT5G40890	ATCLC-A	transport.unspecified anions	ATCLC-A (CHLORIDE CHANNEL A); anion channel/ nitrate transmembrane transporter/ voltage-gated chloride channel
AT2G30920	ATCOQ3	Co-factor and vitamine metabolism.ubiquinone.hexaprenyldihydroxybenzoate methyltransferase	ATCOQ3 (ARABDIOPSIS THALIANA COENZYME Q 3); hexaprenyldihydroxybenzoate methyltransferase/ polyprenyldihydroxybenzoate methyltransferase
AT1G55850	ATCSLE1	cell wall.cellulose synthesis.cellulose synthase	ATCSLE1; cellulose synthase/ transferase, transferring glycosyl groups
AT5G23530	AtCXE18	Biodegradation of Xenobiotics	AtCXE18 (Arabidopsis thaliana carboxyesterase 18); carboxylesterase
AT1G32210	ATDAD1	stress.biotic	ATDAD1 (DEFENDER AGAINST APOPTOTIC DEATH 1)
AT1G79940	ATERDJ2A	stress.abiotic.heat	ATERDJ2A; heat shock protein binding / unfolded protein binding
AT5G64440	AtFAAH	not assigned.no ontology	AtFAAH (Arabidopsis thaliana fatty acid amide hydrolase); N-(long-chain-acyl)ethanolamine deacylase/ amidase
AT5G01600	ATFER1	metal handling.binding, chelation and storage	ATFER1; ferric iron binding / iron ion binding
AT3G11730	ATFP8	signalling.G-proteins	ATFP8; GTP binding / GTP-dependent protein binding / myosin XI tail binding
AT3G51830	ATG5	signalling.phosphinositides	ATG5; phosphatidylinositol-4,5-bisphosphate 5-phosphatase
AT4G35860	ATGB2	signalling.G-proteins	ATGB2 (GTP-BINDING 2); GTP binding
AT1G75680	AtGH9B7	misc.gluco-, galacto- and mannosidases.endoglucanase	AtGH9B7 (Arabidopsis thaliana glycosyl hydrolase 9B7); catalytic/ hydrolase, hydrolyzing O-glycosyl compounds
AT3G47930	ATGLDH	redox.ascorbate and glutathione.ascorbate.L-Galactono-1,4-lactone dehydrogenase	ATGLDH (L-GALACTONO-1,4-LACTONE DEHYDOROGENASE); L-gulono-1,4-lactone dehydrogenase/ galactonolactone dehydrogenase

AT3G63080	ATGPX5	redox.ascorbate and glutathione.glutathione	ATGPX5 (glutathione peroxidase 5); glutathione peroxidase
AT3G17820	ATGSKB6	N-metabolism.ammonia metabolism.glutamine synthetase	ATGSKB6; copper ion binding / glutamate-ammonia ligase
AT4G03550	ATGSL05	minor CHO metabolism.callose	ATGSL05 (GLUCAN SYNTHASE-LIKE 5); 1,3-beta-glucan synthase/ transferase, transferring glycosyl groups
AT3G07160	ATGSL10	minor CHO metabolism.callose	ATGSL10 (glucan synthase-like 10); 1,3-beta-glucan synthase
AT5G37600	ATGLN1;1	N-metabolism.ammonia metabolism.glutamine synthetase	ATGSR1; copper ion binding / glutamate-ammonia ligase
AT1G66200	ATGSR2	N-metabolism.ammonia metabolism.glutamine synthetase	ATGSR2; copper ion binding / glutamate-ammonia ligase
AT1G02950	ATGSTF4	misc.glutathione S transferases	ATGSTF4 (GLUTATHIONE S-TRANSFERASE F4); glutathione transferase
AT3G08710	ATH9	redox.thioredoxin	ATH9 (thioredoxin H-type 9)
AT4G36480	ATLCB1	lipid metabolism.'exotics' (steroids, squalene etc).sphingolipids	ATLCB1 (LONG-CHAIN BASE1); protein binding / serine C-palmitoyltransferase
AT5G54110	ATMAMI	cell.vesicle transport	ATMAMI (ARABIDOPSIS THALIANA MEMBRANE-ASSOCIATED MANNITOL-INDUCED); structural molecule
AT3G43300	ATMIN7	signalling.G-proteins	ATMIN7 (ARABIDOPSIS THALIANA HOPM INTERACTOR 7); guanyl-nucleotide exchange factor/ protein binding
AT3G62700	ABCC14	transport.ABC transporters and multidrug resistance systems	ATMRP10; ATPase, coupled to transmembrane movement of substances
AT2G34660	ABCC2	transport.ABC transporters and multidrug resistance systems	ATMRP2 (ARABIDOPSIS THALIANA MULTIDRUG RESISTANCE-ASSOCIATED PROTEIN 2); ATPase, coupled to transmembrane movement of substances
AT2G47800	ABCC4	transport.ABC transporters and multidrug resistance systems	ATMRP4 (ARABIDOPSIS THALIANA MULTIDRUG RESISTANCE-ASSOCIATED PROTEIN 4); ATPase, coupled to transmembrane movement of substances / folic acid transporter
AT1G15500	ATNTT2	transport.metabolite transporters at the mitochondrial membrane	ATNTT2; ATP:ADP antiporter
AT4G00860	AT0ZI1	stress	ATOZI1
AT2G43080	AT-P4H-1	misc.oxidases - copper, flavone etc	AT-P4H-1 (A. THALIANA P4H ISOFORM 1); oxidoreductase, acting on paired donors, with

			incorporation or reduction of molecular oxygen, 2-oxoglutarate as one donor, and incorporation of one atom each of oxygen into both donors / procollagen-proline 4-dioxygen
AT5G13640	ATPDAT	lipid metabolism.'exotics' (steroids, squalene etc).phosphatidylcholinesterol O-acyltransferase	ATPDAT; phosphatidylcholine-sterol O-acyltransferase
AT2G32920	ATPDI9	redox.thioredoxin.PDIL	ATPDIL2-3 (PDI-LIKE 2-3); protein disulfide isomerase
AT1G35620	ATPDI8	redox.thioredoxin.PDIL	ATPDIL5-2 (PDI-LIKE 5-2); protein disulfide isomerase
AT4G28510	ATPHB1	mitochondrial electron transport / ATP synthesis.NADH-DH.complex I	ATPHB1 (PROHIBITIN 1)
AT1G03860	ATPHB2	cell.cycle	ATPHB2 (PROHIBITIN 2)
AT5G40770	ATPHB3	cell.division	ATPHB3 (PROHIBITIN 3)
AT3G27280	ATPHB4	cell.division	ATPHB4 (PROHIBITIN 4)
AT2G20530	ATPHB6	mitochondrial electron transport / ATP synthesis.NADH-DH.complex I	ATPHB6 (PROHIBITIN 6)
AT3G08510	ATPLC2	signalling.phosphinositides.phosphoinositide phospholipase C	ATPLC2 (PHOSPHOLIPASE C 2); phospholipase C
AT4G35790	ATPLDDELTA	lipid metabolism.lipid degradation.lysophospholipases.phospholipase D	ATPLDDELTA; phospholipase D
AT3G52300	ATPQ	mitochondrial electron transport / ATP synthesis.F1-ATPase	ATPQ (ATP SYNTHASE D CHAIN, MITOCHONDRIAL); hydrogen ion transmembrane transporter
AT2G38940	ATPT2	transport.phosphate	ATPT2 (ARABIDOPSIS THALIANA PHOSPHATE TRANSPORTER 2); carbohydrate transmembrane transporter/ inorganic phosphate transmembrane transporter/ phosphate transmembrane transporter/ sugar:hydrogen symporter
AT4G24520	AR1	misc.cytochrome P450	ATR1 (ARABIDOPSIS P450 REDUCTASE 1); NADPH-hemoprotein reductase
AT4G30210	AR2	misc.cytochrome P450	ATR2 (ARABIDOPSIS P450 REDUCTASE 2); NADPH-hemoprotein reductase
AT1G09630	ATRAB-A2A	signalling.G-proteins	ATRAB11C (ARABIDOPSIS RAB GTPASE 11C); GTP binding

AT1G43890	ATRAB-C1	signalling.G-proteins	ATRAB18 (ARABIDOPSIS RAB GTPASE HOMOLOG B18); GTP binding
AT5G47200	ATRAB1A	signalling.G-proteins	ATRAB1A; GTP binding
AT2G21880	ATRAB7A	signalling.G-proteins	ATRAB7A; GTP binding
AT3G18820	ATRAB7B	signalling.G-proteins	ATRAB7B (ARABIDOPSIS RAB GTPASE HOMOLOG G3F); GTP binding
AT3G53610	ATRAB8	signalling.G-proteins	ATRAB8; GTP binding
AT3G46060	ARA-3	signalling.G-proteins	ATRAB8A; GTP binding
AT5G03520	ATRAB-E1D	signalling.G-proteins	ATRAB8C; GTP binding
AT1G16920	ATRABA1B	signalling.G-proteins	ATRABA1B (ARABIDOPSIS RAB GTPASE HOMOLOG A1B); GTP binding
AT5G45750	AtRABA1c	signalling.G-proteins	AtRABA1c (Arabidopsis Rab GTPase homolog A1c); GTP binding
AT4G18800	ATHSGBP	signalling.G-proteins	ATRABA1D (ARABIDOPSIS RAB GTPASE HOMOLOG A1D); GTP binding
AT4G18430	AtRABA1e	signalling.G-proteins	AtRABA1e (Arabidopsis Rab GTPase homolog A1e); GTP binding
AT5G59150	ATRAB-A2D	signalling.G-proteins	ATRABA2D (HOARABIDOPSIS RAB GTPASE HOMOLOG A2D); GTP binding
AT2G21600	ATRER1B	protein.targeting.secretory pathway.ER	ATRER1B
AT1G28340	AtRLP4	not assigned.no ontology	AtRLP4 (Receptor Like Protein 4); protein binding
AT2G37270	ATRPS5B	protein.synthesis.ribosomal protein.eukaryotic.40S subunit.S5	ATRPS5B (RIBOSOMAL PROTEIN 5B); structural constituent of ribosome
AT4G30580	ATS2	development.unspecified	ATS2; 1-acylglycerol-3-phosphate O-acyltransferase/ acyltransferase
AT1G56330	ATSAR1	signalling.G-proteins	ATSAR1B (SECRETION-ASSOCIATED RAS 1 B); GTP binding
AT4G01320	ATSTE24	protein.degradation	ATSTE24; endopeptidase/ metalloendopeptidase
AT5G39040	ABCB27	transport.ABC transporters and multidrug resistance systems	ATTAP2; ATPase, coupled to transmembrane movement of substances / transporter
AT3G20050	ATTCP-1	protein.folding	ATTCP-1; ATP binding / protein binding / unfolded protein binding
AT1G72750	ATTIM23-2	protein.targeting.mitochondria	ATTIM23-2; P-P-bond-hydrolysis-driven protein transmembrane transporter/ protein transporter

AT2G36070	ATTIM44-2	protein.targeting.mitochondria	ATTIM44-2; protein-transmembrane transporting ATPase
AT4G03560	ATCCH1	transport.calcium	ATTPC1 (TWO-PORE CHANNEL 1); calcium channel/ voltage-gated calcium channel
AT5G39950	ATH2	redox.thioredoxin	ATTRX2 (THIOREDOXIN 2); oxidoreductase, acting on sulfur group of donors, disulfide as acceptor
AT5G22360	ATVAMP714	cell.vesicle transport	ATVAMP714 (VESICLE-ASSOCIATED MEMBRANE PROTEIN 714)
AT3G54300	ATVAMP727	cell.vesicle transport	ATVAMP727 (VESICLE-ASSOCIATED MEMBRANE PROTEIN 727)
AT4G38920	ATVHA-C3	transport.p- and v-ATPases.H+-transporting two-sector ATPase.subunit C	ATVHA-C3 (VACUOLAR-TYPE H(+)-ATPASE C3); ATPase
AT2G05170	ATVPS11	protein.targeting.secretory pathway.vacuole	ATVPS11; binding / protein binding / transporter/ zinc ion binding
AT2G14740	ATVSR3	protein.targeting.secretory pathway.vacuole	ATVSR3 (ARABIDOPSIS THALIANA VACULOLAR SORTING RECEPTOR 3); calcium ion binding
AT3G62830	ATUXS2	cell wall.precursor synthesis.UXS	AUD1; UDP-glucuronate decarboxylase/ catalytic/ dTDP-glucose 4,6-dehydratase
AT2G38120	AUX1	transport.misc	AUX1 (AUXIN RESISTANT 1); amino acid transmembrane transporter/ auxin binding / auxin influx transmembrane transporter/ transporter
AT1G15690	ATAVP3	transport.H+ transporting pyrophosphatase	AVP1; ATPase/ hydrogen-translocating pyrophosphatase
AT1G78920	AtVHP2;1	transport.H+ transporting pyrophosphatase	AVP2 (ARABIDOPSIS VACUOLAR H+-PYROPHOSPHATASE 2); hydrogen-translocating pyrophosphatase
AT1G54990	AXR4	not assigned.unknown	AXR4 (AUXIN RESISTANT 4)
AT4G33430	ATBAK1	signalling.receptor kinases.leucine rich repeat.brassinosteroid signaling.SERK.BAK1	BAK1 (BRI1-ASSOCIATED RECEPTOR KINASE); kinase/ protein binding / protein heterodimerization/ protein serine/threonine kinase
AT5G64370	BETA-UP	nucleotide metabolism.degradation.pyrimidine.beta-ureidopropionase	BETA-UP (beta-ureidopropionase); beta-ureidopropionase
AT3G02260	ASA1	hormone metabolism.auxin.signal transduction	BIG (BIG); binding / ubiquitin-protein ligase/ zinc ion binding

AT5G61900	BON	signalling.calcium	BON1 (BONZAI 1); calcium-dependent phospholipid binding
AT5G46800	BOU	transport.metabolite transporters at the mitochondrial membrane	BOU (A BOUT DE SOUFFLE); binding / transporter
AT5G16840	BPA1	RNA.RNA binding	BPA1 (BINDINGPARTNEROFACD11 1); nucleic acid binding / nucleotide binding / oxidoreductase
AT4G35230	BSK1	signalling.kinase.RLCK.brassinosteroid signalling kinase	BSK1 (BR-SIGNALING KINASE 1); ATP binding / binding / kinase/ protein kinase/ protein tyrosine kinase
AT4G00710	BSK3	signalling.kinase.RLCK.brassinosteroid signalling kinase	BSK3 (BR-SIGNALING KINASE 3); ATP binding / binding / kinase/ protein kinase/ protein tyrosine kinase
AT4G23630	BTI1	stress.biotic	BTI1 (VIRB2-INTERACTING PROTEIN 1)
AT4G11220	BTI2	not assigned.no ontology	BTI2 (VIRB2-INTERACTING PROTEIN 2)
AT5G41600	BTI3	not assigned.no ontology	BTI3 (VIRB2-INTERACTING PROTEIN 3)
AT2G30490	ATC4H	secondary metabolism.phenylpropanoids.lignin biosynthesis.C4H	C4H (CINNAMATE-4-HYDROXYLASE); trans-cinnamate 4-monooxygenase
AT1G29930	AB140	PS.lightreaction.photosystem II.LHC-II	CAB1 (CHLOROPHYLL A/B BINDING PROTEIN 1); chlorophyll binding
AT1G07670	ATECA4	transport.calcium	calcium-transporting ATPase
AT5G61790	ATCNX1	signalling.calcium	calnexin 1 (CNX1)
AT1G05570	ATGSL06	minor CHO metabolism.callose	CALS1 (CALLOSE SYNTHASE 1); 1,3-beta-glucan synthase/ transferase, transferring glycosyl groups
AT2G07050	CAS1	hormone metabolism.brassinosteroid.synthesis-degradation.sterols.other	CAS1 (cycloartenol synthase 1); cycloartenol synthase
AT1G05940	CAT9	transport.amino acids	CAT9 (CATIONIC AMINO ACID TRANSPORTER 9); cationic amino acid transmembrane transporter
AT1G26340	ATCB5-A	redox.ascorbate and glutathione	CB5-A (CYTOCHROME B5 ISOFORM A); heme binding
AT2G32720	ATCB5-B	redox.ascorbate and glutathione	CB5-B (CYTOCHROME B5 ISOFORM B); heme binding
AT2G46650	ATCB5-C	redox.ascorbate and glutathione	CB5-C (CYTOCHROME B5 ISOFORM C); heme binding
AT5G48810	ATB5-B	redox.ascorbate and glutathione	CB5-D (CYTOCHROME B5 ISOFORM D); heme binding
AT5G53560	ATB5-A	redox.misc	CB5-E (CYTOCHROME B5 ISOFORM E); heme binding

AT1G30450	ATCCC1	transport.unspecified anions	CCC1 (CATION-CHLORIDE CO-TRANSPORTER 1); cation:chloride symporter/ sodium:potassium:chloride symporter
AT3G63520	ATCCD1	secondary metabolism.isoprenoids.carotenoids.carotenoid cleavage dioxygenase	CCD1 (CAROTENOID CLEAVAGE DIOXYGENASE 1); 9-cis-epoxycarotenoid dioxygenase
AT3G09840	ATCDC48	cell.division	CDC48 (CELL DIVISION CYCLE 48); ATPase/ identical protein binding
AT5G19450	CDPK19	signalling.calcium.calcium dependent protein kinases	CDPK19 (CALCIUM-DEPENDENT PROTEIN KINASE 19); ATP binding / calcium ion binding / calmodulin-dependent protein kinase/ kinase/ protein kinase/ protein serine/threonine kinase
AT3G55360	ATTSC13	lipid metabolism.'exotics' (steroids, squalene etc).trans-2-enoyl-CoA reductase (NADPH)	CER10; 3-oxo-5-alpha-steroid 4-dehydrogenase/ fatty acid elongase/ trans-2-enoyl-CoA reductase (NADPH)
AT3G21630	CERK1	signalling.receptor kinases.leucine rich repeat IX	CERK1 (CHITIN ELICITOR RECEPTOR KINASE 1); kinase/ receptor signaling protein/ transmembrane receptor protein kinase
AT4G32410	AtCESA1	cell wall.cellulose synthesis.cellulose synthase	CESA1 (CELLULOSE SYNTHASE 1); cellulose synthase/ transferase, transferring glycosyl groups
AT5G05170	ATCESA3	cell wall.cellulose synthesis.cellulose synthase	CEV1 (CONSTITUTIVE EXPRESSION OF VSP 1); cellulose synthase/ transferase, transferring glycosyl groups
AT4G38240	CGL	protein.glycosylation.alpha-1,3-mannosyl-glycoprotein-beta-1,2-N-acetylglucosaminyltransferase(GnTI)	CGL1 (COMPLEX GLYCAN LESS 1); alpha-1,3-mannosylglycoprotein 2-beta-N-acetylglucosaminyltransferase/ protein N-acetylglucosaminyltransferase/ transferase, transferring glycosyl groups
ATCG00480	ATPB	PS.lightreaction.ATP synthase.beta subunit	chloroplast-encoded gene for beta subunit of ATP synthase
AT2G32260	ATCCT1	lipid metabolism.Phospholipid synthesis.choline-phosphate cytidylyltransferase	cholinephosphate cytidylyltransferase, putative / phosphorylcholine transferase, putative / CTP:phosphocholine cytidylyltransferase, putative
AT5G08530	CI51	mitochondrial electron transport / ATP synthesis.NADH-DH.localisation not clear	CI51 (51 kDa subunit of complex I); 4 iron, 4 sulfur cluster binding / FMN binding / NAD or NADH binding / NADH dehydrogenase

			(ubiquinone)/ oxidoreductase, acting on NADH or NADPH
AT4G24400	ATCIPK8	signalling.calcium.CBL interacting kinase/SnRK3	CIPK8 (CBL-INTERACTING PROTEIN KINASE 8); kinase/ protein kinase
AT5G49890	ATCLC-C	transport.unspecified anions	CLC-C (CHLORIDE CHANNEL C); anion channel/ voltage-gated chloride channel
AT2G20120	COV1	hormone metabolism.auxin.induced-regulated-responsive-activated	COV1 (CONTINUOUS VASCULAR RING)
AT5G56090	COX15	protein.aa activation	COX15 (cytochrome c oxidase 15)
AT3G57530	ATCPK32	signalling.calcium.calcium dependent protein kinases	CPK32 (CALCIUM-DEPENDENT PROTEIN KINASE 32); calcium-dependent protein kinase C/ calmodulin-dependent protein kinase/ kinase/ protein binding
AT2G28000	CH-CPN60A	PS.calvin cycle.rubisco interacting	CPN60A (CHAPERONIN-60ALPHA); ATP binding / protein binding
AT1G55490	CPN60B	PS.calvin cycle.rubisco interacting	CPN60B (CHAPERONIN 60 BETA); ATP binding / protein binding
AT3G03050	ATCSLD3	cell wall.cellulose synthesis.cellulose synthase	CSLD3 (CELLULOSE SYNTHASE-LIKE D3); cellulose synthase/ transferase, transferring glycosyl groups
AT1G11680	CYP51	hormone metabolism.brassinosteroid.synthesis-degradation.sterols.CYP51	CYP51G1 (CYTOCHROME P450 51G1); oxygen binding / sterol 14-demethylase
AT2G45510	CYP704A2	misc.cytochrome P450	CYP704A2; electron carrier/ heme binding / iron ion binding / monooxygenase/ oxygen binding
AT4G15330	CYP705A1	misc.cytochrome P450	CYP705A1; electron carrier/ heme binding / iron ion binding / monooxygenase/ oxygen binding
AT5G42580	CYP705A12	misc.cytochrome P450	CYP705A12; electron carrier/ heme binding / iron ion binding / monooxygenase/ oxygen binding
AT3G20940	CYP705A30	misc.cytochrome P450	CYP705A30; electron carrier/ heme binding / iron ion binding / monooxygenase/ oxygen binding
AT3G20960	CYP705A33	misc.cytochrome P450	CYP705A33; electron carrier/ heme binding / iron ion binding / monooxygenase/ oxygen binding
AT5G47990	CYP705A5	misc.cytochrome P450	CYP705A5; oxygen binding / thalian-diol desaturase
AT5G48000	CYP708A2	misc.cytochrome P450	CYP708A2; oxygen binding / thalianol hydroxylase
AT2G28860	CYP710A4	misc.cytochrome P450	CYP710A4 (cytochrome P450, family 710, subfamily A, polypeptide 4); electron carrier/

			heme binding / iron ion binding / monooxygenase/ oxygen binding
AT5G42590	CYP71A16	misc.cytochrome P450	CYP71A16; electron carrier/ heme binding / iron ion binding / monooxygenase/ oxygen binding
AT1G13080	CYP71B2	misc.cytochrome P450	CYP71B2 (CYTOCHROME P450 71B2); electron carrier/ heme binding / iron ion binding / monooxygenase/ oxygen binding
AT3G26210	CYP71B23	misc.cytochrome P450	CYP71B23; electron carrier/ heme binding / iron ion binding / monooxygenase/ oxygen binding
AT3G26330	CYP71B37	misc.cytochrome P450	CYP71B37; electron carrier/ heme binding / iron ion binding / monooxygenase/ oxygen binding
AT2G24180	CYP71B6	misc.cytochrome P450	CYP71B6 (CYTOCHROME P450 71B6); electron carrier/ heme binding / iron ion binding / monooxygenase/ oxygen binding
AT1G13110	CYP71B7	misc.cytochrome P450	CYP71B7; electron carrier/ heme binding / iron ion binding / monooxygenase/ oxygen binding
AT1G75130	CYP721A1	misc.cytochrome P450	CYP721A1; electron carrier/ heme binding / iron ion binding / monooxygenase/ oxygen binding
AT3G14680	CYP72A14	misc.cytochrome P450	CYP72A14; electron carrier/ heme binding / iron ion binding / monooxygenase/ oxygen binding
AT3G14610	CYP72A7	misc.cytochrome P450	CYP72A7; electron carrier/ heme binding / iron ion binding / monooxygenase/ oxygen binding
AT1G16400	CYP79F2	misc.cytochrome P450	CYP79F2; oxidoreductase, acting on paired donors, with incorporation or reduction of molecular oxygen, NADH or NADPH as one donor, and incorporation of one atom of oxygen / oxygen binding
AT5G36220	CYP81D1	misc.cytochrome P450	CYP81D1 (CYTOCHROME P450 81D1); electron carrier/ heme binding / iron ion binding / monooxygenase/ oxygen binding
AT2G25160	CYP82F1	misc.cytochrome P450	CYP82F1; electron carrier/ heme binding / iron ion binding / monooxygenase/ oxygen binding
AT4G13770	CYP83A1	misc.cytochrome P450	CYP83A1 (CYTOCHROME P450 83A1); oxidoreductase, acting on paired donors, with incorporation or reduction of molecular oxygen, NADH or NADPH as one donor, and incorporation of one atom of oxygen / oxygen binding

AT4G31500	ATR4	misc.cytochrome P450	CYP83B1 (CYTOCHROME P450 MONOOXYGENASE 83B1); oxidoreductase, acting on paired donors, with incorporation or reduction of molecular oxygen, NADH or NADPH as one donor, and incorporation of one atom of oxygen / oxygen binding
AT2G40890	CYP98A3	secondary metabolism.phenylpropanoids.lignin biosynthesis.C3H	CYP98A3 (cytochrome P450, family 98, subfamily A, polypeptide 3); monooxygenase/ p-coumarate 3-hydroxylase
ATMG00160	COX2	mitochondrial electron transport / ATP synthesis.cytochrome c oxidase	cytochrome c oxidase subunit 2
AT4G29330	DER1	protein.degradation	DER1 (DERLIN-1)
AT3G09090	DEX1	development.unspecified	DEX1 (DEFECTIVE IN EXINE FORMATION 1); calcium ion binding
AT4G00550	DGD2	lipid metabolism.glycolipid synthesis.DGDG synthase	DGD2; UDP-galactosyltransferase/ UDP-glycosyltransferase/ digalactosyldiacylglycerol synthase/ transferase, transferring glycosyl groups
AT5G66680	DGL1	protein.glycosylation	DGL1; dolichyl-diphosphooligosaccharide-protein glycotransferase
AT5G12290	DGS1	not assigned.no ontology	DGS1 (DGD1 SUPPRESSOR 1)
AT2G18730	ATDGK3	lipid metabolism.Phospholipid synthesis.diacylglycerol kinase	diacylglycerol kinase, putative
AT5G64290	DCT	transport.metabolite transporters at the mitochondrial membrane	DIT2.1 (DICARBOXYLATE TRANSPORT 2.1); oxoglutarate:malate antiporter
AT1G27980	ATDPL1	lipid metabolism.'exotics' (steroids, squalene etc).sphingolipids	DPL1; carboxy-lyase/ catalytic/ pyridoxal phosphate binding
AT4G20260	ATPCAP1	signalling.phosphinositides	DREPP plasma membrane polypeptide family protein
AT4G33650	ADL2	misc.dynamin	DRP3A (DYNAMIN-RELATED PROTEIN 3A); GTP binding / GTPase/ phosphoinositide binding
AT3G19820	CBB1	hormone metabolism.brassinosteroid.synthesis-degradation.sterols.DWF1	DWF1 (DWARF 1); calmodulin binding / catalytic
AT2G14120	DRP3B	misc.dynamin	dynamin-like protein 2b (ADL2b)
AT1G07810	ACA3	transport.calcium	ECA1 (ER-TYPE CA2+-ATPASE 1); calcium-transporting ATPase
AT4G00900	ATECA2	transport.calcium	ECA2 (ER-TYPE CA2+-ATPASE 2); calcium-transporting ATPase

AT1G10130	ATECA3	transport.calcium	ECA3 (ENDOPLASMIC RETICULUM-TYPE CALCIUM-TRANSPORTING ATPASE 3); calcium-transporting ATPase/ calmodulin binding / manganese-transporting ATPase/ peroxidase
AT1G01960	EDA10	signalling.G-proteins	EDA10 (embryo sac development arrest 10); ARF guanyl-nucleotide exchange factor/ binding / guanyl-nucleotide exchange factor
AT3G56150	ATEIF3C-1	protein.synthesis.initiation	EIF3C (EUKARYOTIC TRANSLATION INITIATION FACTOR 3C); translation initiation factor
AT3G57290	ATEIF3E-1	protein.synthesis.initiation	EIF3E (EUKARYOTIC TRANSLATION INITIATION FACTOR 3E); translation initiation factor
AT5G37510	CI76	mitochondrial electron transport / ATP synthesis.NADH-DH.localisation not clear	EMB1467 (embryo defective 1467); NADH dehydrogenase (ubiquinone)/ NADH dehydrogenase/ electron carrier/ iron-sulfur cluster binding / oxidoreductase/ oxidoreductase, acting on NADH or NADPH
AT1G10510	emb2004	development.unspecified	emb2004 (embryo defective 2004)
AT5G09900	EMB2107	protein.degradation.ubiquitin.proteasom	EMB2107 (EMBRYO DEFECTIVE 2107)
AT3G04400	emb2171	protein.synthesis.ribosomal protein.eukaryotic.60S subunit.L23	emb2171 (embryo defective 2171); structural constituent of ribosome
AT1G20200	EMB2719	protein.degradation.ubiquitin.proteasom	EMB2719 (EMBRYO DEFECTIVE 2719); enzyme regulator
AT5G55940	emb2731	development.unspecified	emb2731 (embryo defective 2731)
AT1G80410	EMB2753	misc.acyl transferases	EMB2753 (EMBRYO DEFECTIVE 2753); binding
AT4G36630	EMB2754	not assigned.unknown	EMB2754 (EMBRYO DEFECTIVE 2754); binding / small GTPase regulator
AT1G34430	EMB3003	TCA / org transformation.TCA.pyruvate DH.E2	EMB3003 (embryo defective 3003); acyltransferase/ dihydrolipoyllysine-residue acetyltransferase/ protein binding
AT5G40480	EMB3012	protein.targeting.nucleus	EMB3012 (embryo defective 3012)
ATMG00480	ATP8	mitochondrial electron transport / ATP synthesis.F1-ATPase	Encodes subunit 8 of the mitochondrial F(O) ATP synthase complex.
ATCG00500	ACCD	lipid metabolism.FA synthesis and FA elongation.Acetyl CoA Carboxylation.heteromeric Complex.beta Carboxyltransferase	Encodes the carboxytransferase beta subunit of the Acetyl-CoA carboxylase (ACCase) complex in plastids. This complex catalyzes the

			carboxylation of acetyl-CoA to produce malonyl-CoA, the first committed step in fatty acid synthesis.
AT1G30360	ERD4	stress.abiotic.drought/salt	ERD4 (early-responsive to dehydration 4)
AT1G08930	ERD6	transport.sugars	ERD6 (EARLY RESPONSE TO DEHYDRATION 6); carbohydrate transmembrane transporter/ sugar transmembrane transporter/ sugar:hydrogen symporter
AT2G17840	ERD7	development.unspecified	ERD7 (EARLY-RESPONSIVE TO DEHYDRATION 7)
AT2G43400	ETFQO	mitochondrial electron transport / ATP synthesis.electron transfer flavoprotein	ETFQO (electron-transfer flavoprotein:ubiquinone oxidoreductase); catalytic/ electron carrier/ electron-transferring-flavoprotein dehydrogenase
AT5G64260	EXL2	signalling.in sugar and nutrient physiology	EXL2 (EXORDIUM LIKE 2)
AT5G26030	ATFC-I	tetrapyrrole synthesis.ferrochelatase	FC1 (ferrochelatase 1); ferrochelatase
AT5G63910	FCLY	not assigned.unknown	FCLY (FARNESYLCYSTEINE LYASE); prenylcysteine oxidase
AT3G51550	FER	signalling.receptor kinases.Catharanthus roseus-like RLK1	FER (FERONIA); kinase/ protein kinase
AT4G25630	ATFIB2	protein.synthesis.ribosome biogenesis.Pre-rRNA processing and modifications.methylotransferases	FIB2 (FIBRILLARIN 2); snoRNA binding
AT5G67590	FRO1	mitochondrial electron transport / ATP synthesis.NADH-DH.localisation not clear	FRO1 (FROSTBITE1); NADH dehydrogenase (ubiquinone)
AT5G23980	ATFRO4	metal handling.acquisition	FRO4 (FERRIC REDUCTION OXIDASE 4); ferric-chelate reductase
AT5G53170	FTSH11	protein.degradation.metalloprotease	FTSH11 (FtsH protease 11); ATP-dependent peptidase/ ATPase/ metallopeptidase
AT2G26140	ftsh4	protein.degradation.metalloprotease	ftsh4 (FtsH protease 4); ATP-dependent peptidase/ ATPase/ metallopeptidase
AT1G14080	ATFUT6	cell wall.cell wall proteins.AGPs.AGP Fucosyltransferase	FUT6 (FUCOSYLTRANSFERASE 6); fucosyltransferase/ transferase, transferring glycosyl groups
AT1G19580	GAMMA CA1	mitochondrial electron transport / ATP synthesis.NADH-DH.complex I.carbonic anhydrase	GAMMA CA1 (GAMMA CARBONIC ANHYDRASE 1); carbonate dehydratase
AT1G47260	APFI	mitochondrial electron transport / ATP synthesis.NADH-DH.complex I.carbonic anhydrase	GAMMA CA2 (GAMMA CARBONIC ANHYDRASE 2); carbonate dehydratase

AT5G66510	GAMMA CA3	mitochondrial electron transport / ATP synthesis.NADH-DH.complex I.carbonic anhydrase	GAMMA CA3 (GAMMA CARBONIC ANHYDRASE 3); carbonate dehydratase
AT5G63510	GAMMA CAL1	mitochondrial electron transport / ATP synthesis.NADH-DH.complex I.carbonic anhydrase	GAMMA CAL1 (GAMMA CARBONIC ANHYDRASE LIKE 1); carbonate dehydratase
AT3G48680	AtCAL2	mitochondrial electron transport / ATP synthesis.NADH-DH.complex I.carbonic anhydrase	GAMMA CAL2 (GAMMA CARBONIC ANHYDRASE-LIKE 2); transferase
AT2G36830	GAMMA-TIP	transport.Major Intrinsic Proteins.TIP	GAMMA-TIP (GAMMA TONOPLAST INTRINSIC PROTEIN); water channel
AT2G19950	GC1	not assigned.unknown	GC1 (Golgin Candidate 1)
AT2G22475	GEM	hormone metabolism.abscisic acid.induced-regulated-responsive-activated	GEM (GL2-EXPRESSION MODULATOR)
AT5G14950	ATGMII	protein.glycosylation.mannosyl-oligosaccharide-1,3-1,6-alpha-mannosidase(GMII)	GMII (GOLGI ALPHA-MANNOSIDASE II); alpha-mannosidase
AT2G26300	ATGPA1	signalling.G-proteins	GP ALPHA 1 (G PROTEIN ALPHA SUBUNIT 1); GTP binding / GTPase/ channel regulator/ signal transducer
AT2G41490	GPT	protein.glycosylation	GPT (UDP-GLCNAC%3ADOLICHOL+PHOSPHATE+GLCNAC-1-P+TRANSFERASE); UDP-N-acetylglucosamine-dolichyl-phosphate N-acetylglucosaminephosphotransferase
AT2G26890	GRV2	stress.abiotic.heat	GRV2 (GRAVITROPISM DEFECTIVE 2); binding / heat shock protein binding
AT2G36850	ATGSL08	minor CHO metabolism.callose	GSL8 (GLUCAN SYNTHASE-LIKE 8); 1,3-beta-glucan synthase/ transferase, transferring glycosyl groups
AT4G20410	GAMMA-SNAP	protein.targeting.secretory pathway.unspecified	GSNAP (GAMMA-SOLUBLE NSF ATTACHMENT PROTEIN); binding / transporter
AT4G21150	HAP6	development.unspecified	HAP6 (HAPLESS 6); dolichyl-diphosphooligosaccharide-protein glycotransferase
AT5G26040	HDA2	RNA.regulation of transcription.HDA	HDA2; histone deacetylase
AT5G14220	HEMG2	tetrapyrrole synthesis.protoporphyrin IX oxidase	HEMG2; electron carrier/ oxidoreductase/ protoporphyrinogen oxidase
AT3G23990	HSP60	protein.folding	HSP60 (HEAT SHOCK PROTEIN 60); ATP binding

AT2G33210	HSP60-2	protein.folding	HSP60-2 (HEAT SHOCK PROTEIN 60-2); ATP binding
AT5G27670	HTA7	DNA.synthesis/chromatin structure.histone.core.H2A	HTA7 (HISTONE H2A 7); DNA binding
AT1G70690	HWI1	signalling.receptor kinases.misc	HWI1 (HOPW1-1-INDUCED GENE1)
AT4G29130	ATHXK1	major CHO metabolism.degradation.sucrose.hexokinase	HXK1 (HEXOKINASE 1); ATP binding / fructokinase/ glucokinase/ hexokinase
AT2G19860	ATHXK2	major CHO metabolism.degradation.sucrose.hexokinase	HXK2 (HEXOKINASE 2); ATP binding / fructokinase/ glucokinase/ hexokinase
AT1G20050	HYD1	hormone metabolism.brassinosteroid.synthesis-degradation.sterols.HYD1	HYD1 (HYDRA1); C-8 sterol isomerase
AT1G34120	AT5P1	minor CHO metabolism.myo-inositol.poly-phosphatases	IP5PI (INOSITOL POLYPHOSPHATE 5-PHOSPHATASE I); inositol trisphosphate phosphatase/ inositol-polyphosphate 5-phosphatase
AT4G00630	ATKEA2	transport.potassium	KEA2; potassium ion transmembrane transporter/ potassium:hydrogen antiporter
AT3G05970	ATLACS6	lipid metabolism.FA synthesis and FA elongation.long chain fatty acid CoA ligase	LACS6 (long-chain acyl-CoA synthetase 6); long-chain-fatty-acid-CoA ligase
AT5G27600	ATLACS7	lipid metabolism.FA synthesis and FA elongation.long chain fatty acid CoA ligase	LACS7 (LONG-CHAIN ACYL-COA SYNTHETASE 7); long-chain-fatty-acid-CoA ligase/ protein binding
AT1G77590	LACS9	lipid metabolism.FA synthesis and FA elongation.long chain fatty acid CoA ligase	LACS9 (LONG CHAIN ACYL-COA SYNTHETASE 9); long-chain-fatty-acid-CoA ligase
AT2G37860	LCD1	development.unspecified	LCD1 (LOWER CELL DENSITY 1)
AT5G40780	LHT1	transport.amino acids	LHT1; amino acid transmembrane transporter
AT3G57650	LPAT2	lipid metabolism.Phospholipid synthesis.1-acylglycerol-3-phosphate O-acyltransferase	LPAT2; 1-acylglycerol-3-phosphate O-acyltransferase
AT3G16950	LPD1	TCA / org transformation.TCA.pyruvate DH.E3	LPD1 (LIPOAMIDE DEHYDROGENASE 1); dihydrolipoyl dehydrogenase
AT5G16590	LRR1	signalling.receptor kinases.leucine rich repeat III	LRR1; ATP binding / kinase/ protein serine/threonine kinase
AT3G25860	LTA2	lipid metabolism.FA synthesis and FA elongation.pyruvate DH	LTA2; dihydrolipoyllysine-residue acetyltransferase
AT3G52200	LTA3	TCA / org transformation.TCA.pyruvate DH.E2	LTA3; ATP binding / dihydrolipoyllysine-residue acetyltransferase

AT1G03090	MCCA	amino acid metabolism.degradation.branched chain group.leucine	MCCA; methylcrotonoyl-CoA carboxylase
AT4G34030	MCCB	amino acid metabolism.degradation.branched chain group.leucine	MCCB (3-METHYLCROTONYL-COA CARBOXYLASE); biotin carboxylase/ methylcrotonoyl-CoA carboxylase
AT3G27820	ATMDAR4	redox.ascorbate and glutathione.ascorbate	MDAR4 (MONODEHYDROASCORBATE REDUCTASE 4); monodehydroascorbate reductase (NADH)
AT1G04630	MEE4	not assigned.unknown	MEE4 (maternal effect embryo arrest 4)
AT5G27540	emb2473	signalling.G-proteins	MIRO1 (Miro-related GTP-ase 1); GTP binding
AT3G63150	ATCBG	signalling.G-proteins	MIRO2 (MIRO-RELATED GTP-ASE 2); GTPase/ calcium ion binding
ATMG00220	COB	protein.synthesis.ribosomal protein.prokaryotic.mitochondrion.30S subunit.S14	Mitochondrial apocytochrome b (cob) gene encodes a subunit of the ubiquinol-cytochrome c oxidoreductase and is part of a 5 kb transcript. The transcript also contains a pseudogene for ribosomal protein S14 called RPS15 and a tRNA(Ser) gene. Both the Cob and RPS15 genes are edited in the transcript.
AT2G42210	ATOEP16-3	protein.targeting.mitochondria	mitochondrial import inner membrane translocase subunit Tim17/Tim22/Tim23 family protein
AT3G02090	MPPBETA	protein.targeting.mitochondria	mitochondrial processing peptidase beta subunit, putative
AT3G16480	MPPalpha	protein.targeting.mitochondria	MPPalpha (mitochondrial processing peptidase alpha subunit); catalytic/ metal ion binding / metalloendopeptidase/ zinc ion binding
AT1G30400	ABCC1	transport.ABC transporters and multidrug resistance systems	MRP1 (ARABIDOPSIS THALIANA MULTIDRUG RESISTANCE-ASSOCIATED PROTEIN 1); ATPase, coupled to transmembrane movement of substances / xenobiotic-transporting ATPase
AT5G52240	AtMAPR5	redox.ascorbate and glutathione	MSBP1 (membrane steroid binding protein 1); steroid binding
AT5G19520	ATMSL9	RNA.regulation of transcription.putative transcription regulator	MSL9 (MECHANOSENSITIVE CHANNEL OF SMALL CONDUCTANCE-LIKE 9); mechanically-gated ion channel

ATMG00270	NAD6	mitochondrial electron transport / ATP synthesis.NADH-DH.localisation not clear	NADH dehydrogenase subunit 6
ATMG00510	NAD7	mitochondrial electron transport / ATP synthesis.NADH-DH.localisation not clear	NADH dehydrogenase subunit 7
ATMG00070	NAD9	mitochondrial electron transport / ATP synthesis.NADH-DH.localisation not clear	NADH dehydrogenase subunit 9
AT4G28220	NDB1	mitochondrial electron transport / ATP synthesis.NADH-DH.type II.external	NDB1 (NAD(P)H dehydrogenase B1); NADH dehydrogenase/ disulfide oxidoreductase
AT5G06320	NHL3	stress.biotic	NHL3
AT5G27150	AT-NHX1	transport.unspecified cations	NHX1 (NA+/H+ EXCHANGER); protein binding / sodium ion transmembrane transporter/ sodium:hydrogen antiporter
AT3G44320	AtNIT3	misc.nitrilases, *nitrile lyases, berberine bridge enzymes, reticuline oxidases, troponine reductases	NIT3 (NITRILASE 3); indole-3-acetonitrile nitrilase/ indole-3-acetonitrile nitrile hydratase/ nitrilase
AT1G56110	NOP56	RNA.regulation of transcription.putative transcription regulator	NOP56 (Arabidopsis homolog of nucleolar protein Nop56)
AT1G12110	ATNRT1	transport.nitrate	NRT1.1; nitrate transmembrane transporter/ transporter
AT4G04910	NSF	protein.degradation.AAA type	NSF (N-ethylmaleimide sensitive factor); ATP binding / binding / nucleoside-triphosphatase/ nucleotide binding
AT1G05500	ATSYTE	not assigned.no ontology.C2 domain-containing protein	NTMC2T2.1
AT1G80300	ATNTT1	transport.misc	NTT1 (NUCLEOTIDE TRANSPORTER 1); ATP:ADP antiporter
AT2G43950	ATOEP37	not assigned.unknown	OEP37; ion channel
AT5G19620	ATOEP80	protein.targeting.chloroplast	OEP80 (OUTER ENVELOPE PROTEIN OF 80 KDA)
AT5G62050	ATOXA1	transport.peptides and oligopeptides	OXA1; P-P-bond-hydrolysis-driven protein transmembrane transporter
AT5G35590	PAA1	protein.degradation.ubiquitin.proteasom	PAA1 (PROTEASOME ALPHA SUBUNIT A 1); endopeptidase/ peptidase/ threonine-type endopeptidase
AT2G05840	PAA2	protein.degradation.ubiquitin.proteasom	PAA2 (20S PROTEASOME SUBUNIT PAA2); endopeptidase/ peptidase/ threonine-type endopeptidase

AT1G16470	PAB1	protein.degradation	PAB1 (PROTEASOME SUBUNIT PAB1); endopeptidase/ peptidase/ threonine-type endopeptidase
AT1G49760	PAB8	RNA.processing	PAB8 (POLY(A) BINDING PROTEIN 8); RNA binding / translation initiation factor
AT3G22110	PAC1	protein.degradation.ubiquitin.proteasom	PAC1; endopeptidase/ peptidase/ threonine-type endopeptidase
AT5G42790	ARS5	protein.degradation.ubiquitin.proteasom	PAF1; endopeptidase/ peptidase/ threonine-type endopeptidase
AT4G37070	AtPLAIVA	development.storage proteins	patatin, putative
AT1G72150	PATL1	transport.misc	PATL1 (PATELLIN 1); transporter
AT1G22530	PATL2	transport.misc	PATL2 (PATELLIN 2); transporter
AT4G31300	PBA1	protein.degradation.ubiquitin.proteasom	PBA1; endopeptidase/ peptidase/ threonine-type endopeptidase
AT3G27430	PBB1	protein.degradation.ubiquitin.proteasom	PBB1; endopeptidase/ peptidase/ threonine-type endopeptidase
AT1G13060	PBE1	protein.degradation.ubiquitin.proteasom	PBE1; endopeptidase/ peptidase/ threonine-type endopeptidase
AT3G60820	PBF1	protein.degradation.ubiquitin.proteasom	PBF1; peptidase/ threonine-type endopeptidase
AT1G56450	PBG1	protein.degradation.ubiquitin.proteasom	PBG1; peptidase/ threonine-type endopeptidase
AT2G26510	PDE135	transport.misc	PDE135 (pigment defective embryo 135); transmembrane transporter
AT2G36380	ABCG34	transport.ABC transporters and multidrug resistance systems	PDR6; ATPase, coupled to transmembrane movement of substances
AT1G15210	ABCG35	transport.ABC transporters and multidrug resistance systems	PDR7 (PLEIOTROPIC DRUG RESISTANCE 7); ATPase, coupled to transmembrane movement of substances
AT3G53480	ABCG37	transport.ABC transporters and multidrug resistance systems	PDR9 (PLEIOTROPIC DRUG RESISTANCE 9); ATPase, coupled to transmembrane movement of substances
AT2G38670	PECT1	cell.division	PECT1 (PHOSPHORYLETHANOLAMINE CYTIDYLYLTRANSFERASE 1); ethanolamine-phosphate cytidylyltransferase
AT1G59870	ABCG36	transport.ABC transporters and multidrug resistance systems	PEN3 (PENETRATION 3); ATPase, coupled to transmembrane movement of substances / cadmium ion transmembrane transporter
AT5G09978	PEP7	stress.biotic.signalling	PEP7 (ELICITOR PEPTIDE 7 PRECURSOR)

AT3G61070	PEX11E	cell.organisation	PEX11E
AT5G56630	PFK7	glycolysis.unclear/dually targeted.phosphofructokinase (PFK)	PFK7 (PHOSPHOFRUCTOKINASE 7); 6-phosphofructokinase
AT1G02520	ABCB11	transport.ABC transporters and multidrug resistance systems	PGP11 (P-GLYCOPROTEIN 11); ATPase, coupled to transmembrane movement of substances
AT3G55320	ABCB20	transport.ABC transporters and multidrug resistance systems	PGP20 (P-GLYCOPROTEIN 20); ATPase, coupled to transmembrane movement of substances
AT3G62150	ABCB21	transport.ABC transporters and multidrug resistance systems	PGP21 (P-GLYCOPROTEIN 21); ATPase, coupled to transmembrane movement of substances
AT5G18480	PGSIP6	major CHO metabolism.synthesis.starch	PGSIP6 (PLANT GLYCOGENIN-LIKE STARCH INITIATION PROTEIN 6); transferase, transferring glycosyl groups / transferase, transferring hexosyl groups
AT1G33140	PGY2	protein.synthesis.ribosomal protein.eukaryotic.60S subunit.L9	PGY2 (PIGGYBACK2); structural constituent of ribosome
AT3G52190	AtPHF1	development.unspecified	PHF1 (PHOSPHATE TRANSPORTER TRAFFIC FACILITATOR1); nucleotide binding
AT4G23400	PIP1;5	transport.Major Intrinsic Proteins.PIP	PIP1;5 (PLASMA MEMBRANE INTRINSIC PROTEIN 1;5); water channel
AT3G61430	ATPIP1	transport.Major Intrinsic Proteins.PIP	PIP1A (PLASMA MEMBRANE INTRINSIC PROTEIN 1A); water channel
AT2G45960	ATHH2	transport.Major Intrinsic Proteins.PIP	PIP1B (NAMED PLASMA MEMBRANE INTRINSIC PROTEIN 1B); water channel
AT5G60660	PIP2;4	transport.Major Intrinsic Proteins.PIP	PIP2;4 (PLASMA MEMBRANE INTRINSIC PROTEIN 2;4); water channel
AT2G37170	PIP2;2	transport.Major Intrinsic Proteins.PIP	PIP2B (PLASMA MEMBRANE INTRINSIC PROTEIN 2); water channel
AT4G35100	PIP2;7	transport.Major Intrinsic Proteins.PIP	PIP3 (PLASMA MEMBRANE INTRINSIC PROTEIN 3); water channel
AT3G05630	PDLZ2	lipid metabolism.lipid degradation.lysophospholipases.phospholipase D	PLDP2; phospholipase D
AT4G04470	PMP22	protein.targeting.peroxisomes	PMP22
AT3G56110	PRA1.B1	cell.vesicle transport	PRA1.B1 (PRENYLATED RAB ACCEPTOR 1.B1)
AT3G13710	PRA1.F4	signalling.G-proteins	PRA1.F4 (PRENYLATED RAB ACCEPTOR 1.F4)

AT1G55190	PRA1.F2	cell.vesicle transport	PRA7
AT2G22000	PROPEP6	stress.biotic.signalling	PROPEP6 (Elicitor peptide 6 precursor)
AT4G25970	PSD3	lipid metabolism.Phospholipid synthesis.phosphatidylserine decarboxylase	PSD3 (phosphatidylserine decarboxylase 3); phosphatidylserine decarboxylase
AT1G65260	PTAC4	RNA.regulation of transcription.unclassified	PTAC4 (PLASTID TRANSCRIPTIONALLY ACTIVE4)
AT3G54110	ATPUMP1	mitochondrial electron transport / ATP synthesis.uncoupling protein	PUMP1 (PLANT UNCOUPLING MITOCHONDRIAL PROTEIN 1); binding / oxidative phosphorylation uncoupler
AT2G45140	PVA12	protein.targeting.secretory pathway.ER	PVA12 (PLANT VAP HOMOLOG 12); structural molecule
AT4G39850	ABCD1	transport.ABC transporters and multidrug resistance systems	PXA1 (PEROXISOMAL ABC TRANSPORTER 1); ATPase, coupled to transmembrane movement of substances
AT5G23300	PYRD	nucleotide metabolism.synthesis.pyrimidine.dihydroorotate dehydrogenase	PYRD (pyrimidine d); dihydroorotate dehydrogenase
AT2G44610	ATRAB6A	signalling.G-proteins	RAB6A; GTP binding / protein binding
AT4G39990	ATGB3	signalling.G-proteins	RABA4B (RAB GTPASE HOMOLOG A4B); GTP binding
AT4G17170	AT-RAB2	signalling.G-proteins	RABB1C (ARABIDOPSIS RAB GTPASE HOMOLOG B1C); GTP binding / GTPase
AT5G44790	HMA7	transport.metal	RAN1 (RESPONSIVE-TO-ANTAGONIST 1); ATPase, coupled to transmembrane movement of ions, phosphorylative mechanism / copper ion transmembrane transporter
AT5G47910	ATRBOHD	stress.biotic.respiratory burst	RBOHD (RESPIRATORY BURST OXIDASE HOMOLOGUE D); NAD(P)H oxidase
AT1G25490	ATB BETA BETA	protein.postranslational modification	RCN1 (ROOTS CURL IN NPA); protein phosphatase type 2A regulator
AT4G39090	RD19	protein.degradation.cysteine protease	RD19 (RESPONSIVE TO DEHYDRATION 19); cysteine-type endopeptidase/ cysteine-type peptidase
AT2G37180	PIP2;3	transport.Major Intrinsic Proteins.PIP	RD28 (RESPONSIVE TO DESICCATION 28); water channel
AT3G02230	ATRGP1	cell wall.cell wall proteins.RGP	RGP1 (REVERSIBLY GLYCOSYLATED POLYPEPTIDE 1); cellulose synthase (UDP-forming)
AT5G15650	ATRGP2	cell wall.cell wall proteins.RGP	RGP2 (REVERSIBLY GLYCOSYLATED POLYPEPTIDE 2);

			transferase, transferring hexosyl groups
AT3G13870	GOM8	development.unspecified	RHD3 (ROOT HAIR DEFECTIVE 3); GTP binding
AT3G51460	RHD4	lipid metabolism.lipid degradation.lysophospholipases.phosphoinositide phospholipase C	RHD4 (ROOT HAIR DEFECTIVE4); phosphatidylinositol-4,5-bisphosphate 5-phosphatase/ phosphatidylinositol-4-phosphate phosphatase
AT3G05590	RPL18	protein.synthesis.ribosomal protein.eukaryotic.60S subunit.L18	RPL18 (RIBOSOMAL PROTEIN L18); structural constituent of ribosome
AT1G69620	RPL34	protein.synthesis.ribosomal protein.eukaryotic.60S subunit.L34	RPL34 (RIBOSOMAL PROTEIN L34); structural constituent of ribosome
AT4G38630	ATMCB1	protein.degradation.ubiquitin	RPN10 (REGULATORY PARTICLE NON-ATPASE 10); peptide receptor
AT1G64520	RPN12a	protein.degradation.ubiquitin.proteasom	RPN12a (Regulatory Particle non-ATPase 12a); peptidase
AT2G20580	ATRPN1A	protein.degradation.ubiquitin.proteasom	RPN1A (26S PROTEASOME REGULATORY SUBUNIT S2 1A); binding / enzyme regulator
AT5G05780	AE3	protein.degradation.ubiquitin.proteasom	RPN8A (RP NON-ATPASE SUBUNIT 8A)
AT5G23740	RPS11-BETA	protein.synthesis.ribosomal protein.eukaryotic.40S subunit.S11	RPS11-BETA (RIBOSOMAL PROTEIN S11-BETA); structural constituent of ribosome
AT1G04270	RPS15	protein.synthesis.ribosomal protein.eukaryotic.40S subunit.S15	RPS15 (CYTOSOLIC RIBOSOMAL PROTEIN S15); structural constituent of ribosome
AT3G46040	RPS15AD	protein.synthesis.ribosomal protein.eukaryotic.40S subunit.S15A	RPS15AD (ribosomal protein S15A D); structural constituent of ribosome
AT4G09800	RPS18C	protein.synthesis.ribosomal protein.eukaryotic.40S subunit.S18	RPS18C (S18 RIBOSOMAL PROTEIN); RNA binding / nucleic acid binding / structural constituent of ribosome
AT1G53750	RPT1A	protein.degradation.ubiquitin.proteasom	RPT1A (REGULATORY PARTICLE TRIPLE-A 1A); ATPase
AT5G58290	RPT3	protein.degradation.ubiquitin.proteasom	RPT3 (REGULATORY PARTICLE TRIPLE-A ATPASE 3); ATPase
AT5G43010	RPT4A	protein.degradation.ubiquitin.proteasom	RPT4A; ATPase
AT3G05530	ATS6A.2	protein.degradation.ubiquitin.proteasom	RPT5A (REGULATORY PARTICLE TRIPLE-A ATPASE 5A); ATPase/ calmodulin binding
AT5G19990	ATSUG1	protein.degradation.ubiquitin.proteasom	RPT6A (REGULATORY PARTICLE TRIPLE-A ATPASE 6A); ATPase
AT2G33120	ATVAMP722	cell.vesicle transport	SAR1 (SYNAPTOBREVIN-RELATED PROTEIN 1)

AT1G20780	ATPUB44	stress.biotic	SAUL1 (SENESCENCE-ASSOCIATED E3 UBIQUITIN LIGASE 1); ubiquitin-protein ligase
AT1G61250	SC3	transport.misc	SC3 (SECRETORY CARRIER 3); transmembrane transporter
AT3G10410	CPY	protein.degradation.serine protease	scpl49 (serine carboxypeptidase-like 49); serine-type carboxypeptidase
AT3G10370	SDP6	lipid metabolism.glyceral metabolism.FAD-dependent glycerol-3-phosphate dehydrogenase	SDP6 (SUGAR-DEPENDENT 6); glycerol-3-phosphate dehydrogenase
AT5G12370	SEC10	cell.vesicle transport	SEC10 (EXOCYST COMPLEX COMPONENT SEC10)
AT1G11890	ATSEC22	protein.targeting.secretory pathway.unspecified	SEC22; transporter
AT1G71820	SEC6	not assigned.unknown	SEC6
AT2G45070	SEC61 BETA	protein.targeting.secretory pathway.unspecified	SEC61 BETA; protein transporter
AT3G10380	ATSEC8	cell.vesicle transport	SEC8 (SUBUNIT OF EXOCYST COMPLEX 8)
AT4G32400	ATBT1	transport.metabolite transporters at the envelope membrane	SHS1 (SODIUM HYPERSENSITIVE 1); binding / nucleotide transmembrane transporter/ transporter
AT5G13710	CPH	hormone metabolism.brassinosteroid.synthesis-degradation.sterols.SMT2	SMT1 (STEROL METHYLTRANSFERASE 1); sterol 24-C-methyltransferase
AT1G20330	CVP1	hormone metabolism.brassinosteroid.synthesis-degradation.sterols.SMT2	SMT2 (STEROL METHYLTRANSFERASE 2); S-adenosylmethionine-dependent methyltransferase
AT1G76090	SMT3	hormone metabolism.brassinosteroid.synthesis-degradation.sterols.SMT2	SMT3 (STEROL METHYLTRANSFERASE 3); S-adenosylmethionine-dependent methyltransferase/ sterol 24-C-methyltransferase
AT5G06140	ATSNX1	protein.targeting.secretory pathway.vacuole	SNX1 (SORTING NEXIN 1); phosphoinositide binding / protein binding
AT5G58440	SNX2a	cell.vesicle transport	SNX2a (SORTING NEXIN 2a); phosphoinositide binding
AT2G37970	SOUL-1	not assigned.no ontology	SOUL-1; binding
AT4G21540	SPHK1	lipid metabolism.'exotics' (steroids, squalene etc).sphingolipids	SPHK1 (SPHINGOSINE KINASE 1); D-erythro-sphingosine kinase/ diacylglycerol kinase/ sphinganine kinase
AT5G01220	SQD2	lipid metabolism.glycolipid synthesis.sulfolipid synthase	SQD2 (sulfoquinovosyldiacylglycerol 2); UDP-glycosyltransferase/ UDP-

			sulfoquinovose:DAG sulfoquinovosyltransferase/ transferase, transferring glycosyl groups
AT5G24140	SQP2	hormone metabolism.brassinosteroid.synthesis-degradation.sterols.other	SQP2; FAD binding / oxidoreductase/ squalene monooxygenase
AT4G34640	ERG9	lipid metabolism.'exotics' (steroids, squalene etc).squalene synthase	SQS1 (SQUALENE SYNTHASE 1); farnesyl-diphosphate farnesyltransferase
AT4G22130	SRF8	protein.postranslational modification.kinase.receptor like cytoplasmatic kinase VII	SRF8 (STRUBBELIG-RECEPTOR FAMILY 8); ATP binding / kinase/ protein binding / protein kinase/ protein serine/threonine kinase/ protein tyrosine kinase
AT1G74020	SS2	secondary metabolism.N misc.alkaloid-like	SS2 (STRICTOSIDINE SYNTHASE 2); strictosidine synthase
AT5G58270	ABCB25	transport.ABC transporters and multidrug resistance systems	STA1 (STARIK 1); ATPase, coupled to transmembrane movement of substances / transporter
AT2G01470	ATSEC12	protein.degradation	STL2P (SEC12P-LIKE 2 PROTEIN); nucleotide binding
AT1G11260	ATSTP1	transport.sugars	STP1 (SUGAR TRANSPORTER 1); carbohydrate transmembrane transporter/ sugar:hydrogen symporter
AT3G19930	ATSTP4	transport.sugars	STP4 (SUGAR TRANSPORTER 4); carbohydrate transmembrane transporter/ monosaccharide transmembrane transporter/ sucrose:hydrogen symporter/ sugar:hydrogen symporter
AT5G19690	STT3A	stress.abiotic.drought/salt	STT3A (STAUROSPORIN AND TEMPERATURE SENSITIVE 3-LIKE A); oligosaccharyl transferase
AT1G34130	STT3B	stress.abiotic.drought/salt	STT3B (staurosporin and temperature sensitive 3-like b); oligosaccharyl transferase
AT3G17910	EMB3121	mitochondrial electron transport / ATP synthesis.cytochrome c oxidase	SURF1 (SURFEIT 1)
AT1G80070	EMB14	RNA.processing.splicing	SUS2 (ABNORMAL SUSPENSOR 2)
AT3G11820	AT-SYR1	cell.vesicle transport	SYP121 (SYNTAXIN OF PLANTS 121); SNAP receptor/ protein anchor
AT3G52400	ATSYP122	cell.vesicle transport	SYP122 (SYNTAXIN OF PLANTS 122); SNAP receptor
AT5G08080	ATSYP132	cell.vesicle transport	SYP132 (SYNTAXIN OF PLANTS 132); SNAP receptor
AT3G05710	ATSYP43	cell.vesicle transport	SYP43 (SYNTAXIN OF PLANTS 43); SNAP receptor

AT3G09740	ATSYP71	cell.vesicle transport	SYP71 (SYNTAXIN OF PLANTS 71); protein transporter
AT2G20990	ATSYTA	not assigned.no ontology.C2 domain-containing protein	SYTA (SYNAPTOTAGMIN A)
AT3G45600	TET3	development.unspecified	TET3 (TETRASPANIN3)
AT2G23810	TET8	development.unspecified	TET8 (TETRASPANIN8)
AT5G48010	AtTHAS1	secondary metabolism.isoprenoids.terpenoids	THAS1 (THALIANOL SYNTHASE 1); catalytic/ thalianol synthase
AT4G27080	ATPDI7	redox.thioredoxin.PDIL	thioredoxin family protein
AT1G06950	ATTIC110	protein.targeting.chloroplast	TIC110 (TRANSLOCON AT THE INNER ENVELOPE MEMBRANE OF CHLOROPLASTS 110)
AT1G10840	TIF3H1	protein.synthesis.initiation	TIF3H1; translation initiation factor
AT5G58070	ATTIL	transport.misc	TIL (TEMPERATURE-INDUCED LIPOCALIN); binding / transporter
AT1G55900	EMB1860	transport.metabolite transporters at the mitochondrial membrane	TIM50
AT5G20350	TIP1	cell.organisation	TIP1 (TIP GROWTH DEFECTIVE 1); S-acyltransferase/ acyl binding
AT3G26520	GAMMA-TIP2	transport.Major Intrinsic Proteins.TIP	TIP2 (TONOPLAST INTRINSIC PROTEIN 2); water channel
AT1G45201	ATTLL1	lipid metabolism.lipid degradation.lipases.triacylglycerol lipase	TLL1 (TRIACYLGLYCEROL LIPASE-LIKE 1); triacylglycerol lipase
AT1G20840	AtTMT1	transport.sugars	TMT1 (TONOPLAST MONOSACCHARIDE TRANSPORTER1); carbohydrate transmembrane transporter/ nucleoside transmembrane transporter/ sugar:hydrogen symporter
AT4G35300	TMT2	transport.sugars	TMT2 (TONOPLAST MONOSACCHARIDE TRANSPORTER2); carbohydrate transmembrane transporter/ nucleoside transmembrane transporter/ sugar:hydrogen symporter
AT5G05000	ATTOC34	protein.targeting.chloroplast	TOC34 (TRANSLOCON AT THE OUTER ENVELOPE MEMBRANE OF CHLOROPLASTS 34); GTP binding
AT3G46740	MAR1	protein.targeting.chloroplast	TOC75-III (TRANSLOCON AT THE OUTER ENVELOPE MEMBRANE OF CHLOROPLASTS 75-III); P-P-bond-hydrolysis-driven protein transmembrane transporter

AT1G27390	TOM20-2	protein.targeting.mitochondria	TOM20-2 (TRANSLOCASE OUTER MEMBRANE 20-2); P-P-bond-hydrolysis-driven protein transmembrane transporter/ metal ion binding
AT3G27080	TOM20-3	protein.targeting.mitochondria	TOM20-3 (TRANSLOCASE OF OUTER MEMBRANE 20 KDA SUBUNIT 3); P-P-bond-hydrolysis-driven protein transmembrane transporter
AT5G40930	TOM20-4	protein.targeting.mitochondria	TOM20-4 (TRANSLOCASE OF OUTER MEMBRANE 20-4); P-P-bond-hydrolysis-driven protein transmembrane transporter
AT5G43970	ATTOM22-V	protein.targeting.mitochondria	TOM22-V (TRANSLOCASE OF OUTER MEMBRANE 22-V); P-P-bond-hydrolysis-driven protein transmembrane transporter
AT1G32400	TOM2A	development.unspecified	TOM2A (TOBAMOVIRUS MULTIPLICATION 2A); protein binding
AT3G20000	TOM40	protein.targeting.mitochondria	TOM40; P-P-bond-hydrolysis-driven protein transmembrane transporter/ voltage-gated anion channel
AT5G08040	TOM5	protein.targeting.unknown	TOM5 (MITOCHONDRIAL IMPORT RECEPTOR SUBUNIT TOM5 HOMOLOG)
AT4G20850	TPP2	protein.degradation.serine protease	TPP2 (TRIPEPTIDYL PEPTIDASE II); tripeptidyl-peptidase
AT4G23640	ATKT3	transport.potassium	TRH1 (TINY ROOT HAIR 1); potassium ion transmembrane transporter
AT5G19780	TUA5	cell.organisation	TUA5; structural constituent of cytoskeleton
AT5G44340	TUB4	cell.organisation	TUB4; structural constituent of cytoskeleton
AT1G20010	TUB5	cell.organisation	TUB5; structural constituent of cytoskeleton
AT4G11150	emb2448	transport.p- and v-ATPases.H+-transporting two-sector ATPase.subunit E	TUF (VACUOLAR ATP SYNTHASE SUBUNIT E1); proton-transporting ATPase, rotational mechanism
AT3G53520	ATUXS1	cell wall.precursor synthesis.UXS	UXS1 (UDP-GLUCURONIC ACID DECARBOXYLASE 1); UDP-glucuronate decarboxylase/ catalytic
AT3G60600	(AT)VAP	DNA.unspecified	VAP (VESICLE ASSOCIATED PROTEIN); protein binding
AT5G42270	FTSH5	protein.degradation.metalloprotease	VAR1 (VARIEGATED 1); ATP-dependent peptidase/ ATPase/ metallopeptidase

AT2G30950	FTSH2	protein.degradation.metalloprotease	VAR2 (VARIEGATED 2); ATP-dependent peptidase/ ATPase/ metallopeptidase/ zinc ion binding
AT3G01280	ATVDAC1	transport.porins	VDAC1 (VOLTAGE DEPENDENT ANION CHANNEL 1); voltage-gated anion channel
AT5G67500	ATVDAC2	transport.porins	VDAC2 (VOLTAGE DEPENDENT ANION CHANNEL 2); voltage-gated anion channel
AT5G15090	ATVDAC3	transport.porins	VDAC3 (VOLTAGE DEPENDENT ANION CHANNEL 3); voltage-gated anion channel
AT5G57490	ATVDAC4	transport.unspecified anions	VDAC4 (VOLTAGE DEPENDENT ANION CHANNEL 4); voltage-gated anion channel
AT1G78900	VHA-A	transport.p- and v-ATPases	VHA-A (VACUOLAR ATP SYNTHASE SUBUNIT A); ATP binding / hydrogen ion transporting ATP synthase, rotational mechanism / hydrolase, acting on acid anhydrides, catalyzing transmembrane movement of substances / proton-transporting ATPase, rotational mechanism
AT2G28520	VHA-A1	transport.p- and v-ATPases	VHA-A1 (VACUOLAR PROTON ATPASE A 1); ATPase
AT2G21410	VHA-A2	transport.p- and v-ATPases	VHA-A2 (VACUOLAR PROTON ATPASE A2); ATPase
AT4G39080	VHA-A3	transport.p- and v-ATPases	VHA-A3 (VACUOLAR PROTON ATPASE A3); ATPase
AT1G64200	VHA-E3	transport.p- and v-ATPases.H+-transporting two-sector ATPase	VHA-E3 (VACUOLAR H+-ATPASE SUBUNIT E ISOFORM 3); proton-transporting ATPase, rotational mechanism
AT3G01390	AVMA10	transport.p- and v-ATPases	VMA10 (VACUOLAR MEMBRANE ATPASE 10); hydrogen ion transporting ATP synthase, rotational mechanism
AT5G63880	VPS20.1	RNA.regulation of transcription.SNF7	VPS20.1
AT5G09260	VPS20.2	cell.vesicle transport	VPS20.2 (VACUOLAR PROTEIN SORTING-ASSOCIATED PROTEIN 20.2)
AT1G77140	ATVPS45	protein.targeting.secretory pathway.vacuole	VPS45 (VACUOLAR PROTEIN SORTING 45); protein transporter
AT3G52850	ATELP	protein.targeting.secretory pathway.vacuole	VSR1 (VACUOLAR SORTING RECEPTOR HOMOLOG 1); amino-terminal vacuolar sorting propeptide binding
AT2G14720	BP80-2;1	protein.targeting.secretory pathway.vacuole	VSR-2; calcium ion binding

AT1G09850	XBCP3	protein.degradation.cysteine protease	XBCP3 (xylem bark cysteine peptidase 3); cysteine-type endopeptidase/ cysteine-type peptidase
AT5G20490	ATXIK	cell.organisation	XIK; motor/ protein binding
AT5G55500	ATXYLT	protein.glycosylation.beta-1,2-xylosyltransferase(beta-1,2-XylT)	XYLT (ARABIDOPSIS THALIANA BETA-1,2-XYLOSYLTRANSFERASE); xylosyltransferase
AT1G67730	ATKCR1	secondary metabolism.wax	YBR159; ketoreductase/ oxidoreductase
AT3G51430	SSL5	secondary metabolism.N misc.alkaloid-like	YLS2; strictosidine synthase
AT3G47630		not assigned.unknown	FUNCTIONS IN: molecular_function unknown; INVOLVED IN: biological_process unknown;
AT4G17140		not assigned.no ontology.C2 domain-containing protein	FUNCTIONS IN: molecular_function unknown; INVOLVED IN: biological_process unknown;
AT3G55600		not assigned.unknown	FUNCTIONS IN: molecular_function unknown; INVOLVED IN: biological_process unknown;
AT5G62270		not assigned.unknown	FUNCTIONS IN: molecular_function unknown; INVOLVED IN: biological_process unknown; LOCATED IN: cellular_component unknown
AT3G48380		not assigned.unknown	FUNCTIONS IN: molecular_function unknown; INVOLVED IN: biological_process unknown; LOCATED IN: cellular_component unknown
AT5G40510		not assigned.unknown	FUNCTIONS IN: molecular_function unknown; INVOLVED IN: biological_process unknown; LOCATED IN: cellular_component unknown
AT4G12340		not assigned.unknown	FUNCTIONS IN: molecular_function unknown; INVOLVED IN: biological_process unknown; LOCATED IN: cellular_component unknown
AT5G10780		not assigned.no ontology	FUNCTIONS IN: molecular_function unknown; INVOLVED IN: biological_process unknown; LOCATED IN: cellular_component unknown
AT4G26410		not assigned.unknown	FUNCTIONS IN: molecular_function unknown; INVOLVED IN: biological_process unknown; LOCATED IN: cellular_component unknown

AT4G00026	SD3	not assigned.unknown	FUNCTIONS IN: molecular_function unknown; INVOLVED IN: biological_process unknown; LOCATED IN: chloroplast
AT2G41475		development.unspecified	FUNCTIONS IN: molecular_function unknown; INVOLVED IN: biological_process unknown; LOCATED IN: endomembrane system
AT3G27570		not assigned.unknown	FUNCTIONS IN: molecular_function unknown; INVOLVED IN: biological_process unknown; LOCATED IN: endomembrane system
AT5G47420		not assigned.unknown	FUNCTIONS IN: molecular_function unknown; INVOLVED IN: biological_process unknown; LOCATED IN: endoplasmic reticulum
AT2G35610	XEG113	not assigned.unknown	FUNCTIONS IN: molecular_function unknown; INVOLVED IN: biological_process unknown; LOCATED IN: Golgi apparatus
AT1G33230		not assigned.unknown	FUNCTIONS IN: molecular_function unknown; INVOLVED IN: biological_process unknown; LOCATED IN: integral to membrane
AT2G44640		not assigned.unknown	FUNCTIONS IN: molecular_function unknown; INVOLVED IN: biological_process unknown; LOCATED IN: mitochondrion, chloroplast, plasma membrane, plastid, chloroplast envelope
AT2G40800		not assigned.unknown	FUNCTIONS IN: molecular_function unknown; INVOLVED IN: biological_process unknown; LOCATED IN: mitochondrion
AT2G45060		not assigned.unknown	FUNCTIONS IN: molecular_function unknown; INVOLVED IN: biological_process unknown; LOCATED IN: mitochondrion
AT4G24290		not assigned.unknown	FUNCTIONS IN: molecular_function unknown; INVOLVED IN: biological_process unknown; LOCATED IN: plasma membrane
AT1G17620		not assigned.unknown	FUNCTIONS IN: molecular_function unknown; INVOLVED IN: biological_process unknown; LOCATED IN: plasma membrane
AT5G64090		not assigned.unknown	FUNCTIONS IN: molecular_function unknown; INVOLVED IN:

			biological_process unknown; LOCATED IN: plasma membrane
AT5G24710		not assigned.no ontology	FUNCTIONS IN: molecular_function unknown; INVOLVED IN: biological_process unknown; LOCATED IN: plasma membrane
AT2G31140		not assigned.unknown	FUNCTIONS IN: molecular_function unknown; INVOLVED IN: biological_process unknown; LOCATED IN: plasma membrane
AT3G23180		not assigned.no ontology	FUNCTIONS IN: molecular_function unknown; INVOLVED IN: defense response; LOCATED IN: membrane
AT5G42570		not assigned.no ontology	FUNCTIONS IN: molecular_function unknown; INVOLVED IN: intracellular protein transport; LOCATED IN: endoplasmic reticulum, plasma membrane
AT2G32240		not assigned.unknown	FUNCTIONS IN: molecular_function unknown; INVOLVED IN: response to cadmium ion; LOCATED IN: plasma membrane
AT1G14870	AtPCR2	not assigned.no ontology	FUNCTIONS IN: molecular_function unknown; INVOLVED IN: response to oxidative stress; LOCATED IN: plasma membrane
AT1G61670		not assigned.no ontology	FUNCTIONS IN: molecular_function unknown; ; LOCATED IN: endomembrane system, integral to membrane
AT4G28770		not assigned.unknown	FUNCTIONS IN: molecular_function unknown; ; LOCATED IN: plasma membrane, vacuole, membrane
AT3G51140		not assigned.unknown	; INVOLVED IN: biological_process unknown; LOCATED IN: chloroplast, chloroplast inner membrane, chloroplast envelope
AT5G24690		not assigned.unknown	; INVOLVED IN: biological_process unknown; LOCATED IN: mitochondrion, chloroplast, plastid, chloroplast inner membrane, chloroplast envelope
AT3G44330		not assigned.unknown	; INVOLVED IN: protein processing; LOCATED IN: mitochondrion, endoplasmic reticulum, plasma membrane, vacuole
AT5G66420		not assigned.unknown	; ; LOCATED IN: cellular_component unknown
AT5G42090		not assigned.unknown	; ; LOCATED IN: endomembrane system, integral to membrane
AT1G69450		stress.abiotic.drought/salt	; ; LOCATED IN: endomembrane system, membrane

AT4G29520		not assigned.unknown	; ; LOCATED IN: endoplasmic reticulum, plasma membrane
AT1G19360	RRA3	not assigned.unknown	; ; LOCATED IN: endoplasmic reticulum
AT4G12650		not assigned.unknown	; ; LOCATED IN: integral to membrane, Golgi apparatus, plasma membrane, vacuole
AT5G35160		not assigned.unknown	; ; LOCATED IN: integral to membrane, Golgi apparatus
AT5G23890		not assigned.no ontology	; ; LOCATED IN: mitochondrion, chloroplast thylakoid membrane, chloroplast, plastid, chloroplast envelope
AT3G58840	PMD1	not assigned.unknown	; ; LOCATED IN: mitochondrion, plastid
AT2G20230		not assigned.unknown	; ; LOCATED IN: plasma membrane, vacuole
AT3G19340		not assigned.unknown	; ; LOCATED IN: plasma membrane
AT5G20660		protein.degradation	24 kDa vacuolar protein, putative
AT2G20140	RPT2b	protein.degradation.ubiquitin.proteasom	26S protease regulatory complex subunit 4, putative
AT5G23540		protein.degradation.ubiquitin.proteasom	26S proteasome regulatory subunit, putative
AT1G22410		amino acid metabolism.synthesis.aromatic aa.chorismate.3-deoxy-D-arabino-heptulosonate 7-phosphate synthase	2-dehydro-3-deoxyphosphoheptonate aldolase, putative / 3-deoxy-D-arabino-heptulosonate 7-phosphate synthase, putative / DAHP synthetase, putative
AT4G26910		not assigned.no ontology	2-oxoacid dehydrogenase family protein
AT5G55070		TCA / org transformation.TCA.2-oxoglutarate dehydrogenase	2-oxoacid dehydrogenase family protein
AT2G33630		lipid metabolism.'exotics' (steroids, squalene etc).3-beta hydroxysteroid dehydrogenase/isomerase	3-beta hydroxysteroid dehydrogenase/isomerase family protein
AT2G16530		hormone metabolism.brassinosteroid.synthesis-degradation.BRs.DET2	3-oxo-5-alpha-steroid 4-dehydrogenase family protein / steroid 5-alpha-reductase family protein
AT4G25740		protein.synthesis.ribosomal protein.eukaryotic.40S subunit.S10	40S ribosomal protein S10 (RPS10A)
AT4G30800		protein.synthesis.ribosomal protein.eukaryotic.40S subunit.S11	40S ribosomal protein S11 (RPS11B)
AT1G15930		protein.synthesis.ribosomal protein.eukaryotic.40S subunit.S12	40S ribosomal protein S12 (RPS12A)
AT5G59850		protein.synthesis.ribosomal protein.eukaryotic.40S subunit.S15A	40S ribosomal protein S15A (RPS15aF)

AT2G09990		protein.synthesis.ribosomal protein.eukaryotic.40S subunit.S16	40S ribosomal protein S16 (RPS16A)
AT5G18380		protein.synthesis.ribosomal protein.eukaryotic.40S subunit.S16	40S ribosomal protein S16 (RPS16C)
AT3G02080		protein.synthesis.ribosomal protein.eukaryotic.40S subunit.S19	40S ribosomal protein S19 (RPS19A)
AT5G61170		protein.synthesis.ribosomal protein.eukaryotic.40S subunit.S19	40S ribosomal protein S19 (RPS19C)
AT2G41840		protein.synthesis.ribosomal protein.eukaryotic.40S subunit.S2	40S ribosomal protein S2 (RPS2C)
AT3G47370		protein.synthesis.ribosomal protein.eukaryotic.40S subunit.S20	40S ribosomal protein S20 (RPS20B)
AT5G62300		protein.synthesis.ribosomal protein.eukaryotic.40S subunit.S20	40S ribosomal protein S20 (RPS20C)
AT5G28060		protein.synthesis.ribosomal protein.eukaryotic.40S subunit.S24	40S ribosomal protein S24 (RPS24B)
AT2G21580		protein.synthesis.ribosomal protein.eukaryotic.40S subunit.S25	40S ribosomal protein S25 (RPS25B)
AT4G39200		protein.synthesis.ribosomal protein.eukaryotic.40S subunit.S25	40S ribosomal protein S25 (RPS25E)
AT2G40590		protein.synthesis.ribosomal protein.eukaryotic.40S subunit.S26	40S ribosomal protein S26 (RPS26B)
AT4G33865		protein.synthesis.ribosomal protein.eukaryotic.40S subunit.S29	40S ribosomal protein S29 (RPS29C)
AT2G31610		protein.synthesis.ribosomal protein.eukaryotic.40S subunit.S3	40S ribosomal protein S3 (RPS3A)
AT5G35530		protein.synthesis.ribosomal protein.eukaryotic.40S subunit.S3	40S ribosomal protein S3 (RPS3C)
AT3G04840		protein.synthesis.ribosomal protein.eukaryotic.40S subunit.S3A	40S ribosomal protein S3A (RPS3aA)
AT4G34670		protein.synthesis.ribosomal protein.eukaryotic.40S subunit.S3A	40S ribosomal protein S3A (RPS3aB)
AT5G07090		protein.synthesis.ribosomal protein.eukaryotic.40S subunit.S4	40S ribosomal protein S4 (RPS4B)
AT1G48830		protein.synthesis.ribosomal protein.eukaryotic.40S subunit.S7	40S ribosomal protein S7 (RPS7A)
AT3G02560		protein.synthesis.ribosomal protein.eukaryotic.40S subunit.S7	40S ribosomal protein S7 (RPS7B)
AT5G16130		protein.synthesis.ribosomal protein.eukaryotic.40S subunit.S7	40S ribosomal protein S7 (RPS7C)
AT5G15200		protein.synthesis.ribosomal protein.eukaryotic.40S subunit.S9	40S ribosomal protein S9 (RPS9B)
AT3G09200		protein.synthesis.ribosomal protein.eukaryotic.60S subunit.P0	60S acidic ribosomal protein P0 (RPP0B)
AT1G01100		protein.synthesis.ribosomal protein.eukaryotic.60S subunit.P1	60S acidic ribosomal protein P1 (RPP1A)
AT4G00810		protein.synthesis.ribosomal protein.eukaryotic.60S subunit.P1	60S acidic ribosomal protein P1 (RPP1B)
AT5G47700		protein.synthesis.ribosomal protein.eukaryotic.60S subunit.P1	60S acidic ribosomal protein P1 (RPP1C)
AT2G27720		protein.synthesis.ribosomal protein.eukaryotic.60S subunit.P2	60S acidic ribosomal protein P2 (RPP2A)

AT2G27710		protein.synthesis.ribosomal protein.eukaryotic.60S subunit.P2	60S acidic ribosomal protein P2 (RPP2B)
AT1G66580	RPL10C	protein.synthesis.ribosomal protein.eukaryotic.60S subunit.L10	60S ribosomal protein L10 (RPL10C)
AT5G45775		protein.synthesis.ribosomal protein.eukaryotic.60S subunit.L11	60S ribosomal protein L11 (RPL11D)
AT3G53430		protein.synthesis.ribosomal protein.eukaryotic.60S subunit.L12	60S ribosomal protein L12 (RPL12B)
AT3G07110		protein.synthesis.ribosomal protein.eukaryotic.60S subunit.L13A	60S ribosomal protein L13A (RPL13aA)
AT3G24830		protein.synthesis.ribosomal protein.eukaryotic.60S subunit.L13A	60S ribosomal protein L13A (RPL13aB)
AT2G20450		protein.synthesis.ribosomal protein.eukaryotic.60S subunit.L14	60S ribosomal protein L14 (RPL14A)
AT4G27090		protein.synthesis.ribosomal protein.eukaryotic.60S subunit.L14	60S ribosomal protein L14 (RPL14B)
AT4G16720		protein.synthesis.ribosomal protein.eukaryotic.60S subunit.L15	60S ribosomal protein L15 (RPL15A)
AT1G27400		protein.synthesis.ribosomal protein.eukaryotic.60S subunit.L17	60S ribosomal protein L17 (RPL17A)
AT1G67430		protein.synthesis.ribosomal protein.eukaryotic.60S subunit.L17	60S ribosomal protein L17 (RPL17B)
AT2G34480		protein.synthesis.ribosomal protein.eukaryotic.60S subunit.L18A	60S ribosomal protein L18A (RPL18aB)
AT3G14600		protein.synthesis.ribosomal protein.eukaryotic.60S subunit.L18A	60S ribosomal protein L18A (RPL18aC)
AT1G57860		protein.synthesis.ribosomal protein.eukaryotic.60S subunit.L21	60S ribosomal protein L21
AT5G27770		protein.synthesis.ribosomal protein.eukaryotic.60S subunit.L22	60S ribosomal protein L22 (RPL22C)
AT3G05560		protein.synthesis.ribosomal protein.eukaryotic.60S subunit.L22	60S ribosomal protein L22-2 (RPL22B)
AT3G49910		protein.synthesis.ribosomal protein.eukaryotic.60S subunit.L26	60S ribosomal protein L26 (RPL26A)
AT4G15000		protein.synthesis.ribosomal protein.eukaryotic.60S subunit.L27	60S ribosomal protein L27 (RPL27C)
AT3G18740	RLK902	protein.synthesis.ribosomal protein.eukaryotic.60S subunit.L30	60S ribosomal protein L30 (RPL30C)
AT4G26230		protein.synthesis.ribosomal protein.eukaryotic.60S subunit.L31	60S ribosomal protein L31 (RPL31B)
AT5G56710		protein.synthesis.ribosomal protein.eukaryotic.60S subunit.L31	60S ribosomal protein L31 (RPL31C)
AT1G26880		protein.synthesis.ribosomal protein.eukaryotic.60S subunit.L34	60S ribosomal protein L34 (RPL34A)
AT3G28900		protein.synthesis.ribosomal protein.eukaryotic.60S subunit.L34	60S ribosomal protein L34 (RPL34C)

AT3G55750		protein.synthesis.ribosomal protein.eukaryotic.60S subunit.L35A	60S ribosomal protein L35a (RPL35aD)
AT3G53740		protein.synthesis.ribosomal protein.eukaryotic.60S subunit.L36	60S ribosomal protein L36 (RPL36B)
AT3G10950		protein.synthesis.ribosomal protein.eukaryotic.60S subunit.L37A	60S ribosomal protein L37a (RPL37aB)
AT3G59540		protein.synthesis.ribosomal protein.eukaryotic.60S subunit.L38	60S ribosomal protein L38 (RPL38B)
AT4G31985		protein.synthesis.ribosomal protein.eukaryotic.60S subunit.L39	60S ribosomal protein L39 (RPL39C)
AT1G18540		protein.synthesis.ribosomal protein.eukaryotic.60S subunit.L6	60S ribosomal protein L6 (RPL6A)
AT2G18330		protein.degradation.AAA type	AAA-type ATPase family protein
AT2G37360	ABCG2	transport.ABC transporters and multidrug resistance systems	ABC transporter family protein
AT4G28570		not assigned.no ontology	alcohol oxidase-related
AT5G07030		RNA.regulation of transcription.putative transcription regulator	aspartic-type endopeptidase
AT1G11910	APA1	protein.degradation.aspartate protease	aspartyl protease family protein
AT3G03060		protein.degradation.ubiquitin.proteasom	ATP binding / ATPase/ nucleoside-triphosphatase/ nucleotide binding
AT1G63500		signalling.kinase.RLCK.brassinosteroid signalling kinase	ATP binding / binding / protein kinase/ protein serine/threonine kinase/ protein tyrosine kinase
AT5G56500	Cpn60beta3	protein.folding	ATP binding / protein binding
AT2G07698		mitochondrial electron transport / ATP synthesis.F1-ATPase	ATP synthase alpha chain, mitochondrial, putative
AT5G47030		mitochondrial electron transport / ATP synthesis.F1-ATPase	ATP synthase delta' chain, mitochondrial
AT5G13450	ATP5	mitochondrial electron transport / ATP synthesis.F1-ATPase	ATP synthase delta chain, mitochondrial, putative / H(+)-transporting two-sector ATPase, delta (OSCP) subunit, putative
AT1G51650		mitochondrial electron transport / ATP synthesis.F1-ATPase	ATP synthase epsilon chain, mitochondrial
AT2G33040	ATP3	mitochondrial electron transport / ATP synthesis.F1-ATPase	ATP synthase gamma chain, mitochondrial (ATPC)
AT5G23630	MIA	transport.unspecified cations	ATPase E1-E2 type family protein / haloacid dehalogenase-like hydrolase familiy protein
AT3G25290		hormone metabolism.auxin.induced-regulated-responsive-activated	auxin-responsive family protein

AT5G35735		hormone metabolism.auxin.induced-regulated-responsive-activated	auxin-responsive family protein
AT1G69840		not assigned.no ontology	band 7 family protein
AT2G03510		not assigned.no ontology	band 7 family protein
AT3G01290	AtHIR2	not assigned.no ontology	band 7 family protein
AT4G27585		not assigned.no ontology	band 7 family protein
AT5G51570		not assigned.no ontology	band 7 family protein
AT5G54100		not assigned.no ontology	band 7 family protein
AT5G62740	ATHIR1	not assigned.no ontology	band 7 family protein
AT1G64790	ILA	protein.synthesis	binding
AT2G01690		not assigned.unknown	binding
AT3G04830		not assigned.unknown	binding
AT3G56210		not assigned.unknown	binding
AT5G41950		not assigned.unknown	binding
AT5G39410		not assigned.unknown	binding / catalytic
AT2G20360		mitochondrial electron transport / ATP synthesis.NADH-DH.localisation not clear	binding / catalytic/ coenzyme binding
AT5G10730		not assigned.unknown	binding / catalytic/ coenzyme binding
AT3G03305		misc.calcineurin-like phosphoesterase family protein	calcineurin-like phosphoesterase family protein
AT1G05150		signalling.calcium	calcium-binding EF hand family protein
AT1G65540		signalling.calcium	calcium-binding EF hand family protein
AT5G08580		signalling.calcium	calcium-binding EF hand family protein
AT3G59820		signalling.calcium	calcium-binding mitochondrial protein-related
AT1G70670	AtCLO4	development.unspecified	caleosin-related family protein
AT3G29310		signalling.calcium	calmodulin-binding protein-related
AT2G25310		not assigned.unknown	carbohydrate binding
AT3G62360		not assigned.unknown	carbohydrate binding

AT5G11560		not assigned.no ontology	catalytic
AT5G49900		not assigned.unknown	catalytic/ glucosylceramidase
AT3G52950		not assigned.no ontology	CBS domain-containing protein / octicosapeptide/Phox/Bemp1 (PB1) domain-containing protein
AT1G63110		cell.division	cell division cycle protein-related
AT3G03960		protein.folding	chaperonin, putative
AT3G11830		protein.folding	chaperonin, putative
AT3G13470	Cpn60beta2	protein.folding	chaperonin, putative
AT3G02530		protein.folding	chaperonin, putative
AT3G18190		protein.folding	chaperonin, putative
AT5G20890		protein.folding	chaperonin, putative
AT5G26360		protein.folding	chaperonin, putative
AT4G18060		not assigned.no ontology	clathrin binding
AT3G08530		cell.vesicle transport	clathrin heavy chain, putative
AT3G11130		cell.vesicle transport	clathrin heavy chain, putative
AT4G31490		cell.vesicle transport	coatomer beta subunit, putative / beta-coat protein, putative / beta-COP, putative
AT1G62020		cell.vesicle transport	coatomer protein complex, subunit alpha, putative
AT2G21390		cell.vesicle transport	coatomer protein complex, subunit alpha, putative
AT1G30630		cell.vesicle transport	coatomer protein epsilon subunit family protein / COPE family protein
AT4G33120		lipid metabolism.Phospholipid synthesis.(S)-coclaurine-N-methyltransferase	coclaurine N-methyltransferase, putative
AT3G12260		mitochondrial electron transport / ATP synthesis.NADH-DH.complex I	complex 1 family protein / LVR family protein
AT4G34700	AtCIB22	mitochondrial electron transport / ATP synthesis	complex 1 family protein / LVR family protein
AT1G18420		not assigned.unknown	Uncharacterised protein matching with AtALMT9 (aluminum-activated malate transporter 9)
AT1G53000	AtCKS	cell wall.precursor synthesis.KDO pathway.CMP-KDO Synthetague	cytidylyltransferase family

AT2G47380		mitochondrial electron transport / ATP synthesis.cytochrome c oxidase	cytochrome c oxidase subunit Vc family protein / COX5C family protein
AT3G27240		mitochondrial electron transport / ATP synthesis.cytochrome c	cytochrome c1, putative
AT5G40810		mitochondrial electron transport / ATP synthesis.cytochrome c	cytochrome c1, putative
AT4G21105		not assigned.unknown	cytochrome-c oxidase/ electron carrier
AT1G26850		stress.abiotic.drought/salt	dehydration-responsive family protein
AT4G18030		stress.abiotic.drought/salt	dehydration-responsive family protein
AT1G29470		stress.abiotic.drought/salt	dehydration-responsive protein-related
AT1G04430		stress.abiotic.drought/salt	dehydration-responsive protein-related
AT4G00740	QUA3	stress.abiotic.drought/salt	dehydration-responsive protein-related
AT5G64030		stress.abiotic.drought/salt	dehydration-responsive protein-related
AT5G15910		not assigned.no ontology	dehydrogenase-related
AT5G19760		transport.metabolite transporters at the mitochondrial membrane	dicarboxylate/tricarboxylate carrier (DTC)
AT3G13930		TCA / org transformation.TCA.pyruvate DH.E2	dihydrolipoamide S-acetyltransferase, putative
AT5G67630		DNA.synthesis/chromatin structure	DNA helicase, putative
AT2G24420		DNA.repair	DNA repair ATPase-related
AT2G45820		RNA.regulation of transcription.putative transcription regulator	DNA-binding protein, putative
AT1G21080		stress.abiotic.heat	DNAJ heat shock N-terminal domain-containing protein
AT2G35720	OWL1	stress.abiotic.heat	DNAJ heat shock N-terminal domain-containing protein
AT4G39150		stress.abiotic.heat	DNAJ heat shock N-terminal domain-containing protein
AT1G20575	DPMS1	protein.glycosylation	dolichyl-phosphate beta-D-mannosyltransferase, putative / dolichol-phosphate mannosyltransferase, putative / mannose-P-dolichol synthase, putative
AT5G54830		misc.oxidases - copper, flavone etc	DOMON domain-containing protein / dopamine beta-monooxygenase N-terminal domain-containing protein

AT5G25940		development.unspecified	early nodulin-related
AT5G42150		not assigned.unknown	electron carrier/ protein disulfide oxidoreductase
AT3G08950	HCC1	mitochondrial electron transport / ATP synthesis.cytochrome c oxidase	electron transport SCO1/SenC family protein
AT1G30230		protein.synthesis.elongation	elongation factor 1-beta / EF-1-beta
AT1G57720		protein.synthesis.elongation	elongation factor 1B-gamma, putative / eEF-1B gamma, putative
AT1G09640		protein.synthesis.elongation	elongation factor 1B-gamma, putative / eEF-1B gamma, putative
AT1G69460		transport.misc	emp24/gp25L/p24 family protein
AT3G22845		cell.vesicle transport	emp24/gp25L/p24 protein-related
AT1G10950	AtTMN1	not assigned.no ontology	endomembrane protein 70, putative
AT1G14670		not assigned.no ontology	endomembrane protein 70, putative
AT2G01970		not assigned.no ontology	endomembrane protein 70, putative
AT3G13772	AtTMN7	not assigned.unknown	endomembrane protein 70, putative
AT5G10840		not assigned.unknown	endomembrane protein 70, putative
AT5G25100		not assigned.no ontology	endomembrane protein 70, putative
AT2G25430		not assigned.no ontology.epsin N-terminal homology (ENTH) domain-containing protein	epsin N-terminal homology (ENTH) domain-containing protein
AT2G01600		not assigned.no ontology.epsin N-terminal homology (ENTH) domain-containing protein	epsin N-terminal homology (ENTH) domain-containing protein
AT4G32285		not assigned.no ontology.epsin N-terminal homology (ENTH) domain-containing protein	epsin N-terminal homology (ENTH) domain-containing protein / clathrin assembly protein-related
AT2G39420		not assigned.no ontology	esterase/lipase/thioesterase family protein
AT5G19290		not assigned.no ontology	esterase/lipase/thioesterase family protein
AT1G09740		hormone metabolism.ethylene.induced-regulated-responsive-activated	ethylene-responsive protein, putative
AT1G42960		not assigned.unknown	expressed protein localized to the inner membrane of the chloroplast.
AT3G51050		not assigned.no ontology	FG-GAP repeat-containing protein
AT1G75200		not assigned.no ontology	flavodoxin family protein / radical SAM domain-containing protein

AT2G22900		misc.UDP glucosyl and glucoronyl transferases	galactosyl transferase GMA12/MNN10 family protein
AT3G49360	PGL2	OPP.oxidative PP.6-phosphogluconolactonase	glucosamine/galactosamine-6-phosphate isomerase family protein
AT1G67930		cell.vesicle transport	Golgi transport complex protein-related
AT5G46070		signalling.G-proteins	GTP binding / GTPase
AT3G28710		transport.p- and v-ATPases.H+-transporting two-sector ATPase	H+-transporting two-sector ATPase, putative
AT3G25610		transport.p- and v-ATPases	haloacid dehalogenase-like hydrolase family protein
AT5G44240	ALA2	transport.p- and v-ATPases	haloacid dehalogenase-like hydrolase family protein
AT1G08160		not assigned.no ontology	harpin-induced protein-related / HIN1-related / harpin-responsive protein-related
AT4G35060	HIPP25	metal handling.binding, chelation and storage	heavy-metal-associated domain-containing protein / copper chaperone (CCH)-related
AT4G38350		not assigned.no ontology	hedgehog receptor
AT1G09870		misc.acid and other phosphatases	histidine acid phosphatase family protein
AT5G10980		DNA.synthesis/chromatin structure.histone.core.H3	histone H3
AT5G59970		DNA.synthesis/chromatin structure.histone.core.H4	histone H4
AT3G27325		not assigned.unknown	hydrolase, acting on ester bonds
AT2G36290		not assigned.no ontology	hydrolase, alpha/beta fold family protein
AT3G48410		not assigned.no ontology	hydrolase, alpha/beta fold family protein
AT2G32600		not assigned.no ontology.hydroxyproline rich proteins	hydroxyproline-rich glycoprotein family protein
AT4G23470		not assigned.no ontology.hydroxyproline rich proteins	hydroxyproline-rich glycoprotein family protein
AT5G27760		not assigned.no ontology	hypoxia-responsive family protein
AT2G36100	CASP1	not assigned.no ontology	integral membrane family protein
AT3G11550	CASP2	not assigned.no ontology	integral membrane family protein
AT4G27860		not assigned.no ontology	integral membrane family protein
AT4G27870		not assigned.no ontology	integral membrane family protein
AT4G15610		not assigned.no ontology	integral membrane family protein

AT1G75220	AtERDL6	transport.sugars	integral membrane protein, putative
AT2G38480		not assigned.no ontology	integral membrane protein, putative
AT3G05280		not assigned.no ontology	integral membrane Yip1 family protein
AT3G52760		not assigned.no ontology	integral membrane Yip1 family protein
AT4G30260		not assigned.no ontology	integral membrane Yip1 family protein
AT1G72500		stress.biotic.PR-proteins.proteinase inhibitors.trypsin inhibitor	inter-alpha-trypsin inhibitor heavy chain-related
AT1G19110		stress.biotic.PR-proteins.proteinase inhibitors.trypsin inhibitor	inter-alpha-trypsin inhibitor heavy chain-related
AT5G66060		misc.oxidases - copper, flavone etc	iron ion binding / oxidoreductase/ oxidoreductase, acting on paired donors
AT2G37050		signalling.receptor kinases.leucine rich repeat I	kinase
AT4G08850		signalling.receptor kinases.leucine rich repeat XI	kinase
AT1G54320		not assigned.no ontology	LEM3 (ligand-effect modulator 3) family protein / CDC50 family protein
AT3G23190		stress.biotic	lesion inducing protein-related
AT2G17440	PIRL5	not assigned.no ontology	leucine-rich repeat family protein
AT4G35470	PIRL4	signalling.receptor kinases	leucine-rich repeat family protein
AT5G45510		not assigned.no ontology	leucine-rich repeat family protein
AT5G07910		not assigned.no ontology	leucine-rich repeat family protein
AT1G56140		signalling.receptor kinases.leucine rich repeat VIII.VIII-2	leucine-rich repeat family protein / protein kinase family protein
AT1G56145		signalling.receptor kinases.DUF 26	leucine-rich repeat family protein / protein kinase family protein
AT3G14840		signalling.receptor kinases.DUF 26	leucine-rich repeat family protein / protein kinase family protein
AT3G23750		signalling.receptor kinases.leucine rich repeat IX	leucine-rich repeat family protein / protein kinase family protein
AT5G48380	BIR1	signalling.receptor kinases.leucine rich repeat X	leucine-rich repeat family protein / protein kinase family protein
AT5G49760		signalling.receptor kinases.leucine rich repeat VIII.VIII-1	leucine-rich repeat family protein / protein kinase family protein
AT1G51800	IOS1	signalling.receptor kinases.leucine rich repeat I	leucine-rich repeat protein kinase, putative
AT1G51850		signalling.receptor kinases.leucine rich repeat I	leucine-rich repeat protein kinase, putative

AT1G25570		not assigned.no ontology	leucine-rich repeat protein-related
AT1G06840		signalling.receptor kinases.leucine rich repeat VIII.VIII-1	leucine-rich repeat transmembrane protein kinase, putative
AT1G27190		signalling.receptor kinases.leucine rich repeat X	leucine-rich repeat transmembrane protein kinase, putative
AT2G31880	EVR	signalling.receptor kinases.leucine rich repeat XI	leucine-rich repeat transmembrane protein kinase, putative
AT3G28450		signalling.receptor kinases.leucine rich repeat X	leucine-rich repeat transmembrane protein kinase, putative
AT3G02880		signalling.receptor kinases.leucine rich repeat III	leucine-rich repeat transmembrane protein kinase, putative
AT5G49770		signalling.receptor kinases.leucine rich repeat VIII.VIII-1	leucine-rich repeat transmembrane protein kinase, putative
AT5G37710		signalling.calcium	lipase class 3 family protein / calmodulin-binding heat-shock protein, putative
AT1G67560	ATLOX6	hormone metabolism.jasmonate.synthesis-degradation.lipoxygenase	lipoxygenase family protein
AT2G04350	LACS8	lipid metabolism.FA synthesis and FA elongation.long chain fatty acid CoA ligase	long-chain-fatty-acid--CoA ligase family protein / long-chain acyl-CoA synthetase family protein (LACS8)
AT1G51590	MANIB	protein.glycosylation.mannosyl-oligosaccharide alpha-1,2-mannosidase	mannosyl-oligosaccharide 1,2-alpha-mannosidase, putative
AT4G25640	ATDTX35	transport.misc	MATE efflux family protein
AT4G00290		not assigned.no ontology	mechanosensitive ion channel domain-containing protein / MS ion channel domain-containing protein
AT1G12640		not assigned.no ontology	membrane bound O-acyl transferase (MBOAT) family protein
AT3G07570		not assigned.no ontology	membrane protein, putative
AT5G64500		not assigned.no ontology	membrane protein-related
AT2G16460		not assigned.unknown	metal ion binding
AT2G19080		protein.targeting.mitochondria	metaxin-related
AT2G39960		protein.targeting.secretory pathway.ER	microsomal signal peptidase 25 kDa subunit, putative (SPC25)
AT2G19680		mitochondrial electron transport / ATP synthesis.F1-ATPase	mitochondrial ATP synthase g subunit family protein
AT4G26210		mitochondrial electron transport / ATP synthesis.F1-ATPase	mitochondrial ATP synthase g subunit family protein
AT4G29480		mitochondrial electron transport / ATP synthesis.F1-ATPase	mitochondrial ATP synthase g subunit family protein

AT3G49560		protein.targeting.mitochondria	mitochondrial import inner membrane translocase subunit Tim17/Tim22/Tim23 family protein
AT5G14040	PHT3;1	transport.metabolite transporters at the mitochondrial membrane	mitochondrial phosphate transporter
AT1G51980		protein.targeting.mitochondria	mitochondrial processing peptidase alpha subunit, putative
AT5G01500	TAAC	transport.metabolite transporters at the mitochondrial membrane	mitochondrial substrate carrier family protein
AT5G15640		transport.metabolite transporters at the mitochondrial membrane	mitochondrial substrate carrier family protein
AT4G38540		misc.oxidases - copper, flavone etc	monooxygenase, putative (MO2)
AT4G31340		cell.organisation	myosin heavy chain-related
AT3G03100		mitochondrial electron transport / ATP synthesis.NADH-DH.localisation not clear	NADH:ubiquinone oxidoreductase family protein
AT5G18800		mitochondrial electron transport / ATP synthesis.NADH-DH.localisation not clear	NADH-ubiquinone oxidoreductase 19 kDa subunit (NDUFA8) family protein
AT5G11770		mitochondrial electron transport / ATP synthesis.NADH-DH.localisation not clear	NADH-ubiquinone oxidoreductase 20 kDa subunit, mitochondrial
AT1G79010		mitochondrial electron transport / ATP synthesis.NADH-DH.localisation not clear	NADH-ubiquinone oxidoreductase 23 kDa subunit, mitochondrial (TYKY)
AT1G16700		mitochondrial electron transport / ATP synthesis.NADH-DH.localisation not clear	NADH-ubiquinone oxidoreductase 23 kDa subunit, mitochondrial, putative
AT4G02580		mitochondrial electron transport / ATP synthesis.NADH-DH.localisation not clear	NADH-ubiquinone oxidoreductase 24 kDa subunit, putative
AT5G47890		mitochondrial electron transport / ATP synthesis.NADH-DH.localisation not clear	NADH-ubiquinone oxidoreductase B8 subunit, putative
AT3G18410		mitochondrial electron transport / ATP synthesis.NADH-DH.localisation not clear	NADH-ubiquinone oxidoreductase-related
AT5G52840		mitochondrial electron transport / ATP synthesis.NADH-DH.localisation not clear	NADH-ubiquinone oxidoreductase-related
AT5G14120		development.unspecified	nodulin family protein
AT5G06260		DNA.synthesis/chromatin structure	nucleolar protein-related
AT1G61790		transport.sugars	OST3/OST6 family protein
AT3G11070		not assigned.no ontology	outer membrane OMP85 family protein
AT5G05520		not assigned.no ontology	outer membrane OMP85 family protein

AT2G17720	P4H5	misc.oxidases - copper, flavone etc	oxidoreductase, 2OG-Fe(II) oxygenase family protein
AT3G28480		misc.oxidases - copper, flavone etc	oxidoreductase, 2OG-Fe(II) oxygenase family protein
AT3G15090		not assigned.no ontology	oxidoreductase, zinc-binding dehydrogenase family protein
AT4G13010		misc.oxidases - copper, flavone etc	oxidoreductase, zinc-binding dehydrogenase family protein
AT5G03700		not assigned.no ontology	PAN domain-containing protein
AT4G19410		cell wall.pectin*esterases.acetyl esterase	pectinacetylesterase, putative
AT5G56730		protein.degradation.metalloprotease	peptidase M16 family protein / insulinase family protein
AT5G51740		protein.degradation	peptidase M48 family protein
AT3G66654		cell.cycle.peptidylprolyl isomerase	peptidyl-prolyl cis-trans isomerase cyclophilin-type family protein
AT5G17820		misc.peroxidases	peroxidase 57 (PER57) (P57) (PRXR10)
AT1G49570		misc.peroxidases	peroxidase, putative
AT4G26010		misc.peroxidases	peroxidase, putative
AT5G03080		misc.acid and other phosphatases	phosphatidic acid phosphatase-related / PAP2-related
AT1G17340		signalling.phosphinositides	phosphoinositide phosphatase family protein
AT3G23400	FIB4	cell.organisation	plastid-lipid associated protein PAP / fibrillin family protein
AT1G63830		not assigned.no ontology.proline rich family	proline-rich family protein
AT3G02200		protein.degradation.ubiquitin.proteasom	proteasome family protein
AT2G20790		cell.vesicle transport	protein binding
AT2G40060	CLC2	not assigned.unknown	protein binding / structural molecule
AT2G20760		not assigned.unknown	protein binding / structural molecule
AT3G51890		not assigned.unknown	protein binding / structural molecule
AT2G23770		signalling.receptor kinases.lysine motif	protein kinase family protein / peptidoglycan-binding LysM domain-containing protein
AT1G52540		signalling.kinase.RLCK.receptor like cytoplasmatic kinase X	protein kinase, putative
AT3G46290	HERK1	signalling.receptor kinases.Catharanthus roseus-like RLK1	protein kinase, putative
AT5G50000		signalling.MAP kinases.putative MAP3K, Raf-like	protein kinase, putative

AT3G46280		signalling.receptor kinases.misc	protein kinase-related
AT3G48570		protein.targeting.secretory pathway.unspecified	protein transport protein SEC61 gamma subunit, putative
AT5G50460		protein.targeting.secretory pathway.unspecified	protein transport protein SEC61 gamma subunit, putative
AT2G34250		protein.targeting.secretory pathway.unspecified	protein transport protein sec61, putative
AT3G44190		not assigned.no ontology	pyridine nucleotide-disulphide oxidoreductase family protein
AT4G33070		fermentation.PDC	pyruvate decarboxylase, putative
AT5G56350		glycolysis.plastid branch.pyruvate kinase (PK)	pyruvate kinase, putative
AT4G36750		lipid metabolism.'exotics'(steroids, squalene etc)	quinone reductase family protein
AT1G15530		signalling.receptor kinases.legume-lectin	receptor lectin kinase, putative
AT5G23750		cell.organization.remorin	remorin family protein
AT3G10260		not assigned.no ontology	reticulon family protein
AT1G64090	RTNLB3	not assigned.no ontology	reticulon family protein (RTNLB3)
AT3G07950		protein.degradation.serine protease	rhomboid protein-related
AT1G76400		protein.glycosylation	ribophorin I family protein
AT2G01720		protein.glycosylation	ribophorin I family protein
AT3G49080		protein.synthesis.ribosomal protein.unknown.small subunit.S9	ribosomal protein S9 family protein
AT4G17720		RNA.RNA binding	RNA recognition motif (RRM)-containing protein
AT1G72960		development.unspecified	root hair defective 3 GTP-binding (RHD3) family protein
AT3G05500		not assigned.no ontology	rubber elongation factor (REF) family protein
AT5G04990	ATSUN1	not assigned.no ontology	sad1/unc-84 protein-related
AT2G04940		not assigned.no ontology	scramblase-related
AT1G32050		transport.misc	secretory carrier membrane protein (SCAMP) family protein
AT5G58640		metal handling	selenoprotein-related
AT1G31440		cell.vesicle transport	SH3 domain-containing protein 1 (SH3P1)
AT4G23430	AtTic32-IVa	misc.short chain dehydrogenase/reductase (SDR)	short-chain dehydrogenase/reductase (SDR) family protein

AT4G09750		misc.short chain dehydrogenase/reductase (SDR)	short-chain dehydrogenase/reductase (SDR) family protein
AT3G05230		protein.degradation	signal peptidase subunit family protein
AT5G05670		protein.targeting.secretory pathway.unspecified	signal recognition particle binding
AT4G30600		protein.targeting.secretory pathway.unspecified	signal recognition particle receptor alpha subunit family protein
AT1G53210		signalling.calcium	sodium/calcium exchanger family protein / calcium-binding EF hand family protein
AT1G63010		not assigned.no ontology	SPX (SYG1/Pho81/XPR1) domain-containing protein
AT1G08470	SSL3	secondary metabolism.N misc.alkaloid-like	strictosidine synthase family protein
AT3G51440		secondary metabolism.N misc.alkaloid-like	strictosidine synthase family protein
AT3G57010		secondary metabolism.N misc.alkaloid-like	strictosidine synthase family protein
AT1G70600		protein.synthesis.ribosomal protein.eukaryotic.60S subunit.L27A	structural constituent of ribosome
AT1G16900		protein.postranslational modification	sugar binding / transferase, transferring glycosyl groups
AT1G18260	EBS5	not assigned.no ontology	suppressor of lin-12-like protein-related / sel-1 protein-related
AT1G24510		protein.postranslational modification	T-complex protein 1 epsilon subunit, putative / TCP-1-epsilon, putative / chaperonin, putative
AT4G33360	FLDH	secondary metabolism.flavonoids.dihydroflavonols	terpene cyclase/mutase-related
AT5G21990	OEP61	not assigned.no ontology.tetratricopeptide repeat (TPR)	tetratricopeptide repeat (TPR)-containing protein
AT5G44920		not assigned.no ontology.Toll-Interleukin-Resistance (TIR) domain-containing protein	Toll-Interleukin-Resistance (TIR) domain-containing protein
AT4G00090		development.unspecified	transducin family protein / WD-40 repeat family protein
AT3G20920		not assigned.no ontology	translocation protein-related
AT2G21160		not assigned.no ontology	translocon-associated protein alpha (TRAP alpha) family protein
AT5G14030		not assigned.no ontology	translocon-associated protein beta (TRAPB) family protein
AT5G23575		not assigned.no ontology	transmembrane protein, putative
AT5G54750		transport.misc	transport protein particle (TRAPP) component Bet3, putative

AT4G25220	AtG3Pp2	transport.membrane system unknown	transporter, putative
AT1G06890		not assigned.no ontology	transporter-related
AT1G76880		RNA.regulation of transcription.Trihelix, Triple-Helix transcription factor family	trihelix DNA-binding protein, putative
AT3G62120		protein.aa activation.bifunctional aminoacyl-tRNA synthetase	tRNA synthetase class II (G, H, P and S) family protein
AT5G51220		protein.targeting.unknown	ubiquinol-cytochrome C chaperone family protein
AT4G32470		mitochondrial electron transport / ATP synthesis.cytochrome c reductase	ubiquinol-cytochrome C reductase complex 14 kDa protein, putative
AT5G25450		mitochondrial electron transport / ATP synthesis.cytochrome c reductase	ubiquinol-cytochrome C reductase complex 14 kDa protein, putative
AT5G05370		mitochondrial electron transport / ATP synthesis.cytochrome c reductase	ubiquinol-cytochrome C reductase complex ubiquinone-binding protein, putative / ubiquinol-cytochrome C reductase complex 8.2 kDa protein, putative
AT5G13430		mitochondrial electron transport / ATP synthesis.cytochrome c reductase	ubiquinol-cytochrome C reductase iron-sulfur subunit, mitochondrial, putative / Rieske iron-sulfur protein, putative
AT3G52730		mitochondrial electron transport / ATP synthesis.cytochrome c reductase	ubiquinol-cytochrome C reductase UQCRX/QCR9-like family protein
AT3G11930		hormone metabolism.ethylene.induced-regulated-responsive-activated	universal stress protein (USP) family protein
AT5G62865		not assigned.unknown	unknown protein
AT1G30130		not assigned.unknown	unknown protein
AT1G04280		not assigned.unknown	unknown protein
AT1G33800		not assigned.unknown	unknown protein
AT1G33810		not assigned.unknown	unknown protein
AT1G36050		not assigned.unknown	unknown protein
AT1G47550	SEC3A	not assigned.unknown	unknown protein
AT1G48200		not assigned.unknown	unknown protein
AT1G51630		not assigned.unknown	unknown protein
AT1G52200		not assigned.no ontology	unknown protein

AT1G52320	SDH6	not assigned.unknown	unknown protein
AT1G52780		not assigned.unknown	unknown protein
AT1G53760		not assigned.unknown	unknown protein
AT1G55160		not assigned.unknown	unknown protein
AT1G57610		not assigned.unknown	unknown protein
AT1G65270		not assigned.unknown	unknown protein
AT1G65985		not assigned.unknown	unknown protein
AT1G67350		not assigned.no ontology	unknown protein
AT1G67785		not assigned.unknown	unknown protein
AT1G08480		not assigned.unknown	unknown protein
AT1G72170		not assigned.unknown	unknown protein
AT1G09330		not assigned.unknown	unknown protein
AT1G80890		not assigned.unknown	unknown protein
AT1G18720		not assigned.no ontology	unknown protein
AT1G20430		not assigned.unknown	unknown protein
AT1G22200		not assigned.unknown	unknown protein
AT1G22520		not assigned.unknown	unknown protein
AT1G24310		not assigned.unknown	unknown protein
AT1G27930		not assigned.unknown	unknown protein
AT2G28430		not assigned.unknown	unknown protein
AT2G31490		not assigned.unknown	unknown protein
AT2G35736		not assigned.unknown	unknown protein
AT2G35790		not assigned.unknown	unknown protein
AT2G38550		not assigned.unknown	unknown protein
AT2G40765		not assigned.unknown	unknown protein
AT2G42310		not assigned.unknown	unknown protein

AT2G44870		not assigned.unknown	unknown protein
AT2G45010		not assigned.no ontology	unknown protein
AT2G46540		not assigned.unknown	unknown protein
AT2G47960		not assigned.unknown	unknown protein
AT2G02510		not assigned.unknown	unknown protein
AT2G20820		not assigned.unknown	unknown protein
AT2G21870	MGP1	not assigned.unknown	unknown protein
AT3G27390		not assigned.unknown	unknown protein
AT3G27930		not assigned.unknown	unknown protein
AT3G28720		RNA.regulation of transcription.putative transcription regulator	unknown protein
AT3G43520		not assigned.unknown	unknown protein
AT3G47833	SDH7	not assigned.unknown	unknown protein
AT3G49720		not assigned.unknown	unknown protein
AT3G51580		not assigned.unknown	unknown protein
AT3G57280		not assigned.unknown	unknown protein
AT3G62010		protein.postranslational modification	unknown protein
AT3G07568		not assigned.unknown	unknown protein
AT3G08600		not assigned.unknown	unknown protein
AT3G08610		not assigned.unknown	unknown protein
AT3G01720		not assigned.unknown	unknown protein
AT3G10980		stress	unknown protein
AT3G01130		not assigned.unknown	unknown protein
AT3G02900		not assigned.unknown	unknown protein
AT3G21190		not assigned.unknown	unknown protein
AT4G20150		not assigned.unknown	unknown protein

AT4G22310		not assigned.unknown	unknown protein
AT4G24330		not assigned.unknown	unknown protein
AT4G25770		not assigned.unknown	unknown protein
AT4G27120		not assigned.unknown	unknown protein
AT4G30010		not assigned.unknown	unknown protein
AT4G39690		not assigned.unknown	unknown protein
AT4G00530		not assigned.unknown	unknown protein
AT4G00585		not assigned.unknown	unknown protein
AT4G12590		not assigned.unknown	unknown protein
AT4G16450		not assigned.unknown	unknown protein
AT5G35460		not assigned.unknown	unknown protein
AT5G04830		not assigned.unknown	unknown protein
AT5G42960		not assigned.unknown	unknown protein
AT5G45480		not assigned.unknown	unknown protein
AT5G47570		not assigned.unknown	unknown protein
AT5G51510		not assigned.unknown	unknown protein
AT5G53650		not assigned.unknown	unknown protein
AT5G54440	AtTRS130	not assigned.unknown	unknown protein
AT5G55610		not assigned.unknown	unknown protein
AT5G59613		not assigned.unknown	unknown protein
AT5G62575	SDH7	not assigned.unknown	unknown protein
AT5G08060		not assigned.no ontology	unknown protein
AT5G65207		not assigned.unknown	unknown protein
AT5G65810	CGR3	not assigned.unknown	unknown protein
AT5G65950		not assigned.unknown	unknown protein
AT5G08540		not assigned.unknown	unknown protein

AT5G09270		not assigned.unknown	unknown protein
AT5G09995		not assigned.unknown	unknown protein
AT5G12470		not assigned.unknown	unknown protein
AT5G13560		not assigned.no ontology	unknown protein
AT5G15320		not assigned.unknown	unknown protein
AT5G16280		not assigned.unknown	unknown protein
AT5G16660		not assigned.unknown	unknown protein
AT5G20090		not assigned.unknown	unknown protein
AT5G25265		not assigned.unknown	unknown protein
AT3G27190	UKL2	nucleotide metabolism.salvage.phosphoribosyltransferases.upp	uracil phosphoribosyltransferase, putative / UMP pyrophosphorylase, putative / UPRTase, putative
AT1G76030		transport.p- and v-ATPases.H+-transporting two-sector ATPase.subunit B	vacuolar ATP synthase subunit B / V-ATPase B subunit / vacuolar proton pump B subunit / V-ATPase 57 kDa subunit
AT4G38510		transport.p- and v-ATPases.H+-transporting two-sector ATPase.subunit B	vacuolar ATP synthase subunit B, putative / V-ATPase B subunit, putative / vacuolar proton pump B subunit, putative / V-ATPase 57 kDa subunit, putative
AT3G58730		transport.p- and v-ATPases.H+-transporting two-sector ATPase.subunit D	vacuolar ATP synthase subunit D (VATD) / V-ATPase D subunit / vacuolar proton pump D subunit (VATPD)
AT3G42050		transport.p- and v-ATPases.H+-transporting two-sector ATPase.subunit H	vacuolar ATP synthase subunit H family protein
AT4G20110	BP80-3;1	protein.targeting.secretory pathway.vacuole	vacuolar sorting receptor, putative
AT4G21450		cell.vesicle transport	vesicle-associated membrane family protein / VAMP family protein
AT4G00170		not assigned.no ontology	vesicle-associated membrane family protein / VAMP family protein
AT5G47180		cell.vesicle transport	vesicle-associated membrane family protein / VAMP family protein
AT5G50650		not assigned.no ontology	WD-40 repeat family protein / St12p protein, putative
AT1G68070		protein.degradation.ubiquitin.E3.RING	zinc finger (C3HC4-type RING finger) family protein
